# Strengthening antimicrobial resistance surveillance systems: a scoping review

**DOI:** 10.1186/s12879-023-08585-2

**Published:** 2023-09-11

**Authors:** Phu Cong Do, Yibeltal Alemu Assefa, Suliasi Mekerusa Batikawai, Simon Andrew Reid

**Affiliations:** School of Public Health, Faculty of Medicine, The University of Queensland, Herston, Australia

**Keywords:** Antimicrobial Resistance, AMR, Surveillance, Antimicrobial Resistance Surveillance, Experiences, Strengths, Gaps, AMR scoping review

## Abstract

**Background:**

Antimicrobial resistance (AMR) is an emerging global public health crisis. Surveillance is a fundamental component in the monitoring and evaluation of AMR mitigation endeavours. The primary aim of the scoping review is to identify successes, barriers, and gaps in implementing AMR surveillance systems and utilising data from them.

**Methods:**

PubMed, Web of Science, SCOPUS, and EMBASE databases were searched systematically to identify literature pertaining to implementation, monitoring, and evaluation of AMR surveillance systems. A thematic analysis was conducted where themes within the literature were inductively grouped based on the described content.

**Results:**

The systematic search yielded 639 journal articles for screening. Following deduplication and screening, 46 articles were determined to be appropriate for inclusion. Generally, most studies focused on human AMR surveillance (n = 38, 82.6%). Regionally, there was equal focus on low- and middle-income countries (n = 7, 15.2%) and trans-national contexts (n = 7, 14.5%). All included articles (n = 46, 100.0%) discussed barriers to either implementing or utilising AMR surveillance systems. From the scoping review, 6 themes emerged: capacity for surveillance, data infrastructure, policy, representativeness, stakeholder engagement, and sustainability. Data infrastructure was most frequently discussed as problematic in evaluation of surveillance systems (n = 36, 75.0%). The most frequent success to surveillance system implementation was stakeholder engagement (n = 30, 65.2%).

**Conclusions:**

Experiences of AMR surveillance systems are diverse across contexts. There is a distinct separation of experiences between systems with emerging surveillance systems and those with established systems. Surveillance systems require extensive refinement to become representative and meet surveillance objectives.

**Supplementary Information:**

The online version contains supplementary material available at 10.1186/s12879-023-08585-2.

## Introduction

Antimicrobial resistance (AMR) has been declared a global public health threat that has the potential to jeopardise the foundations of modern medicine and infectious diseases control [1]. Current estimates suggest that AMR is responsible for approximately 700,000 human mortalities per year [2] with the potential for up to 10 million deaths per year by 2050 if effective strategies to reduce resistance are not implemented [2]. Whilst immediate repercussions to human health have been widely recognised as impetus for action [3], the significance of AMR extends to animal and environmental health sectors [4]. The breadth of the issue, thus, necessitates a collaborative approach to address the multi-faceted AMR crisis [5].

The World Health Organization’s (WHO) Global Action Plan (GAP) on AMR was developed to engage the international community in efforts to address the emerging public health crisis [1]. The GAP describes 5 objectives including: (i) improving awareness on AMR through training, education, and communication, (ii) strengthening knowledge and evidence base through surveillance and research, (iii) reducing the incidence of infection through sanitation, hygiene, and infection prevention measures, (iv) optimisation of antimicrobial medicines in human and animal health, and (v) developing an economic case for sustainable investment for new medicines, diagnostic tools, vaccines, and interventions [1]. The basis for these objectives is to facilitate effective policy and stewardship processes to ultimately produce discernible mitigation efforts against AMR [1].

The second objective of the GAP foregrounds surveillance as an integral component to ascertain the status of AMR in various contexts and monitor progress towards control objectives [1]. The role of continuous AMR surveillance facilitates evaluation of AMR stewardship programmes, interventions, and policy efficacy via the generation of evidence [6]. Moreover, the borderless nature of AMR has emphasised the need for continuous global monitoring [7]. International initiatives such as the Global Antimicrobial Resistance and Use Surveillance System (GLASS) have aimed to provide guidance in assembling and standardising data from national AMR surveillance systems to inform future actions [8]. Whilst surveillance has been outlined as a global necessity [7], the current state of surveillance systems vary greatly across national contexts [9], with some having highly structured and effective systems [10] and others with no system or a system under development [8]. The wide variation in the structure and effectiveness of national surveillance systems and the absence of foundational work to facilitate improvement calls for research to better understand the barriers and enablers of national AMR surveillance systems.

The aim of this scoping review is to identify and thematically map published literature describing the implementation or evaluation of national AMR surveillance systems. The objectives of the scoping review are to (1) identify the main thematic categories that are relevant in implementing, utilising, and improving surveillance systems (2) examine reported challenges and successes in utilising surveillance systems, and (3) identify gaps within literature that can be used to design further studies on AMR surveillance systems.

## Methods

The scoping review methodology was utilized based on the guidelines outlined within Arksey and O’Malley [11]. The search strategy was documented using the protocol outlined within the Preferred Reporting Items for Systematic Reviews and Meta-Analyses for Scoping Reviews (PRISMA-ScR) [12].

### Search strategy

The electronic databases of PubMed, Web of Science, EMBASE and Scopus were searched for full text academic literature. The following keyword search terms were used to identify relevant literature: (“Antimicrobial Resistance Surveillance” OR “AMR Surveillance” OR “Antibiotic Surveillance” OR “Antibiotic Use Surveillance” OR “Antimicrobial Use Surveillance” OR “AMU Surveillance”) AND (Implement* OR Assess* OR Evaluat* OR Challeng* OR Success*). The search was performed on 13/04/2022. All texts searched were published in English. No filtering processes were applied for geographic location or date of publication. Articles were limited to academic journal articles. Citations found through the search process were exported to Endnote and deduplicated. Identified articles were then subjected to title and abstract screening by PD and SB in Endnote to identify relevancy to the scoping review’s objectives.

### Eligibility criteria

The following eligibility criteria for articles are presented in Table 1.

**Table 1:** Inclusion and exclusion criteria for identified articles

Inclusion Criteria	Exclusion Criteria
• Articles focused on antimicrobial resistance/antimicrobial usage surveillance systems. • The article must evaluate successes and/or challenges of implementing the AMR surveillance system. • No limit on article types nor species (both human and animal health) • Language restricted to English	• Articles that focus solely on antimicrobial resistance prevalence in clinical/community/environmental settings • Articles that focused on genomics of microbes, physiological pathways of AMR, diagnostic testing, and epidemiology of AMR without mention of surveillance system • Secondary studies (systematic, rapid, umbrella reviews) were excluded.

### Data extraction, characterisation, and analysis

**Fig. 1:**
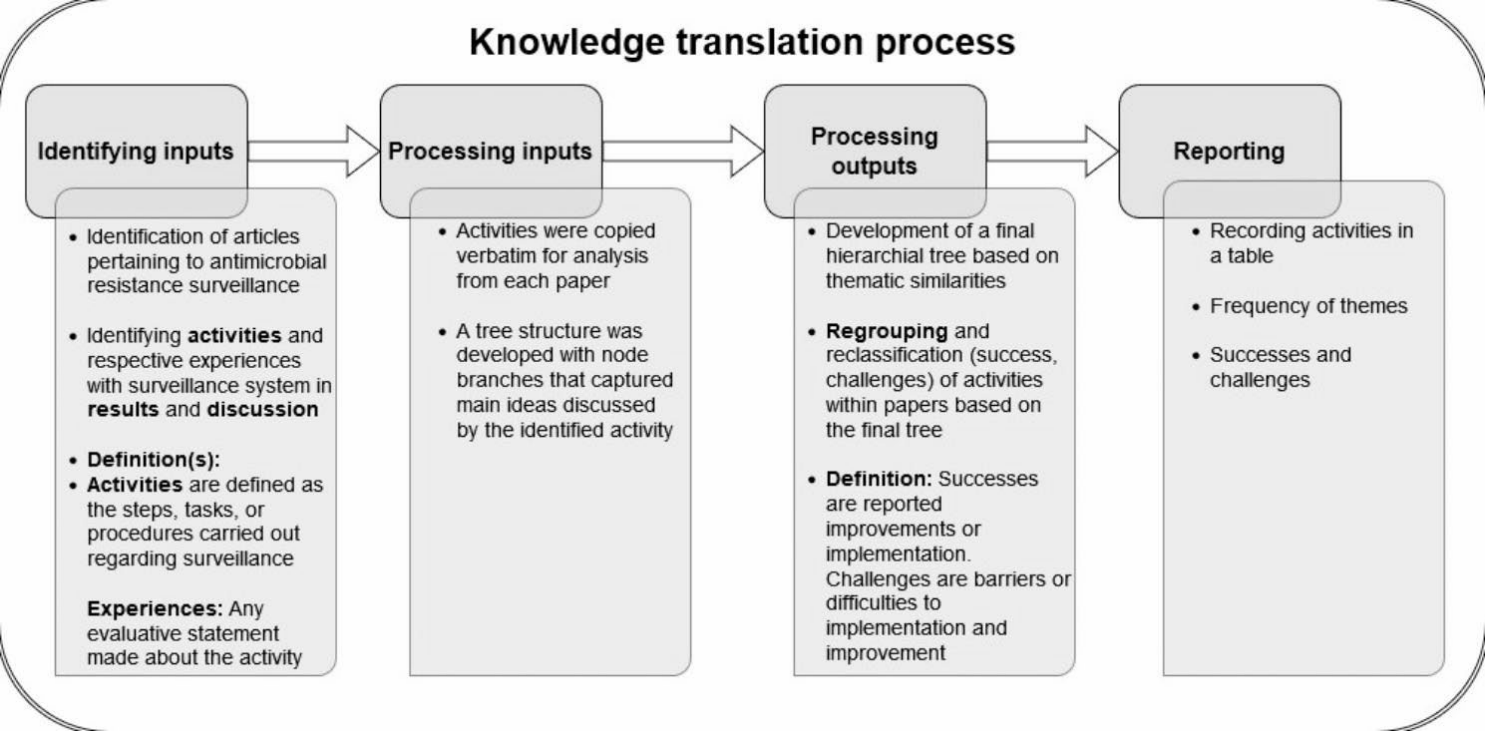
Conceptual framework for the thematic analysis

The full conceptual framework for the review is presented in Fig. 1. Two authors PD and SB independently performed the data extraction on the final articles included in the scoping review. A custom data extraction form was created in Microsoft Excel 2013 for data charting. The form included author, year, title, publication type, geographical location of the article, summary of the article, challenges, and successes detailed within the article. The data extraction process was undertaken continuously and the charted data was analysed using an inductive thematic approach outlined by Thomas and Harden [13]. Activities were defined as steps, tasks, or procedures carried out that were fundamental to the surveillance systems function. Experiences were any evaluative statements made regarding the activities.

An initial text-based analysis was undertaken within each included article to identify initial codes for thematic analysis. PD and SB read through all included articles and then identified the key concepts embodied by each activity or experience. The reviewers looked for identified activities and evaluative statements within the article by the authors to base the analysis. This was undertaken to construct initial codes through extracting the core concept being discussed within the identified text. The initial codes were reviewed by both reviewers and finalised before aggregation by conceptual similarity into sub-themes. Main themes were developed upon further aggregation of the sub-themes identified by their overarching conceptual similarities.

Finally, the activities and experiences were then categorised. Categorisation concerned two elements and was done on the sentiment expressed in each publication. The first was if the activity was a challenge or success to utilisation, implementation, or improvement to surveillance. Successes were defined as activities, experiences, or stipulations that were necessary to overcoming barriers or to facilitate the improvement of surveillance. Challenges were defined as activities or experiences that presented limitations to the improvement or function of surveillance. Secondly, the experiences were categorised by AMR surveillance system status which pertained to the establishment of the surveillance system. Established surveillance systems were identified by reference in the text to a demonstrated track record of practical implementation for an extended period. Conversely, non-established surveillance systems referred to relatively recent or limited implementation of a system which have been relative novelty associated in the implementation. All visualisations and descriptive statistics pertaining to the articles were completed through R 4.6.10 using the *ggplot2* package.

## Results

**Fig. 2:**
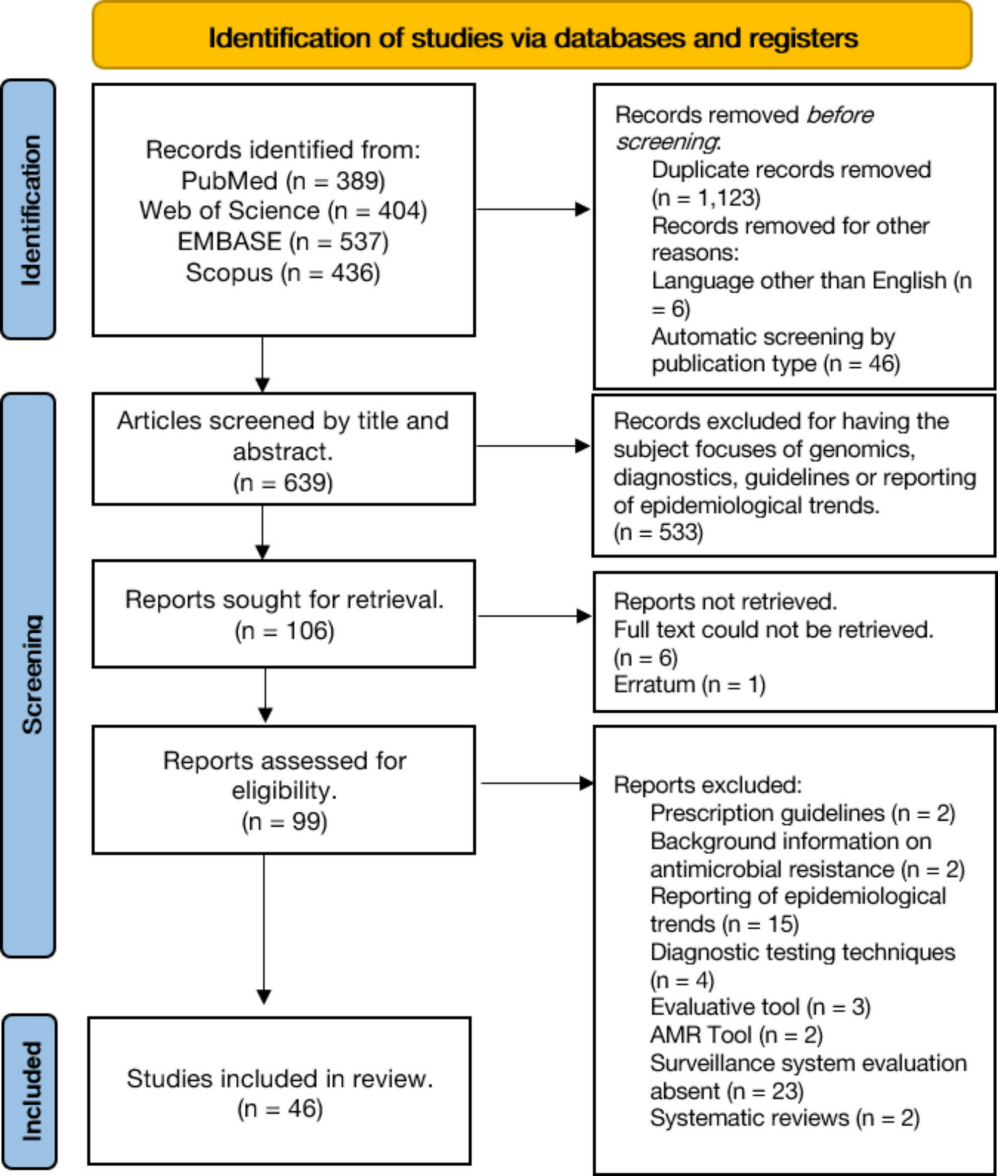
The PRISMA diagram for the scoping review of antimicrobial surveillance system experiences

The search yielded 639 unique articles for review. Following deduplication and screening for relevancy, 99 journal articles met the initial eligibility criteria. Six articles were not included as full text could not be retrieved. One article was a correction statement and was excluded. Two articles were secondary review articles and were excluded from the included studies. After systematically applying the selection criteria to the included articles, a total of 46 articles met the criteria and were included in the final qualitative synthesis of evidence. The flowchart of article selection with exclusion criteria are presented in Fig. 2.

### General characteristics of articles included in scoping review

The general characteristics of included literature are presented in Table 2. Reviews (n = 21, 45.7%) and primary research papers (n = 9, 19.6%) were the most common study type included. There was a single commentary paper and a single narrative review. All articles discussed challenges in utilisation or implementing AMR surveillance systems. Three articles did not outline potential successes in implementing or utilising AMR systems. Most included studies concerned human AMR surveillance (n = 38, 82.6%) with only two studies discussing both human and animal surveillance through a One Health perspective. The main body of included studies was published between 2016 and 2017 (n = 37, 80.4%) with an overall increasing trend of publication from the year 2000.

**Table 2:** Characteristics of included studies in the literature review

Characteristic	Number	Percentage of total% (n = 46)
**Study Type**		
Primary research paper	9	19.6%
Commentary	1	2.2%
Conceptual Analysis	3	6.5%
Cross-sectional	6	13.0%
Editorial	2	4.3%
Field Study	2	4.3%
Narrative Review	1	2.2%
Perspective	3	6.5%
Review	19	41.3%
**Sector**		
Animal	7	15.2%
Human	37	80.4%
Human and Animal	2	4.3%
**Year of publication**		
2000–2005	1	2.2%
2006–2010	3	6.5%
2011–2015	7	15.2%
2016–2021	35	76.1%
**Scope of Articles**		
Challenges	46	100.0%
Successes	43	93.5%

### Geographical characteristics of articles included in scoping review

**Fig. 3:**
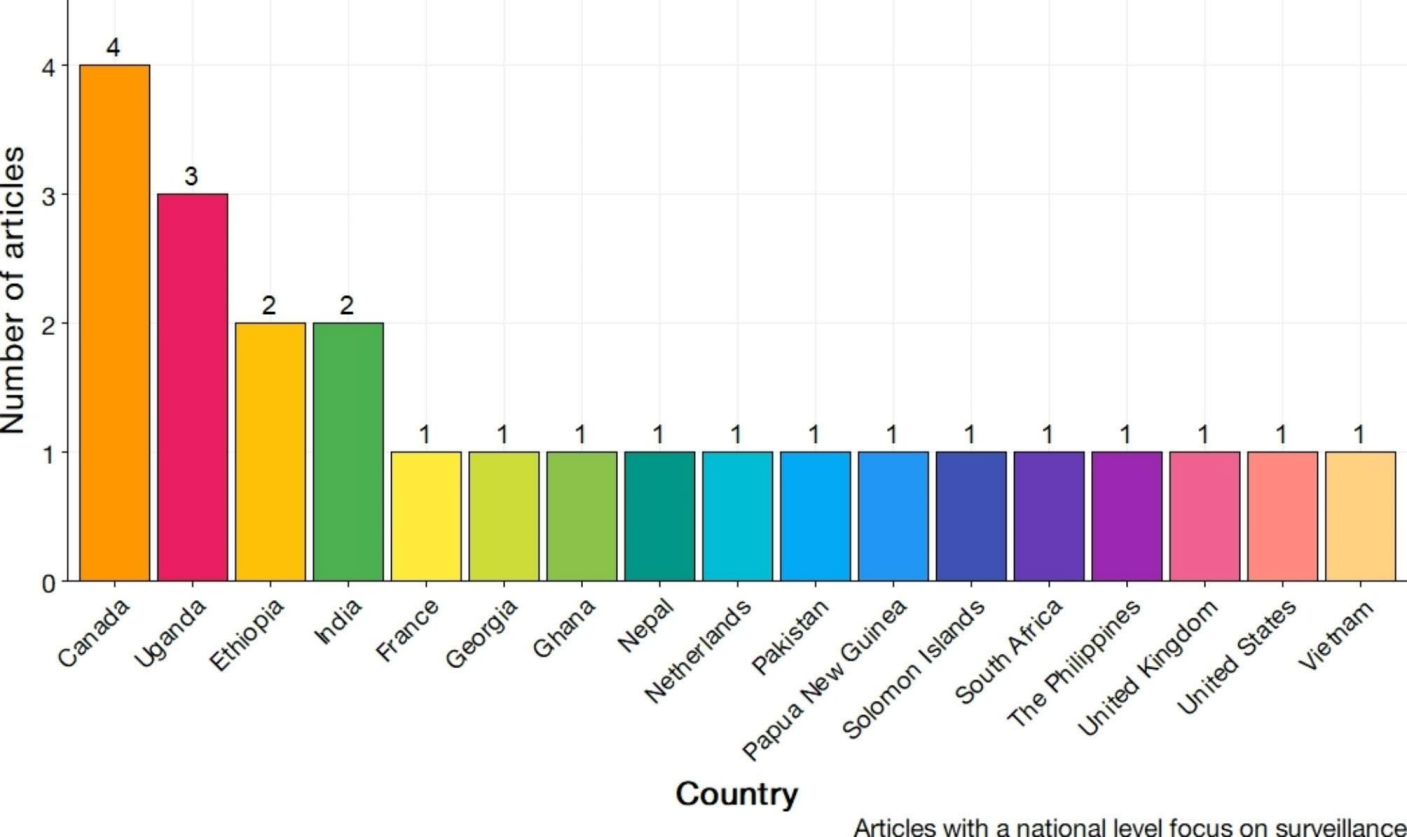
Frequency distribution of articles by national context focus

**Fig. 4:**
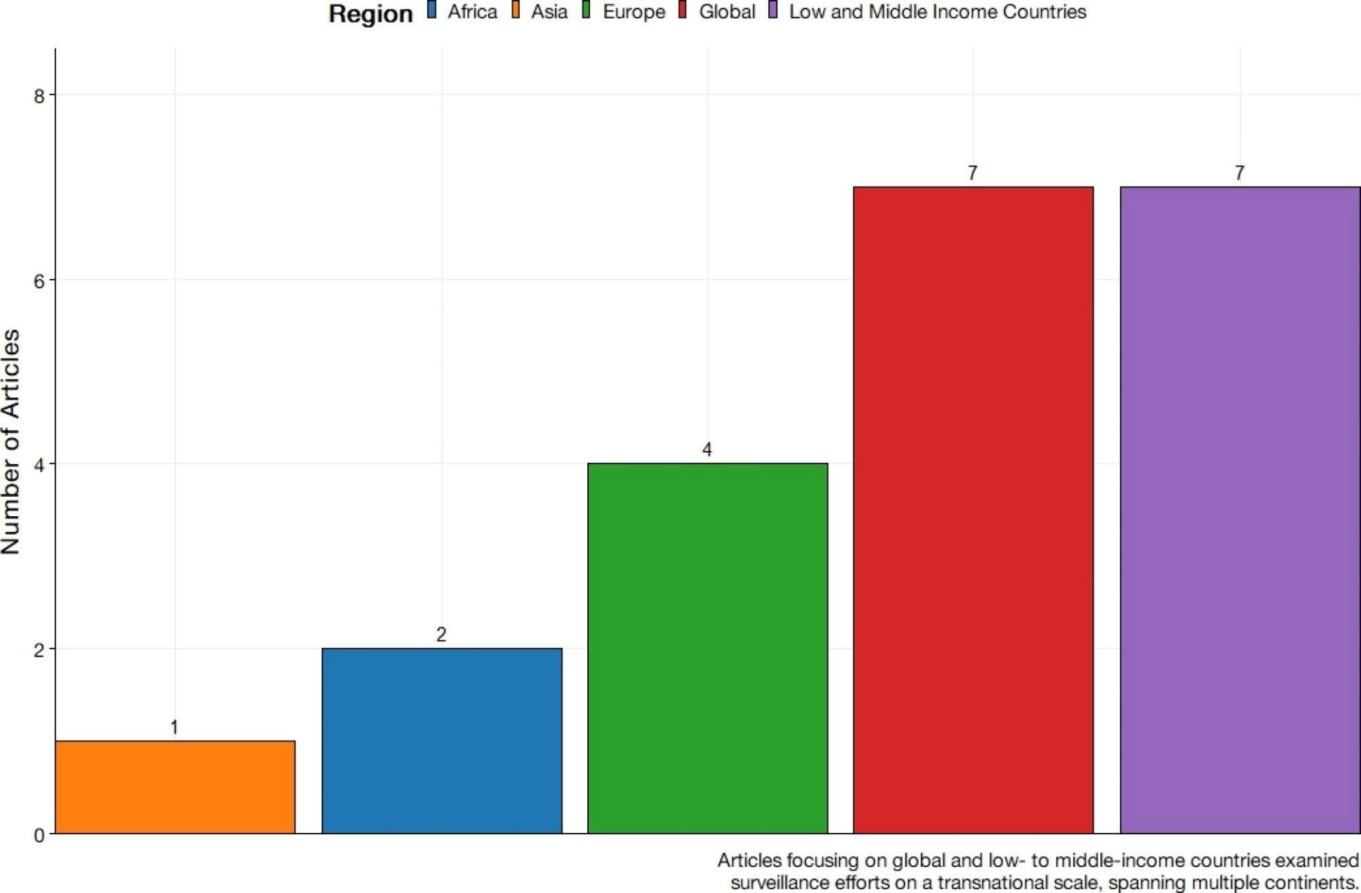
Frequency distribution of studies focused on surveillance at regional levels

Figure 3 depicts the frequency of national contexts within the included literature. More than half of the included literature described a specific regional focus (n = 25, 54.3%). The remainder of the literature examined national, context specific surveillance systems. The most common national context was Canada (n = 4, 8.7%) followed by Uganda (n = 3, 7.5%). Figure 4 presents the frequency of included literature aggregated into transnational contexts. The most frequently observed transnational contexts in literature were that of low- and middle-income countries (LMIC) (n = 7, 14.5%) and global contexts (n = 7, 14.5%). Articles with a global context thematically focus on concerted surveillance activities spanning multiple continents.

### Thematic characteristics of the literature

Six encompassing themes emerged from the included literature. Supplementary file 1 contains the text-based analysis of the individual components of literature that form the initial codes. The themes could be aggregated into physical capacity for surveillance, data infrastructure, policy, stakeholder engagement, sustainability, and representativeness of the system. The activities identified in the included literature were then further classified as successes or challenges. Table 3 presents the frequency of successes and challenges stratified by the respective theme. Overall, data infrastructure was the most frequent challenge experienced within the included literature (n = 36, 78.3%). This was followed by capacity (n = 28, 60.9%). In experiences discussing success for AMR surveillance, stakeholder engagement was the most prominent theme (n = 30, 65.2%). Policy was the least discussed aspect amongst all included literature. Table 4 presents the themes discussed within each article included in the scoping review alongside its characteristics. Table 5 provides the sub-themes which were then aggregated to produce the encompassing themes. The aggregation of individual codes to larger encompassing themes is supplied within is included in supplementary file 3.

**Table 3:** Summary of antimicrobial resistance surveillance system themes in literature with percentage of articles

Theme	Challenges (n, %)	Successes (n, %)
Capacity	28 (60.9%)	13 (28.3%)
Data infrastructure	35 (76.1%)	27 (58.7%)
Policy	1 (2.2%)	7 (15.2%)
Representativeness	17 (37.0%)	9 (19.6%)
Stakeholder engagement	13 (28.3%)	35 (76.1%)
Sustainability	12 (26.1%)	8 (17.4%)

**Table 4:** Characteristics and themes identified within articles

Author	Year	Study Design	Location	Data collection method	Population	Theme					
						Capacity	Data infrastructure	Policy	Representativeness	Stakeholder engagement	Sustainability
Acharya et al. [31]	2021	Cross-sectional	Nepal	Structured Questionnaire	Human	✓	✓				
Altorf-van der Kuil et al. [55]	2017	Perspective	Netherlands	Secondary records and working documents	Human		✓		✓	✓	
Argimón et al. [50]	2020	Cross-sectional	The Philippines	Secondary records and working documents	Human	✓	✓			✓	
Ashley et al. [32]	2019	Perspective	LMIC^a^	Opinion	Human		✓		✓	✓	
Bala et al. [33]	2010	Cross-sectional	India	Secondary records and working documents	Human				✓	✓	
Bennani [53]	2021	Review	United Kingdom	Secondary records and working documents	Human		✓		✓	✓	
Burns et al. [52]	2018	Review	Canada	Stakeholder interview	Human	✓	✓		✓		
Chandrasekera et al. [34]	2015	Review	United States	Secondary records and working documents	Human		✓			✓	
Chandy et al. [70]	2013	Cross-sectional	India	Secondary records and working documents	Human	✓	✓			✓	
Collineau [56]	2019	Review	Global	Secondary records and working documents	Human	✓	✓			✓	
Deckert et al. [35]	2010	Cross-sectional	Canada	Secondary records and working documents	Animal		✓		✓		
Ferguson et al. [20]	2020	Field study	Solomon Islands and Papua New Guinea	Secondary records and working documents	Human	✓				✓	
Fluit et al. [16]	2006	Review	Europe	Secondary records and working documents	Human and animal	✓	✓		✓	✓	
Frost et al. [45]	2021	Review	Global	Secondary records and working documents	Human		✓		✓	✓	✓
Gandra et al. [17]	2020	Review	Asia	Secondary records and working documents	Human	✓	✓		✓		✓
Hannon et al. [14]	2020	Conceptual analysis	Canada	Secondary records and working documents	Animal	✓	✓	✓		✓	✓
Hazim et al. [24]	2018	Review	Ethiopia	Secondary records and working documents	Human	✓	✓			✓	
Hedman, Vasco and Zhang [15]	2020	Review	LMIC^a^	Secondary records and working documents	Animal	✓	✓				✓
Ibrahim et al. [25]	2018	Field study	Ethiopia	Secondary records and working documents	Human	✓	✓				✓
Iskandar et al. [21]	2021	Review	LMIC^a^	Secondary records and working documents	Human	✓	✓	✓			✓
Kakooza et al. [36]	2021	Review	Uganda	Secondary records and working documents	Human		✓			✓	
Kariuki et al. [37]	2018	Editorial	Africa	Secondary records and working documents	Human	✓					✓
Kaur et al. [49]	2019	Conceptual analysis	India	Concept	Human		✓				
Léger et al. [38]	2011	Conceptual analysis	Canada	Concept	Animal		✓		✓	✓	
Lim et al. [47]	2021	Narrative review	LMIC^a^	Narrative Review	Human	✓	✓		✓		
Mader et al. [26]	2021	Perspective	Europe	Perspective	Animal		✓	✓	✓		✓
Mader et al. [54]	2021	Article	France	Secondary records and working documents	Animal	✓	✓		✓	✓	
Malania et al. [18]	2020	Review	Georgia	Secondary records and working documents	Human	✓	✓	✓		✓	✓
Malla et al. [39]	2014	Review	Nepal	Secondary records and working documents	Human	✓	✓			✓	✓
Mitchell et al. [51]	2020	Article	Vietnam	Interview	Human and animal	✓	✓			✓	
Monnet [40]	2000	Review	Europe	Secondary records and working documents	Human		✓			✓	
Mugerwa et al. [27]	2021	Review	Uganda	Secondary records and working documents	Human	✓			✓	✓	
Nabadda et al. [41]	2021	Cross-sectional	Uganda	Secondary records and working documents	Human	✓	✓			✓	
Opintan et al. [28]	2015	Article	Ghana	Secondary records and working documents	Human	✓	✓		✓	✓	
Perovic and Schultsz [23]	2016	Editorial	Africa	Editorial	Human	✓	✓		✓	✓	
Queenan, Häsler, and Rushton [22]	2016	Review	Global	Secondary records and working documents	Human	✓	✓			✓	✓
Rattanaumpawan et al. [48]	2018	Article	LMIC^a^	Secondary records and working documents	Human	✓	✓				
Rempel, Pitout and Laupland [57]	2011	Article	Global	Secondary records and working documents	Human				✓		
Saeed et al. [42]	2017	Article	Pakistan	Secondary records and working documents	Human	✓	✓			✓	
Seale et al. [29]	2017	Article	LMIC^a^	Secondary records and working documents	Human	✓	✓	✓		✓	
Seale et al. [43]	2017	Article	LMIC^a^	Secondary records and working documents	Human	✓	✓	✓			✓
Simjee et al. [44]	2018	Review	Global	Secondary records and working documents	Human		✓		✓	✓	
Singh-Moodley, Ismail, and Perovic [46]	2018	Review	South Africa	Secondary records and working documents	Human	✓	✓	✓		✓	✓
Spiteri [30]	2013	Article	Europe	Secondary records and working documents	Human	✓	✓		✓	✓	
Tornimbene et al. [71]	2018	Commentary	Global	Commentary	Human		✓				
Vernet et al. [19]	2014	Review	LMIC^a^	Secondary records and working documents	Human	✓	✓	✓		✓	

^a^ LMIC = Low- and middle-income countries;

**Table 5:** Thematic characteristics of the included literature with their respective sub-themes and constituent activities which lead to the formation of the themes

Theme	Outcome^a^	Sub-theme	Description	Number of articles	References
**Capacity **	**Challenges** **(n = 18)**	Physical staffing and training	Inadequate trained staff	3	[14, 20, 33]
			Inadequate training new technology	1	[23]
			Insufficient training for diagnostic microbiology and data collection	5	[26, 28, 29, 31, 41, 59]
		Inadequate laboratory infrastructure	Absence of commitment from management and overall limited health system capacity	2	[35, 54]
			Electricity and water supply	5	[25, 28, 33, 48, 49]
			Limited specimen transport	2	[38, 59]
			Software for laboratory	1	[22]
	**Success** **(n = 13)**	Training programmes	External programme enrolment	2	[26, 41]
			Laboratory mentorship	8	[25, 30, 32, 39, 45, 47, 53, 57]
		Laboratory network	National reference laboratory	3	[28, 46, 48]
			Laboratory network structure	1	[52]
		Incentives	Stewardship incentives for microbiological services	1	[38]
**Data Infrastructure**	**Challenges** **(n = 35)**	Quality Assurance	Emphasis on quality assurance	24	[14, 17, 18, 21, 24, 26, 28, 30, 33, 34, 35, 37, 41, 42, 44, 45, 46, 49, 52, 53, 54, 55, 57]
		Non-standardised methods	Diagnostic criteria	10	[21, 27, 28, 29, 39, 44, 49, 55, 56, 59]
			Reporting of bacterial species	3	[14, 31, 39]
			Testing of antimicrobials	2	[39, 44]
			Meta data collection	4	[20, 27, 34, 44]
			Data collection methods	2	[27, 30]
			Information management systems	7	[24, 27, 29, 30, 32, 33, 56]
			Limited pre-existing data	1	[23]
		Data capture	Restricted access to repositories	2	[15, 17]
			Slow retrieval of susceptibility results	1	[21]
			Poor data reporting	3	[22, 36, 54]
			Inadequate data feedback	1	[16]
	**Successes** **(n = 27)**	Standardisation	WHONET system for data collection	10	[34, 38, 41, 44, 46, 47, 48, 49, 50]
			Centralised coordination centre	2	[21, 30]
			Data capture standardisation	3	[33, 39, 42]
		External programmes	Global Antimicrobial Resistance and Use Surveillance System (GLASS)	3	[46, 56, 58]
			European Antimicrobial Resistance Surveillance Scheme (EARSS)	1	[59]
			Clinical standards	3	[21, 41, 55]
		Linkage of data systems	Linkage of surveillance data to antibiotic consumption	1	[26]
			Additional data sources	4	[16, 20, 36, 43]
			Electronic messaging	1	[20]
**Policy**	**Success** **(n = 7)**	Supporting surveillance implementation	Strengthening data infrastructure	2	[29, 53]
			Securing resources for surveillance	1	[56]
			Legal foundations	4	[33, 39, 41, 59]
	**Challenges** **(n = 1)**	Absence of policy	Developing policy	1	[54]
**Representativeness**	**Challenges** **(n = 17)**	Limited representativeness	Inadequate meta-data to categorise antimicrobial resistance	4	[15, 19, 20, 24]
			Over-representation of sites	2	[28, 47]
			Limited coverage by the surveillance system	2	[26, 39]
			Heterogenous surveillance system focus	2	[26, 57]
			Absence of multi-disciplinary steering committee	1	[40]
		Incomplete data collection	Limited sources for data	2	[24, 37]
			Reporting bias in data collection	3	[26, 38, 48]
			Inadequate indicators for surveillance data	1	[27]
			Incorporation of multi-disciplinary team for surveillance coverage in animal health	1	[40]
	**Successes** **(n = 9)**	Improving breadth of data sources	Financial incentives	2	[17, 37]
			External programmes	2	[21, 26]
			Field laboratories	1	[40]
			Increased participation into surveillance system	1	[15]
		Proposed strategies	Harmonisation of procedures and data sources	2	[45, 51]
			Diagnostic cycle for laboratory-based infectious disease surveillance	1	[48]
**Stakeholder engagement**	**Challenges (n = 13)**	Difficulty in coordination	Coordinating laboratory networks	1	[47]
			Engagement of government bodies	5	[14, 31, 33, 41, 43]
			Poor linkage between human, animal, and environmental health sectors	1	[27]
			Engaging clinicians and facilities to participate in surveillance	1	[22]
		Inadequate feedback mechanisms	Difficulty in disseminating results	3	[26, 29, 40]
		Engagement of staff	Engagement with staff to ensure standardised operating procedure	1	[25]
			Staff education to facilitate participation in surveillance	1	[21]
	**Successes** **(n = 34)**	Engagement of stakeholders	External stakeholders establish/improve surveillance laboratory networks	21	[15, 17, 18, 19, 25, 30, 32, 34, 37, 40, 42, 43, 45, 46, 48, 53, 54, 55, 56]
			Participation in a consortium	7	[23, 26, 27, 29, 47, 57, 59]
			Engaging laboratory leadership to facilitate participation in staff training	3	[18, 30, 52]
		Incorporation of external stakeholders	Private sector to increase data sources	2	[17, 28]
		One Health^b^ approach	Necessity for engagement across animal, human, and environmental health sectors	2	[27, 48]
**Sustainability**	**Challenges** **(n = 12)**	Limited funding of surveillance	Under funding of surveillance	4	[26, 27, 42, 56]
			Concurrent funding for sustained surveillance	2	[28, 41]
			Reliance on external funding	2	[28, 33]
		Costs of surveillance	High cost for isolate screening	1	[31]
			Cost of setting up surveillance	1	[49]
			Cost benefit of surveillance	1	[29]
	**Successes (n = 9)**	Funding	External funding to establish and improve surveillance systems	5	[27, 33, 35, 41]
		Partnerships to address financial limitations	Agency partnership for laboratory supplies	1	[32]
			Government led initiatives	1	[34]
			One Health as an economic case	1	[49]
			Gradual development of surveillance networks	1	[39]

^a^ Number of articles reported in outcome represents unique articles.

^b^ One Health refers to the transdisciplinary approach emphasising interconnectedness between human, animal, and environmental health sectors.

#### Capacity

Capacity emerged as a theme that encompassed the physical components of AMR surveillance. This includes staffing and associated training, materials for laboratory diagnostics, physical infrastructure, and transportation. Sub-themes for the identified challenges include physical staffing and training and inadequate laboratory infrastructure. The most frequent challenges within literature pertained to insufficient training in diagnostic microbiology and data collection [14–19] and electricity and water supply [17, 20–23]. Sub-themes for success identified training programmes, the use of laboratory networks, and incentives to improve surveillance. Within successes, the most frequent activity concerned the use of laboratory mentorship in training programmes to ameliorate challenges with training and data collection [20, 24–30].

### Data infrastructure

Data infrastructure was constructed as a theme which discussed and evaluated characteristics data capture. This includes quality assurance, linkage, and methods for data collection. For the challenges pertaining to data infrastructure, literature highlighted the subthemes of quality assurance, non-standardised methods, and data capture to be problematic. Quality assurance of AMR data was emphasised as a prominent challenge in the included literature [16–18, 21, 22, 24, 27, 29–44]. This was followed by differences in diagnostic criteria for AMR across surveillance contexts [14, 17, 19, 22, 26, 34, 40, 44–46]. Successes were achieved through standardisation, external programme enrolment, and linkage of data systems. Experiences discussing successes overwhelmingly highlighted the WHONet system for data collection as a means for data standardisation [18, 22, 23, 28, 36, 40, 41, 47, 48]. Other notable successes included sourcing additional data and linking new sources [49–52].

### Policy

Policy was identified as a theme that discussed legislative foundations for surveillance systems. Of all themes identified within literature, policy was the least frequent. The theme was split by successes in which policy supported implementation or there was a noted absence as a challenge to utilisation of AMR surveillance. The policy experiences mostly detailed successful experiences. Legal foundations for surveillance were the most prominent activity discussed in 4 articles [18, 19, 21, 26]. Only one article discussed a policy related challenge in currently developing policy [43].

### Stakeholder engagement

Stakeholder engagement refers to the success and challenges in the active involvement and collaboration of various relevant individuals, organisations, and entities with vested interests in the context of AMR surveillance. The sub-themes compromising stakeholder engagement challenges were focused on difficulty in coordination, inadequate feedback mechanisms to stakeholders and engagement of staff. Most notably, engaging governmental bodies was the most prominent notion discussed [15, 18, 21, 31, 51]. Successes detailed cases of successful engagement of stakeholders, incorporating stakeholders not previously considered, and plans for a One Health approach to AMR surveillance. Overwhelmingly, much of the included literature highlighted cases were engagement of stakeholders improved surveillance [20, 23–25, 27, 29, 32, 33, 36, 38, 39, 41, 43, 44, 46, 51, 53–55] or a consortium was formed to achieve similar results [14, 16, 19, 28, 30, 45, 56].

### Sustainability

Sustainability encompassed the discussion of economic elements that were necessary to the function of surveillance. The relevant sub-themes for challenges highlighted limited funding and costs of surveillance to be problematic. Most notably, under funding of surveillance [16, 39, 45, 46] and reliance on external funding were two prominent experiences detailed [17, 21]. Successes in sustainability identified securing funding and financial partnerships to be pivotal in successes. Sourcing external funding to establish surveillance was overwhelmingly discussed by literature [18, 21, 37, 45]. Partnerships to address financial limitations was varied in the relationships formed.

### Representativeness

Representativeness refers to the degree in which the AMR data gathered from surveillance accurately reflects the true patterns and trends of AMR within its specified context. The sub-themes identified within the literature include limited representativeness and incomplete data collection. Inadequate meta-data to categorise AMR was the most frequently discussed challenge [35, 52, 53, 55]. The next frequently discussed experience was reporting bias in data collection [16, 23, 47]. Successes focused on improving the breadth of data sources encompassed by the surveillance system and stipulated strategies for improved representativeness. Financial incentives [32, 38], external programmes [16, 34], and harmonisation of procedures and data sources [27, 57] were equally as prominent within literature.

### Separation of experiences

The analysis of included literature highlighted a prominent separation in experiences. Table 6 displays the categorisation of all included literature. Non-established surveillance system literature more frequently originated from LMIC contexts as compared to established surveillance systems. Most notably, representativeness was more frequently discussed in countries with established AMR surveillance systems (n = 15, 78.9%) as compared to non-established (n = 7, 36.8%). Sustainability was more frequently discussed in literature describing non-established AMR surveillance systems (n = 11, 64.7%) as compared to established systems (n = 8, 47.1%). Data infrastructure and stakeholder engagement were similar in their frequency of discussion across established and non-established.

**Table 6:** Themes discussed by established and non-established surveillance systems

Theme (n)	Surveillance System Status	Articles^a^
Capacity (n = 29)	Non-established (n = 17, 58.6%)	[14, 25, 28, 30, 32, 33, 35, 38, 41, 45, 47, 48, 50, 52, 53, 54, 59]
	Established (n = 21, 72.4%)	[16, 20, 22, 23, 26, 28, 29, 31, 40, 42, 43, 49, 53, 56]
Data infrastructure (n = 39)	Non-established (n = 24, 61.5%)	[14, 17, 22, 27, 28, 30, 33, 34, 36, 41, 46, 48, 49, 50, 52, 53, 54, 55, 58, 59]
	Established (n = 25, 64.1%)	[15, 16, 19, 20, 21, 23, 24, 26, 27, 28, 29, 31, 32, 37, 39, 40, 42, 43, 44, 49, 55, 56, 57, 58]
Policy (n = 8)	Non-established (n = 6, 75.0%)	[33, 41, 53, 54, 56, 59]
	Established (n = 2, 25.0%)	[29, 39]
Stakeholder Engagement (n = 36)	Non-established (n = 23, 63.9%)	[15, 17, 25, 27, 28, 30, 32, 33, 34, 41, 42, 46, 47, 48, 49, 52, 53, 54, 56, 59]
	Established (n = 20, 55.6%)	[14, 16, 18, 19, 21, 23, 26, 27, 28, 29, 31, 37, 39, 40, 42, 43, 44, 49, 55, 57]
Sustainability (n = 17)	Non-established (n = 11, 64.7%)	[17, 22, 27, 28, 32, 33, 34, 35, 41, 42, 54, 56]
	Established (n = 8, 47.1%)	[26, 27, 28, 29, 31, 35, 39, 49]
Representativeness (n = 19)	Non-established (n = 7, 36.8%)	[17, 27, 28, 33, 47, 48, 51]
	Established (n = 15, 78.9%)	[15, 18, 19, 20, 21, 24, 26, 27, 28, 37, 39, 40, 51, 55, 57]

^a^ Articles included may discuss multiple surveillance systems across both levels of establishment.

**Fig. 5:**
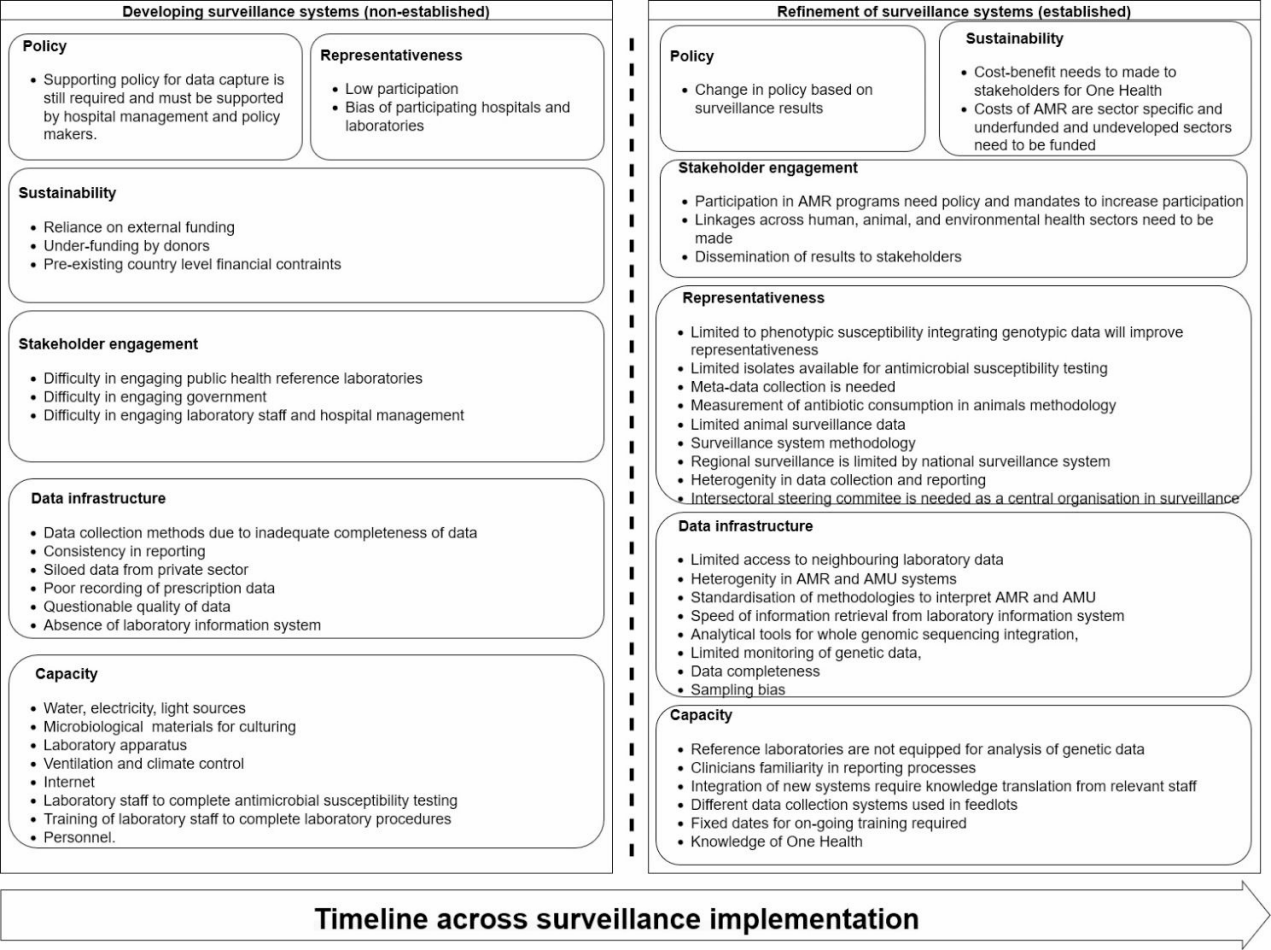
Challenges identified in themes across literature categorised by new and established surveillance systems

Moreover, the divergence of experiences has been elucidated in Fig. 5. Figure 5 provides a visual representation of the divergence in challenges across all reviewed literature by their relative establishment. Discussion in literature with non-established systems more frequently pertained to LMICs. A paradigm shift emerged which is categorised the transition from physical barriers being experienced by LMIC to conceptual barriers being attributable to higher income countries. Physical capacity for surveillance emerges as a greater theme to developing surveillance systems, whereas representativeness of the system becomes more apparent with the establishment of the system. Particularly, for higher income countries it becomes the desire for new technology integration becomes more pronounced. Sustainability for non-established surveillance systems primarily centres around sourcing external funding which contrasts a renewed focus on sustainable internal funding by governments and organisations with established systems. Policy and stakeholder engagement remain relatively unchanged amongst experiences across the literature reviewed.

## Discussion

The objective of the scoping review was to identify and analyse themes within published peer-reviewed literature that pertained to the implementation, utilisation, and improvement of AMR surveillance systems. Overall, the findings of the scoping review demonstrate the diversity in experiences with AMR surveillance systems. Moreover, the review has foregrounded the necessity for a holistic approach in conceptualising AMR surveillance systems with the interwoven nature of the themes presented. The results of the scoping review highlighted the six key themes to be influential in surveillance: capacity, data infrastructure, policy, stakeholder engagement, sustainability, and representativeness. Notably, the scoping review found most literature was published after 2015, coinciding with the first Global Action Plan on AMR [1]. This emphasises a growing recognition of surveillance’s importance as a critical mechanism for public health action.

The prominence of challenges pertaining to data infrastructure highlights a critical need for a renewed overview on how to standardise and quality assure AMR data capture. As the most frequent challenge discussed, there exists considerable overlap with other themes making addressing data infrastructure a multifaceted problem. Interestingly, the paradigm faced by LMICs and higher income countries to improve data infrastructure are vastly dissimilar. For instance, in articles where data infrastructure was discussed as a challenge in LMIC contexts, discussion was focused physical obtainment with auxiliary themes of physical capacity for surveillance and sustainability being influential determinants [19, 29, 43]. Contrasting with higher income countries, challenges faced pertained to broadening the breadth and usefulness of data through further integration of sources [53], technologies [56] and improving timeliness [30]. Indeed, the comparison of economic contexts consolidates the influence health system inequalities impose on endeavours to mitigate AMR [58]. Data for AMR surveillance systems are foundational elements in the process of generating concerted public health action and ensuring adequate feedback is disseminated [59]. As an implication for implementing surveillance systems, considering improvement of contextual health system factors as limit setting steps must be completed before undertaking siloed approaches to improving data infrastructure.

The scoping review reinforces partnership to be a foundational determinant in improving and implementing surveillance systems. Unanimously across all papers reviewed where successes were discussed, stakeholder engagement was pivotal in facilitating action. The result is unsurprising and reflects the largely intricate and multifaceted nature of surveillance systems. While stakeholder engagement is a prominent theme to surveillance systems, the approaches taken in literature are greatly heterogenous. Experiences discussed to improve surveillance through expansion of organisational partnerships [23, 24, 27, 29, 32, 33, 36, 38, 39, 41, 43, 46, 51, 53, 54] undertake a similar overarching approach. However, the nature in which this is implemented is varied and often context specific. Overwhelmingly, literature has reinforced a deficit orientated approach in utilising stakeholder engagement to address gaps. Linking back to the separation of LMICs and higher income countries, it is evident issues with water [21, 23], electricity supply [17, 20–22], foundational training of staff [16, 23, 47], and procurement of microbiological materials [17, 20–22] in LMICs have been addressed through larger organisational and governmental engagement. For higher income countries where the paradigm of conceptual issues are championed [21], introspective approaches to stakeholder engagement have been taken with smaller scale partnerships being created. The implication of this finding for success of surveillance systems is once issues have been identified, stakeholder engagement through partnerships remains an unanimously efficacious approach.

The discourse on stakeholder engagement in animal AMR surveillance yields valuable insights that can strengthen human surveillance efforts. While physical capacity, representativeness, and data infrastructure exhibit commonality in experiences with contextual nuance, stakeholder engagement emerges as a particularly complex aspect. Stakeholder engagement in animal health AMR is particularly intricate due to the sector segmentation across industries and animal types. The complexity is exemplified by the presence of sector-specific AMR data repositories which hinder system unification [14, 26, 60]. For example the Canadian Integrated Program for Antimicrobial Resistance Surveillance (CIPARS) initially faced this disconnect [38] due to political apathy exercised by stakeholders, but was ameliorated through reporting mandates targeting antimicrobial drug distribution [14]. Similar challenges exist in human surveillance which are exemplified with the disconnect between public and private health sectors [45, 52, 53]. Legislative change may be enacted to achieve similar results as in animal health context but may be more difficult to implement. The literature reviewed in the scoping review does not extensively cover experiences with policy to identify leverage points for implementation. As an implication, to improve surveillance systems and foster stakeholder collaboration where barriers may be present, legal foundations for surveillance must be explored.

Ensuring financial sustainability is a fundamental determinant to the long-term viability of AMR surveillance systems. The findings emerging from the review demonstrate LMICs are primarily burdened by the challenges associated with financial sustainability. This finding expected given the economic determinants present within these contexts [43]. Successes detailed in literature to overcome financial barriers have centred around sourcing external funding through trusts and other funds. Indeed, Malania, et al. [18], discusses sourcing funds through the Central Asian and European Surveillance of Antimicrobial Resistance (CAESAR) program, Ashley, et al. [32] details the Wellcome Fund, Gates Foundation, and Fleming fund as potential sources, and Frost, et al. [45] describes the success the Fleming Fund has had with establishing surveillance in Asia and Africa. However, the experiences diverge with more established surveillance systems. There is acknowledgement for external funding’s role in establishment of sustainability, but a notable necessity for internal funding sources to be delineated for long-term surveillance viability [17]. This finding is significant in its implications for the sustainability of surveillance systems. The shift from reliance on external funding towards the development of internal mechanisms for financial stability ensure the long-term viability of surveillance.

Representativeness in surveillance literature emerges as a difficult challenge to manage. The findings of the scoping review highlight the criticality in data and system comprehensiveness to advance overarching surveillance goals. Endeavours to address system representativeness have included capturing meta data to accurately categorise AMR [35, 52, 53, 55] and the expansion of laboratory networks to increase system coverage [16, 26]. However, these goals cannot be achieved without the synergistic improvement of complementary systems related to data infrastructure and physical capacity for surveillance. Overwhelmingly, experiences discussed in the scoping review emphasised a siloed approach without acknowledgement contextual determinants influencing system representativeness. Future efforts should strive for an integrated and collaborative approach towards systematic improvement of system representativeness which leverages the synergy of the associated themes to enhance.

### Gaps in literature

The findings of the scoping review highlight diverse insights into the experiences that contribute to the advancement of surveillance through the acknowledgement of barriers and tailored strategies for success. An interesting point of contention that is obvious upon review of the literature is the absence of surveillance system standards in which denote whether a challenge has properly rectified. With the exception of data infrastructure, through enrolment of in the Global Antimicrobial Surveillance System (GLASS) [21] and external quality assurance schemes for laboratory operation, the absence of a standardised definition of attainment is apparent in all other themes. Understandably, this observation is withholding judgement in whether there is significant benefit in delineating criterion for adequate attainment of physical capacity, policy, stakeholder engagement, sustainability, and representativeness. However, the experiences reviewed in the literature highlight some potential gaps that if addressed, could significantly strengthen surveillance system function.

Antimicrobial resistance surveillance system design is a pertinent component of representativeness that has sparsely been discussed throughout literature. The concept of system design presents an interesting proposition regarding the standardisation of design and comparability between national contexts. Fundamentally, this aligns with the objectives of external programmes such as GLASS which aim to harmonise data, analysis, and monitoring of AMR across international contexts [61, 62]. Without standardisation of system design, data may be presented in a uniform manner, upon initial appraisal, that appears comparable internationally through this endeavour. Despite this apparent uniformity, data may still be subject to sampling and reporting bias which stem from systemic design factors. Only 3 studies provided commentary on surveillance system design but were significantly limited in content for any substantial insights to be derived [22]. Certainly, there are factors such as feasibility and viability that must considered to explain the absence of surveillance system design discussion. Further research addressing this gap may enhance the reliability and effectiveness of the data generated by AMR surveillance systems at an international context, ultimately advancing global efforts.

Introspectively, representativeness at the national level of AMR surveillance is also an unexplored theme within literature. An unexpected finding from the review highlights the necessity for conscious surveillance design which facilitates the nuances of AMR. Representativeness fundamentally refers to the degree in which the collected data accurately reflects the true patterns and trends of AMR within its specified context. Indeed, literature has exhibited desire to rectify representativeness in system design by progressively expanding laboratory networks to facilitate temporal coverage of the system [22, 30]. Albeit the endeavours do not reflect the AMR sampling paradigm which contrasts the infection status of an individual with its clinical significance. In the paradigm, though an individual may carry a.

resistant organism, the process of detection cannot occur without immediate clinical significance of the primary concern [63]. It highlights AMR surveillance to be a sequential system to the to its associated primary disease surveillance. This has significant implications on the monitoring and evaluation of AMR prevalence to the effect that surveillance may conceptually be non-representative without significant re-evaluation of structure. To the knowledge presented in this scoping review, no current human AMR surveillance system considers non-clinical samples. Further research examining the feasibility of non-clinical samples in AMR surveillance would fundamentally subvert the conceptualisation of AMR surveillance and position AMR as the primary concern.

The potential of external tools to enhance AMR surveillance remains underexplored. The scoping review highlights progress in addressing barriers regarding data infrastructure through tools such as WHONet, which aids in the standardisation of AMR data capture with the benefit of providing feedback on data completeness [18, 22, 23, 28, 36, 40, 41, 47, 48]. It is apparent other themes of policy, sustainability, stakeholder engagement, and capacity lack similar tool development due to the diverse complexity presented with the associated contextual factors. Notably, this endeavour remains feasible. Drawing inspiration from the WHO’s International Health Regulations (IHR) and exemplified by the associated Joint External Evaluation Tool (JEE) to help self and external assessment of global health systems [64, 65], a comparable tool could be devised for AMR surveillance. Particularly, standards for satisfactory surveillance function could be delineated with self-assessment tools developed to identify the strengths and weaknesses in current approach across the thematic elements. Further research exploring the possibility the standards and self-assessment tools offers significant potential to enhance surveillance functionality and promote international cross-context comparability.

#### Potential Roadmap for One Health Surveillance Systems

Within AMR surveillance, a One Health approach which aims to integrate, animal, environmental, and human health has been a widely advocated aspiration [66]. Wider AMR literature has hinted at a growing demand for One Health AMR surveillance [67, 68] but the endeavour has yet to be realised [69]. The most influential attribute contributing to One Health surveillances absence is the lack of integration due to a conceptual incongruence throughout sectors [69]. The scoping review and the themes encompassed potentially provide the foundations for a conceptual framework to constructed to facilitate the establishment of a One Health system. Namely, the themes of capacity for surveillance, data infrastructure, policy, stakeholder engagement, sustainability, and representativeness must be addressed to identify relevant contextual factors. In this endeavour, it is of importance for One Health AMR surveillance to be feasible, issues posed to like that to LMICs must first be addressed. This includes ensuring sufficient supplies, training, staffing, and laboratory infrastructure are available. Subsequent themes of data infrastructure, policy, stakeholder engagement, and representativeness will need to be deliberated to identify the most pertinent characteristics that satisfy the system’s objectives. Sustainability may be initially sourced externally like that within literature to facilitate system establishment but will require plans to transition towards internal sustainability like that with established surveillance systems. Pre-emptive planning for One Health AMR surveillance through the experiences delineated within this scoping review has the potential to facilitate the idealisation of such system.

### Strengths and Limitations of the review

There are inherent strengths and limitations of this scoping review. One of the strengths is the breadth of the scoping review and its broad inclusion criteria. This broad approach allowed for the capture of thematic elements dispersed within the larger body of AMR surveillance literature. The inductive approach undertaken allowed for themes to be constructed based on the included literature and would limit the influence of preconceived bias on reported results.

The conducted scoping review has limitations that must be acknowledged. Firstly, the exclusion of grey literature that could encompass official government reports may result in an incomplete representation policy and sustainability discussion. Additionally, the absence of grey literature may have limited the inclusion of other relevant themes that are critical to AMR surveillance function. Furthermore, the absence of a formal risk of bias assessment for the included articles may potentially influence the interpretability of the results. Further research including risk of bias assessment may limit the potential for selection, reporting, and measurement bias from being introduced and facilitate greater qualitative synthesis of themes. The implications of this limitation may prohibit the accuracy in the themes identified and facilitate inaccuracy in categorisation of studies into constructed themes.

## Conclusion

The scoping review has demonstrated an immense diversity of experiences in implementing, utilising, and improving AMR surveillance systems across all contexts. The emergence of six key themes of capacity, data infrastructure, policy, stakeholder engagement, and representativeness foreground the necessity for a holistic in developing AMR surveillance systems. Challenges with data infrastructure and financial sustainability, particularly in LMIC contexts, require immediate action to ensure optimal function and long-term viability of surveillance efforts. Stakeholder engagement emerges as a key determinant in overcoming challenges and reflects the deeper, interconnected intricacies of surveillance that has been unanimously successful in addressing potential barriers. Whilst complex, stakeholder engagement is pivotal and necessitates context-specific strategies for success. Gaps to address include system standards and design, alongside the exploration of external tools offer promising avenues for enhancing AMR surveillance functionality and inter-context comparability. The environment of One Health approach to AMR surveillance by the scoping review is certainly feasible given the key themes identified within the scoping review are pre-emptively planned and addressed adequately. The themes in the scoping review facilitate the pursuit of refined and strengthened AMR surveillance to be possible to ultimately inform concerted global health action.

The experiences in implementing, utilising, and improving AMR surveillance systems have been identified to be divergent across contexts. The scoping review has elucidated the common themes of capacity, data infrastructure, policy, stakeholder engagement, sustainability, and representativeness as central to the discussion of AMR surveillance systems. The implications of the review’s findings suggest stakeholder engagement is fundamental to improving all facets of AMR surveillance. From the findings, it is critical this remains central to efforts involved in surveillance. However, there remains to be a gap within surveillance representativeness that requires further attention. For this gap to be addressed, further work must be completed to conceptualise a methodology to standardise representativeness. The result of this concentrated effort will further strengthen AMR surveillance endeavours.

### Electronic supplementary material

Below is the link to the electronic supplementary material.

Supplementary Material 1

Supplementary Material 2

Supplementary Material 3

## Data Availability

Data supporting the conclusions of the study is found within the supplementary files. All other data is presented within the results section.

## List of abbreviations

AMR: Antimicrobial Resistance
GAP: Global Action Plan
GLASS: Global Antimicrobial and Resistance Use Surveillance System
IHR: International Health Regulations
JEE: Joint External Evaluation
LMIC: Low- and Middle-income countries
NAP: National Action Plan
PRISMA-ScR: Preferred Reporting Items for Systematic Reviews and Meta-Analyses for Scoping Reviews
WHO: World Health Organization
