# Supplementary material for: Strengthening antimicrobial resistance surveillance systems: a scoping review
Source: BMC Infect Dis. 2023 Sep 11;23:593. doi: 10.1186/s12879-023-08585-2 (PMC10496311; doi:10.1186/s12879-023-08585-2)
Supplement: Supplementary file 1 — Supplementary Material 1 [file 12879_2023_8585_MOESM1_ESM.pdf]

Child codes

Legend: Supplementary File 1 Child Codes

The following supplementary file displays the raw paragraphs from each article included in the scoping review. Codes were applied basic on ideological themes of the paragraph that captured the essence of the discussion points made in the paragraph.
Major areas of emphasis were experiences with surveillance and not raw numbers or data quoted.

Acharya et al. 2021

A total of 1584 records from five reporting sites were screened for consistency in the specimen–pathogen combination as per the GLASS criteria, with 1147 (72.4%) isolates assessed for urine, 403 (25.4%) for blood, 27 (1.7%) for stool, and 4 (0.3%) for genital swabs. Overall, 1571 (99.1%) records from all sites were found to be 100% consistent for blood, 96–99% for urine, 88.9–90.5% for stool, and 75% for genital swabs (Figure 1). In the figure below, the x-axis represents the five "reporting" AMR surveillance sites, and the y-axis denotes percentage consistency of specimen–pathogen combination for the four specimens indicated with different colored bars.

- Cases reported
- Record screening
- Screening criteria

A total of 1584 records were assessed for the pathogen–antibacterial combination, with a 66% overall consistency. The consistency in reporting the pathogen and antibacterial sensitivity test according to GLASS criteria varied from 52% to 88% across the different sites, and the numbers varied among the organisms, too. The consistency was high for Salmonella spp. (94–100%), but the averages ranged from 54% to 83% for other bacteria such as Staphylococcus aureus (83%; range: 0–100%), Shigella spp. (71%; range: 0–86%), Escherichia coli (63%; range: 49–91%), Acinetobacter spp. (55%; range: 0–100%), and Klebsiella pneumoniae (54%; range: 48–70%). No consistency was found at any site for Streptococcus pneumoniae and Neisseria gonorrhoeae

- Screening criteria
- Screening consistency

When the AMR surveillance records at the sites were compared to records sent to the NPHL, there was completeness overall, except for a variable origin (hospital or community), which were missing data at two of the sites (sites B and C) and incomplete at two more (94% at Site A and 53% at Site D). For Site B, the antibiotic susceptibility, specimen–pathogen combination, and pathogen–antibacterial combination data were all incomplete (99.7%, 96.9%, and 97.9%, respectively)

- Surveillance records
- Reference laboratory
- Missing data
- Data completeness

Upon site visits, the specimen and isolate data were found to be 100% consistent at sites B and C, whereas the consistency at the remaining three sites varied from 84.8% to 99.1% for specimens and 84.8% to 94.5% for isolates. Only Site C had consistent data on the antibiotic susceptibility pattern; at the remaining four sites, the consistency varied from 80% to 94.5%. The specimen–pathogen combination and the pathogen–antibacterial combination consistency varied among the different sites, with ranges of 46.2–89.4% and 47.1–89.4%, respectively

- Consistency in isolate data
- Data from sites

The completeness of records from the "reporting" sites received at the NPHL varied from 88% to 100%, whereas the consistency varied from 77% to 92%. Data from only two sites were received by the 15th working day of the following month, whereas receipt was delayed for the other three by an average of 175 days (range: 8–269)

- Data and record completeness
- Data timeliness

Barriers in Reporting AMR Surveillance Data The structured questionnaire completed by the microbiology staff at the nine AMR surveillance sites indicated adequate staffing in the microbiology department. A dedicated room for data entry was available at only 50% of the "non-reporting" sites (Sites 1 and 2) whereas it was available at 80% of the "reporting" sites. The data entry area was in a shared space at Sites 4 and B. A computer for data entry with an adequate Internet connection speed was available at only two "non-reporting" sites (50%) as compared to all five (100%) "reporting" sites.

- Barriers (challenges) by staff
- Data entry limited
- Inconsistent reporting
- Internet connection/speed

None of the sites had dedicated personnel available for data entry, which was being temporarily performed by laboratory personnel with a qualification ranging from certificate in Medical Laboratory Technology (CMLT), Bachelor of Science in Medical Laboratory Technology (BSc.MLT) to MSc. Microbiology.

- Dedicated personnel for data entry
- Laboratory personnel qualifications
- Education varied

Although most microbiology staff at all sites had received the AMR surveillance training, the data entry person at one of the "non-reporting" sites had not received the data analysis and WHONET training.

- Microbiology staff training
- Data entry person not trained
- WHONET/Data training

All of the sites had a Terms of Reference based on the 2013 AMR guidelines (published by the NPHL) and a verbal agreement with the NPHL to share the AMR data regularly. Three of the four "non-reporting" sites did not have any institutional restrictions on data sharing

- Agreements
- Data sharing restrictions

Altorf-van der Kuil et al. 2017

Sampling habits were until recently not under financial constraints. However, recent policies aimed at reducing healthcare costs that require patients to pay the first EUR 385 themselves, may cause patients to refuse diagnostics being done. To which extent these policies lead to bias in the surveillance data remains to be studied

- Sampling limiting due to finances
- Bias in surveillance data

At GP practices and in nursing homes, sampling policy is more restrictive: microbiological samples are usually only collected after one or more empiric treatment failures or recurrent infection.

- Sampling restrictive after one treatment failed

The national AMR surveillance system provides opportunities for in-depth scientific research that generates additional knowledge on AMR. Examples of research based on Dutch national surveillance data are studies on the impact the EUCAST breakpoint implementation in Dutch MMLs had on resistance levels in surveillance data [17,26], a review on the adequacy of the urinary tract infection treatment guideline in hospitalised patients [27], a trend analysis for AMR in hospitals where SDD/SOD is applied vs hospitals where this is not the case [28,29], trends in the proportion of E. coliand K. pneumoniae with an ESBL-producing gene [30], and detection and epidemiology of carbapenemase-producing Enterobacteriaceae in the Netherlands [31]

- Data break point on surveillance
- Trends with species
- Epidemiology of species

As an additional effect of national surveillance, participationof the MMLs in the national AMR surveillance network has contributed to sharingof knowledge and quality improvement. Triggered by the feedback on the monthlyfile, many MMLs perform more confirmatory tests for potential HRMOs and exceptionalphenotypes such as carbapenem resistance in Enterobacteriaceae or vancomycinnon-susceptibility in methicillin-resistant S. aureus (MRSA).Furthermore, quality control by the national surveillance team has revealedmethodological issues with regard to AST. For example, in data fromlaboratories using the VITEK2 test panel P608, it was not possible todifferentiate between rifampicin-susceptible and -intermediate S.aureus isolates according to EUCAST breakpoints because the lowestconcentration in the test panel was at the R breakpoint of ≤0.5. In the new test panel P633, theconcentration range is adapted.

- Participation MML has contributed to knowledge and quality
- Difficulty to differentiate between susceptibility

Finally, the national surveillance network provides aplatform for further harmonisation of AST methods in participating MMLs, whichbenefits the comparability of local data with nationwide data. One of theinitiatives that were supported by the national surveillance network was thedevelopment of a guideline on a uniform test panel for disk diffusion inmicroorganisms that cannot be tested with automated systems. This guideline isintended to harmonise the test panels between laboratories and consequentlyprovide information on resistance for both surveillance and clinical purposes

- Further harmonisation of efforts
- Comparability of results/uniform test

To enhance quality and timeliness of data delivery to thenational AMR surveillance system, the NVMM, RIVM and other stakeholders areworking together to develop a new electronic messaging standard. This comprisesa common data model, a new semantic standard [32] that is a subset of theexisting international coding schemes SNOMED (www.ihtsdo.org/snomed-ct) andLOINC (www.loinc.org) as well as different HL7-based (www.hl7.org) datatransfer messages. A number of MMLs, together with the RIVM, different LIMSvendors and other stakeholders, have started a pilot project that uses the newstandards to generate and send data extractions to the database of the nationalsurveillance system

- NIVM, RIVM and stakeholders developing standard

Data collection in the national AMR surveillance system iscurrently limited to phenotypic susceptibility tests and some epidemiologicalinformation. To amplify the impact of the surveillance system on public health,healthcare and scientific research, opportunities are being explored to mergedata from the national AMR surveillance system with existing data on type ofinfection, antibiotic use, co-morbidity and mortality at patient level and/orgenotypic data. This will require standardised, encrypted patient and sampleidentifiers in each system, but once established, it will allow insight intothe risk factors, spread and burden of AMR.

- Data collection limited to phenotypic
- Merger of data
- Identification of risk factors

Another envisioned development is the design of an algorithmfor the detection of (multi-institutional) outbreaks of HRMOs. In theNetherlands, hospitals 8 www.eurosurveillance.org usually have a well-functioninginfection prevention policy in which detection of local outbreaks is incorporated.Nevertheless, individual laboratories do not have access to data fromneighbouring laboratories and analysis of data from the national AMR surveillancesystem can serve as a safety net to detect local or multi-institutionaloutbreaks. We used SaTScan software (www.SaTScan.org) to develop an algorithmto detect clusters of resistant microorganisms in space and time, but many ofthese clusters were found not to be clonal upon validation (data not shown).Currently, a new approach is being developed to detect clusters of resistantmicroorganisms, possibly in combination with other datasets

- Algorithm for detection
- Data not accessible by neighbours

A remaining challenge is to develop a method to adequatelydistinguish between healthcare-associated and community-acquired infections.Currently, it is not possible to identify which patients are transferred fromone hospital to another because the national AMR surveillance system does notuse a countrywide unique patient identifier. This may lead to healthcare-associatedinfections being misclassified as community acquired. Because the distinctionbetween these types of infection is increasingly important in the context ofantibiotic stewardship, it is desirable to have insight into healthcaretransfers by using countrywide unique patient identifiers, which is currentlynot available for our system.

- Origin of infection challenge
- Does not use unique patient identifier

Argimon et al. 2020

Although considerable in volume, the data lacked completeness and consistency, with the main issues being related to the origin of the isolates (hospital or community setting). The data received at the NPHL also did not follow the GLASS criteria for the specimen–pathogen and pathogen–antibacterial combinations and often were submitted with delay. We looked as well at infrastructure availability at the surveillance sites, and among the four "non-reporting" sites, most lacked dedicated personnel for data entry and had not met some specific requirements, such as having a computer. Other researchers have stressed the need for infrastructure and human resources for reliable data in surveillance

- Data completeness and cosnsitency
- GLASS criteria for specimens
- Physical infrastructure in computers
- Infrastructure availability
- Lack of personnel

Generation of quality AMR data requires good infrastructure; otherwise, it will be faced with challenges, particularly in resource-limited laboratory settings

- Quality AMR data needs infrastructure
- Resource limited laboratories

In Nepal, Malla et al. [14] had, by 2014, already highlighted the challenges to AMR surveillance implementation, giving high importance to data collection, compilation, and storage. Many others later noted that the availability of an electronic data collection system with data back-up, as opposed to manual paperwork collection, would result in a smoother

- Challenges to data collection, compilation, storage
- Availability of electronic data collection
- Smoother data pipeline with infrastructure

WHONET software integration for surveillance proved challenging at the data entry site, and they opted for an individualized solution for data entry. Ibrahim et al. also found data transmission challenges in Ethiopia's reporting to the GLASS, although they could improve it by on-site mentorship and frequent site visits with weekly calls

- WHONet software integration challenging
- Opted for different entry solution

In our study, the AMR surveillance site personnel had received training in how to enter data into WHONET, but two of the four "non-reporting" sites did not use the system because no computer was available.

- Surveillance site personnel training
- No computers to use

At most of the surveillance sites, heads of the institution had not signed documents, such as an official memorandum of understanding or a written "Terms of Reference," recording their commitment to report to the NPHL for the GLASS.

- Institution heads not signing
- Troubled committment

National networks of laboratory-based surveillance are a keypillar within the Global Action Plan to combat AMR, with the referencelaboratory playing a central role in the dissemination of surveillance data tolocal, national and international stakeholders. The Philippines ARSPsurveillance data have shown increasing resistance trends for key bug–drugcombinations (Figs. 1b and 2a). In this study, the analysis of the dynamics ofRPs showed the concomitant expansion of specific resistance phenotypes (Fig.2b). By complementing laboratory data with WGS and linking the operationalunits of laboratory and genomic surveillance, we revealed a diversity ofgenetic lineages, AMR mechanisms and vehicles underlying the expansion ofcarbapenem resistance phenotypes (Figs. 1a and 3). The combination of thesethree components vastly improved our understanding of the drivers of carbapenemresistance in the Philippines at different geographical scales, from aplasmid-driven local outbreak of K. pneumoniae ST340 (Fig. 4), to the expansionof a K. pneumoniae ST147 clone carrying NDM-1 in an IncA/C2 plasmid without anyclose match in international genomes (Fig. 5), to the independent introductionsof international epidemic clone E. coli ST410 that can acquire plasmids oflocal circulation.

- Complementing laboratory data with WGS
- Improved understanding

During the course of our project it became apparent thatthere were inconsistencies in the panels of antibiotics tested by the sentinelsites (missing data or different antibiotics tested), which led to theexclusion of samples from the analysis of the dynamics of RPs (Fig. 2). Theseinconsistencies would also hamper cluster detection based on RPs, or the earlydetection of novel emerging RPs that could act as indicators of novel high-riskclones. The joint discussions of these observations by the two collaboratingteams led to an effort to reinforce the standardization of antibiotic testingacross the ARSP sentinel sites, which was coordinated by the ARSRL.

- Inconsistencies in panels
- Exclusion of samples
- Hinder detection
- ARSRL coordination

Even with complete and comprehensive susceptibility testing,RPs can only provide a coarse view of the spread of AMR, and sometimes lead toclusters of disparate genetic relatedness (Fig. 3 and Supplementary Fig. 2).Integration of WGS into laboratory-based AMR surveillance can substantially improvethe detection of high-risk clones by providing a high-resolution picture ofgenetic lineages (strains), AMR mechanisms (genes/mutations) and vehicles(MGEs, Fig. 1a). We identified high-risk clones within known internationalepidemic clones at the local, national and international scales by linkingclonal relatedness, geographic clustering, epidemiological data, and gene andplasmid content with interactive web tools 27.

- Integration of WGS improves representation
- Substantial improvement with integration
- Identification of risk clones by linking data

Ashley et al. 2018b

Pharmaceutical companies generate a large volume of highquality bacterial susceptibility data before and after new agents are marketed, in order to fulfil regulatory requirements. These data are largely undisclosed, but some companies are nowproviding aggregated data, including Pfizer, which has developed ATLAS (Antimicrobial Testing Leadership and Surveillance), a searchable database on resistance to Pfizer anti-infective agents.5 The SENTRY Antimicrobial Surveillance Program is a notable commercial surveillance programme run by JMI Laboratories since 1997 that collects data from .200 sites worldwide,6 largely from the USA and Europe. Findings based on aggregated data of specific species or genera are published, but the dataset is proprietary and not available for wider use. GSK began the Survey of Antibiotic Resistance (SOAR) study in 2002. This focuses on the effectiveness of antibiotics in the treatment of community-acquired respiratory tract infections. 7 SOAR concentrates in particular on countries and regions for which little other susceptibility data are available; the findings are regularly reported in the published literature

- Data that is undisclosed
- Companies providing aggregated data
- SENTRY - JMI laboratories (global surveillance)

Pharma data have several strengths. Isolates are obtained from a global distribution. Organism identification and susceptibility testing procedures are strictly standardized and quality controlled according to international standards. Isolates are transported and retested in a central accredited laboratory, ensuring reliability and reproducibility of results. However, there are several notable disadvantages. There are often no metadata (clinical presentation and outcomes or demographic information) associatedwith the isolates. Organismsampling fulfils the requirements of the particular pharma project rather than being representative or generalizable to the local population and centralized testing means that quality-controlled test data are not available to guide individual patient care; also, local laboratories do not benefit from improved quality management as a result of participation. Locations perceived to represent smallmarkets are typically under-represented. Furthermore, there is no information on denominator data and so the resultsmay be biased and may not reflect the true burden of resistance in the area tested

- Data is standardised and reproducible
- Quality controlled
- Often no meta data
- Local laboratories do not benefit from participation

Research data have several potential strengths. Laboratories may be subject to good laboratory practice when the information generated is performed to specific standards, supported by methods that are rigorously evaluated through robust quality-assurance and quality-control programmes. The methods used to generate bacterial susceptibility data are notoriously error prone and data generated outside of quality-controlled laboratoriesmay be of suboptimal quality;10,11 the inclusion of quality-assured research data in national and global databases could increase the proportion of accurate data points. Patient information may also be collected on clinical presentation, duration of hospital stay, antibiotic treatment, complications and outcome. Furthermore, understanding the trajectory for resistance often requires evaluation of susceptibility data over long timescales and newer national surveillance programmes may not yet have sufficient retrospective information tomake themost of newdata.

- Research data has rigorous quality assurance
- Quality controlled
- AST error prone outside quality laboratory
- Increase proportion of accurate data points

Research-generated data also have several potential limitations. Data may be biased, including ascertainment and sampling biases.12 For example, the study designmay target patient subsets that do not reflect the wider population with infectious diseases, such as sampling of patients within cohorts that have better access to care or patients with themost severe infection syndromes. Since a proportion of bloodstream infections will be hospital acquired, studies of severe invasive diseasemay inflate rates of resistance and may not capture milder forms of communityassociated infection in patients treated as outpatients, whichmay be caused by organisms with lower AMR. Research data may also include duplicate samples. Six main types of potential bias that may influence the validity or interpretation of surveillance data have been identified and these provide a framework for reviewing the use of research data in AMR surveillance (use of inadequate or inappropriate denominator data; case definitions; case ascertainment; sampling bias; failure to deal with multiple occurrences; and biases related to laboratory practice and procedures).

- Data may be biased
- Duplicate samples
- Interpretation of results

A source of susceptibility data thatmay remain unseen by national surveillance systems, particularly in many LMIC settings, are laboratories in private hospitals that generate data for patient care.14–16 The quality of data generated by private laboratories varies considerably, but those that are accredited and performquality- assured services produce data of similar or better quality than that produced by public laboratories. In India, almost all medical laboratories accredited by the National Accreditation Board for Testing and Calibration Laboratories (NABL) are in the private sector and, in South Africa, .80% of South African National Accreditation System (SANAS)-accredited medical laboratories are in the private sector.15 This has led to calls to utilize these data and the inclusion of such data by initiatives such as ResistanceMap.17 This displays AMR data on 12 bacterial species isolated in 49 countries, collected between 1999 to 2015 (depending on the country), together with antibiotic consumption data from 75 countries between 2000 and 2014. The primary sources of data are public and private laboratory networks that routinely collect susceptibility results, but data from India come exclusively fromthe private sector.

- Integration of private data sources
- Quality of data varies in private laboratories

Despite the obvious utility of placing AMR surveillance data generated by academia, pharma or private laboratories into the public domain, very little of these data generated in LMICs is utilized by organizations involved in regional or global surveillance. There are several barriers that prevent this fromhappening. Data are held in numerous silos with highly restricted access. Academics generate data that usually remain private until published in peer-reviewed journals; individual patient-level data may not be released or may be delayed by several years fromthe point of collection because of the time taken to analyse, write and publish. Pharma companies have to jump through several legal hoops before they release their data into the public domain. Even if researchers and pharma companies are keen to deposit data towards global analyses, data aggregation is hampered by a lack of harmonization in data collection, a lack of tools that allow easy data deposition and the lack of a framework that prevents publication of their data by unscrupulous competitors. Furthermore, GLASS collects and reports data on resistance rates aggregated at national level byministries of health and cannot currently accept information generated by research activities or pharma. In general, national programmes take ownership of in-country surveillance activities and agreement may not be reached for direct data deposition to WHO GLASS by non-governmental groups. Furthermore, AMR surveillance data can represent potentially sensitive data, particularly when these describe high rates of resistance or the emergence of a novel resistancemechanismto a key antibiotic.

- Data is siloed and restricted
- Remain private until published
- Time delay
- Aggregation of data is not standardised
- GLASS cannot accept data from pharma or research

Bottom-up' research and pharma activity that generates AMR data is not public health surveillance in the strict sense. Furthermore, the majority of researchers and pharma-employed scientists would be quick to highlight that public health surveillance is neither their responsibility nor area of interest. Agreeing on the principle that researchers and pharma companies couldmake a major contribution to global surveillance should be aligned with the recognition that this is not their primary purpose and will be associated with a financial cost. Debate is required about incentives to support the additional workload associated with sharing data with national programmes or other repositories and who should coordinate this. This discussion could draw on experience gained fromacademic incentives during the development of the WorldWide Antimalarial Resistance Network (WWARN) platform. 18 Any investment should not detract fromfunding that provides improved data sources for patient care, surveillance and prevention of AMR.

- Agreeing on principles for contribution
- Financial costs
- Incentives to improve sharing of data

The flow of research-generated data into global initiatives could be facilitated by funders, who could develop guidelines on sharing of specific datasets, a procedure that could become an integral component of a successful funding award. This is already the case for some forms of data, examples being the submission of all sequence data generated by the Wellcome Sanger Institute to public databases and funding by Wellcome being linked to an open access publication policy. Such changeswould require a clear plan for formatting and destination of data deposition. Journals and publishers could also develop guidelines on data deposition for publications on drug-resistant infection and could make this a necessary part of submission. Data released into public databases by researchers would need to be protected by data access committees or through othermechanisms, but this is not insurmountable because solutions are already in place for numerous types of data.

- Global initiatives can be facilitated by funding
- Wellcome Sanger Institute already has a initiative for publication

Ashley et al. 2018a

In terms of the pathogens under surveillance, 45 networkswere for AMR in bacteria or fungi (Table 1), 18 in malaria, 2 in TB, 6 in HIVand 1 for influenza (Table 2). The median (range) duration of the networks was6 years (1–70). In the case of the discontinued malaria networks, inability tosecure sustainable funding was an important reason for their collapse.4Coverage of LMICs by the networks varied greatly. The median (range) number ofLMICs included in the AMR surveillance networks for which the information wasavailable was 8 (1–67). The WHO Global Influenza Surveillance and ResponseSystem (WHO GISRS) was the longest running network, established in 1947, andincluded the greatest number of LMICs (67), although antiviral resistance wasnot under surveillance at the outset.

- Coverage of LMIc varied
- Sustainable funding needed
- Collapse due to funding

The networks were a heterogeneous group with differentapproaches to surveillance reflecting different objectives. The greatestdiversity was found in the antibacterial surveillance group. Most globalnetworks initiated and sponsored by pharmaceutical companies had the objectiveof evaluating susceptibility to specific drugs (registered drugs or newcompounds). A variety of bacterial or fungal pathogens were collected by thepharma networks including community- and hospital-acquired isolates from bothsterile and non-sterile sites. Academic networks tended to focus AMR surveillancearound a specific clinical question, e.g. one project of the Asian Network forSurveillance of Resistant Pathogens (ANSORP) evaluated susceptibility ofESBL-producing isolates collected in the region to different antimicrobials(Tables 1 and 2). Other academic networks such as the WorldWide AntimalarialResistance Network (WWARN) part of the newly established Infectious DiseasesData Observatory (IDDO) and International Epidemiologic Databases to EvaluateAIDS (IeDEA) have led analyses of individual patient data collected by otherresearch groups.

- Heterogenous approaches
- Global networks are sponsored
- Academic networks - clinical
- Pharmaceutical - susceptibility

There is one supranational European network for surveillanceof food- and waterborne diseases and zoonoses that collects data onantimicrobial susceptibility in humans, animals and food. Larger networks thatmonitor foodborne infections [WHO Global Foodborne Infections network (GFN) andPulseNet International], including animal and environmental isolates, do notreport AMR data although GFN does support an external quality assurance (EQA)programme for participating laboratories, which includes antimicrobialsusceptibility testing (AST). No other supranational networks for AMRsurveillance in animals were identified.

- Supranational European network
- Food, animals, and human
- No other identified

The networks had different approaches to quality management(Table 4). The pharma-led networks typically did not involve LMIC laboratoriesin EQA programs but sent all isolates to a central laboratory for confirmatorytesting. The global surveillance programs for AMR in TB, HIV, influenza, andgonorrhoea all had proficiency testing programs delivered via supranationalnetworks of reference laboratories. Among the networks for AMR surveillance inbacteria, the Latin-American network, Red Latin-Americana de Vigilancia de laResistencia a los Antimicrobianos (ReLAVRA) has been running an EQA scheme(LA-EQAS) since 2000 and provides proficiency testing services at no cost toparticipating laboratories. The Central Asian and Eastern European Surveillanceof Antimicrobial Resistance (CAESAR), the non-EU European network, has used theUK National External Quality Assessment Service (UKNEQAS) for EQA.WHO-sponsored EQA efforts for AST included the discontinued WHO EQAS AST(1998-2001)5 and the WHO-AFRO/NICD-SA EQAP for countries within the WHO-AFROregion.6 Currently, GLASS recommends national reference laboratories takeresponsibility for quality management.

- Proficiency testing programs via Supranational networks
- AMR networks - Latin American, non-European, and Central and East Asian networks have EQA schemes to laboratories

Table 5. Impacts and challenges of the AMR surveillance networks in LMICs with examples

Impacts	Challenges
• Led to changes in treatment policy (malaria networks)<br>• Improved laboratory capacity by establishing networks of reference laboratories and quality management systems (ARMed, WHO/IUATLD, GASP, ReLAVRA, CAESAR)<br>• Standardization of surveillance methodologies and data analysis (WHO Global Malaria Programme, ReLAVRA, WHO/IUATLD, HIVResNet, WHONET, WWARN)<br>• Reduction in healthcare-associated infections in countries (INICC)[21,22]<br>• Exchange of information, training and knowledge between countries (WHO, ReLAVRA, WWARN, netSPEAR)<br>• Data sharing with secondary benefits to inform treatment guidelines (WWARN, IeDEA)<br>• Created global repositories of bacterial isolates; these can be used to screen new drugs (SENTRY, ANSORP)<br>• Discovery of new resistance mechanisms (The Alexander Project)	• Low coverage, particularly in sub-Saharan Africa and India (GASP, GISRS)<br>• Lack of representativeness of data, e.g. due to selective sampling (HIV, GASP, some CAESAR sites)<br>• Difficulties of implementing routine blood culture/diagnostic microbiology in clinical practice (CAESAR)<br>• Difficulties in implementing complex surveillance methodologies, e.g. optimal *in vivo* methods for surveillance for artemisinin resistance in malaria, second-line drug susceptibility testing for TB<br>• Lack of engagement by some partners (netSPEAR)<br>• Reporting delays<br>• Sustainability due to underfunding with consequent understaffing; surveillance has generally not been given high priority by external donors (EANMAT, netSPEAR)

- Low coverage
- Lack of data representativeness
- Difficulty in routine implementation
- Difficulty in method integration
- Sustainability
- Lack of engagement
- Reporting delays

The biggest challenges faced by the global networks havebeen achieving high coverage across LMICs and complying with the recommendedfrequency of reporting. The Global Project on Anti-Tuberculosis Drug ResistanceSurveillance has collected resistance data from 155/194 member states since itsinception in 1994. For 72 countries without routine drug susceptibility testingof cases, these data come from surveys, which are ideally performed every 5 years.The biggest gaps in surveillance in the most recent report were over West andcentral Africa. At an individual level, it was estimated that 33% of new TBcases and 60% of cases treated previously underwent rifampicin susceptibilitytesting in 2016.7 Only one-third of 106 malaria endemic countries were incompliance with the recommended targets for antimalarial drug efficacysurveillance (monitoring at three-yearly intervals) when last reported,although the Global Malaria Programme has recently updated its website withaggregate data from more studies.8,9 The Gonococcal Antimicrobial SurveillanceProgramme (GASP) has had no regional focal point in Africa since 2012. The WHO2014 Global Report on Surveillance obtained data on antimicrobial susceptibilityin N. gonorrhoeae from only 42/194 (22%) member states and noted that coveragewas poorest from presumed high-burden countries. WHO GISRS reported resistanceto the neuraminidase inhibitors of influenza viruses in 2016.

- Challenge in coverage
- Complying with frequency of reporting
- No routine susceptibility testing of cases
- Coverage poor in high burden countries

In a detailed account of the experience of setting up the Network forSurveillance of Pneumococcal Disease in the East Africa Region (netSPEAR), anEast African network funded by the GAVI Alliance, in which routine surveillancefor pneumococcal disease in public hospitals was strengthened, key challengesnoted were difficulty in engaging the government of one of the participatingcountries in the network, poor performance of some sites despite training andproblems with attracting funding. The importance of national and institutionalownership of surveillance activity and of framing it as part of routineactivity rather than extra work was stressed. The benefits of collaborationbetween policymakers, academics and service providers were highlighted, asentiment echoed by the experience of the malaria regional networks, whichre-energized surveillance and also played a role in advocacy for policy change,acting as a bridge between research groups and national control programmes.Individual patient data meta-analyses coordinated by WWARN have led to policyrecommendations to change antimalarial drug dosing. Another impact of theacademic malaria drug efficacy surveillance networks has been the establishmentof successful North-South scientific partnerships. There are a few exampleswhere the scientific leadership now comes from the South, e.g. PlasmodiumDiversity Network Africa, a molecular surveillance network.

- netSPEAR strengthened
- Difficulty in engaging goivernment
- Difficulty in sourcing funding
- Collaboration as a bridge between sectors

There are also examples of training and data sharing/open access agreements having been developed that are contextualized and locally acceptable

Wellcome has begun to address access to untapped sources of global surveillance data held by pharma through a recently funded project conducted by the Open Data Institute. This has created a mechanism to bring together leaders from public health and the pharmaceutical industry, who are collaborating to explore how value could be added by the reuse of available data. An evaluation of the mechanisms and barriers to making this open access has been completed and detailed in a post-project report.21 One proposal is to create a public–private partnership to improve local laboratory capacity. Another is to suggest that relevant metadata and denominator data are also collected, fulfilling the objectives of the pharma project whilst also providing information about local AMR prevalence that, while not informing individual patient care, could inform empirical prescribing protocols.

- Wellcome data institute funding
- Public private partnership to improve local laboratory capacity
- Public private partnership to improve local laboratory capacity

Ministries of health could collaborate with research institutions where this is not already the case or a public–private partnership could be forged so that data generated by research, pharma or private laboratories can be submitted to GLASS as national data. There are examples of research units in Asia and Africa that have already developed close and sustainable working relationships with the relevant ministry of health, which uses the information provided to shape national prescribing policies. In this way, countries can be empowered to utilize data generated in their own territory, with local researchers undertaking in-depth analyses using a range of sources, thereby encouraging comparisons of incidence and prevalence rates of drug-resistant infection between different areas in the country in order to monitor the disease burden and the impact of action plans in each area. This also represents an important training opportunity for government staff, who can develop the technical capability to analyse data that are ultimately generated through country-led capacity building

- Public and private partnerships for data generation to be submitted to GLASS as national data
- Asia and africa have examples
- Training opportunity for staff for technical ability

Having argued that the development of newinitiatives that effectively replicateWHO GLASS and that fragment data are generally to be discouraged, there are some circumstances when additional networks add vital new information. A notable example is the Institute for Health Metrics and Evaluation, which has recently been funded by a joint award from Wellcome, the UK Fleming Fund and the Bill and Melinda Gates Foundation to gather, map and analyse disease and death caused by drug-resistant infections. This will be used to quantify the global burden of disease (GBD) due to drug-resistant infections compared with other diseases and causes of death, and so inform policy- and decisionmaking. Estimating the GBD due to AMR faces numerous challenges, including difficulties in linking surveillance datawith clinical or outcome data and causal attribution, and cannot be regarded as a routine surveillance activity at present.

- Wellcome, UK fleming fund and Gates foundation for funding
- Inform decision making
- GBD difficulties in linking surveillance data with clinical outcome and causal attribution

Investment is needed to promote innovation in AMR surveillance. For example, harnessing emerging technologies relating to 'Big Data' and artificial intelligence could lead tomore effectivemechanisms of AMR data capture, sharing and analysis tools. An innovative system to support automatic data harmonization between different laboratories and institutions could achieve numerous objectives, including: an inbuilt system to standardize data analysis and quality tests for data from multiple sources; capture of patient outcome data to underpin calculations of GBD; and rapid relay of information to treating clinicians, e.g. via electronic decision support algorithms. Data could be automatically linked to national agencies and international data repositories. Innovation in data capture would benefit from early involvement of experts in the social sciences so that the behaviour change required to support buy-in is an integral part of planning and development. Mapping of data sources may also require consideration of the regulatory environment in some settings. Innovation needs to be linked to effective translation, scale-up and integration, and assessed in terms of impact on policy.22 Any alternative system

- Investment into AMR surveillance
- Innovation for new methods for data capture
- Linking of systems
- Engaging experts

Burns et al. 2018

The time and technical challenges to retrieve data from the AHC laboratory information system (LIMS) were identified as barriers that had precluded previous analysis and reporting. These barriers might also exist in other provinces. CAHSN provided an easier to use alternative to the previous LIMS system, and CAHSN could be quickly and reliably queried to retrieve real-time data (24). In addition, the CAHSN system allowed retrieval of anonymized data, eliminating the need for post-retrieval anonymization, thereby further reducing data security concerns

- Time challenges to retrieve data from LIMS
- Technical challenges to retrieve data
- CAHSN system allowed for faster and more secure retrieval

Robust surveillance systems include multiple surveillance components. Examining passively acquired AHC data with actively and passively acquired CIPARS data provided a comparatively low-cost method to use existing data to increase AMR surveillance in BC. When interpreting the data obtained from the CIPARS and AHC components, it is important to note differences between CIPARS and AHC data, which include:
i) CIPARS implements an active, random sampling strategy, while AHC relies on passive sample procurement; ii) CIPARS samples are obtained from a larger portion of the food production continuum (live animals through abattoir to retail meat);
iii) some CIPARS results are national and not specific to BC;
and iv) AHC isolates are more likely than CIPARS isolates to originate from individuals that are sick or unthrifty, and might be more likely to have received antimicrobial treatment.

- Low cost method to use existing data to increase AMR surveillance
- CIPARS - random sampling strategy
- AHC relies on passive sample procurement
- CIPARS national and not specific to BC
- AHC isolates more likely to originate from individuals that are sick

Preliminary findings from examining the AHC and CIPARS components together by food chain value, provide new information, which might be valuable for future surveillance activities. For example, during the survey of experts, interviewees expressed a common concern that using data from sick animals might lead to erroneously high estimates of AMR. Given the limitation of small sample sizes for some sample types, general similarities between AHC and CIPARS AMR for commodity chains indicate that this assumption warrants further examination. In humans, most AMR samples come from diagnostic samples. As another example, AHC data added evidence to support CIPARS data showing very low resistance to fluoroquinolones in Enterobacteriacae. However, the lack of AHC AMR data for Campylobacter was an important limitation to interpretation. Improving capacity for assessing and reporting AMR in Campylobacter might add public health value to AHC's work. As a final example, examining AHC and CIPARS data side-by-side graphically illustrates that ceftiofur resistance appears higher in AHC than in CIPARS Enterobacteriaceae isolates from turkeys, as well as that the CIPARS program was restricted to retail meat during 1 year (2013). The comparison suggests that if a detailed review of AHC data shows limited sampling bias (i.e., AHC data represent multiple premises over many years), increasing active surveillance for ceftiofur AMR in turkeys in BC might be of comparative priority.

- Data from sick animals leads to high estimates of AMR
- Small sample sizes for some samples types
- Improving capacity for assessing and reporting of AMR
- Limited sampling bias

Collineau et al. 2019

Yet, there are a number of challenges that need to be addressed before the transition can be effective. There is still a lack of knowledge translation between bioinformaticians and risk assessors, with bioinformaticians having a limited understanding of the type of

- Knowledge translation gap between parties

LMICs shoulder the bulk of the global burden of infectious diseases and drug resistance but their surveillance systems tend to be weaker than those in high-income countries (HICs), because passive surveillance cannot be integrated with routine casemanagement of patients easily in many areas. This problem has been circumvented to an extent in TB, malaria and HIV AMR surveillance by using active approaches to surveillance in LMICs and gathering data intermittently to provide a snapshot of the situation. However, achieving high coverage of all LMICs and complying with the recommended frequency of surveillance has been difficult. A review of the HIV, TB and malaria surveillance systems in 2011 suggested that one risk of integrating surveillance into routine activities was that high-quality implementation was less likely.17 By contrast, GLASS is based on building up or strengthening traditional models of passive case-based surveillance to generate data, as in HICs. Priority pathogens, drugs and specimens for surveillance are named but, unlike the other networks, GLASS does not specify minimumsample sizes or detailed selection criteria for target populations. Responsibility for quality management is devolved to national reference centres rather than a supranational body. Member states are requested to submit their AMR data to directly on primary TB specimens without an intermediate culture step.18 Molecular surveillance for drug resistance in other bacteria remains some way off but should bemade a high priority in order to simplify surveillance in LMICs

- Difficulty in recommended frequency of survellance
- Responsibility given to national reference centres rather than supranational
- No intermediate culture set up

Assessing the representativeness of AMR surveillance data presents a particular challenge. This will be affected by the geographical location and number of sentinel sites, the number and characteristics of individuals sampled, prior treatment history, the incidence of the target pathogen and the methods of detection. WHO/IUATLD has developed its surveillance methodology to the point where it uses survey data to estimate MDR-TB incidence worldwide but this is exceptional for the global programmes. The global report on early warning indicators of HIV drug resistance states that data from most countries cannot be considered as representative due to the way in which the clinics sampled were selected.11 In malaria therapeutic efficacy studies in hightransmission settings, children less than five years of age are studied since they have the lowest levels of acquired immunity to malaria to give a 'worst-case scenario' depiction of drug efficacy. AMR surveillance for the most commonly encountered bacteria, as it has been practised to date, presents more problems than for other pathogens because of the lack of agreed case definitions and standardized sampling methods. An analysis comparing trends in Escherichia coli resistance from 1997 to 2001 reported by the global Meropenem Yearly Susceptible Test Information Collection (MYSTIC) and SENTRY pharma networks showed that, despite collecting isolates from similar geographical areas, estimates of nonsusceptibility from MYSTIC were consistently higher than those from SENTRY. However, further analysis revealed this was due to a higher proportion of isolates from patients in ICUs inMYSTIC

- Representativeness of AMR data is a challenge
- Characteristics not taken into account
- Characteristics not taken into account

AMR surveillance in animals is still in its infancy, with the exception of foodborne infections, but some strategies have been piloted in LMICs under the guidance of the WHO Advisory Group on Integrated Surveillance of Antimicrobial Resistance (AGISAR). The challenges are great, e.g. progress towards standardizing AST breakpoints in veterinary microbiology is far behind that made in humans.

- Standardization still being deliberated

A successful AMR surveillance network should generate up-todate comparable, representative, high-quality data on pathogens of concern fromthe target population(s). It should be able to detect and track unexpected events including outbreaks in real time, have rapid, effectivemechanisms for communication and reporting, and have a responsible data-sharing policy. A network needs strong leadership and coordination, and it should influence guidelines and policy and ultimately impact on human and animal health. Very few networks were instigated to specifically monitor intervention programmes, e.g. the International Nosocomial Infection Control Consortium. Linking surveillance activity to interventions to combat drug resistance has the potential to increase their impact

- Surveillance should generate representative high quality data
- Effective mechanisms for data sharing
- Strong leadership

Pharma networks produce high-quality data, but theymay not be representative and these networks do not usually support laboratory capacity building in LMICs or influence policy and guidelines. Purely academic networks also produce high-quality data; they often target a clinical or policy question, but they too have limited influence on policy and their sustainability is reliant on external funding. Most of the networks are slow to report their findings and do not give unrestricted access to their data. The experience of the larger global programmes for AMR surveillance in TB, malaria and HIV suggests that options for more active surveillance may need to be considered in order to gather comparable useful data from low-income countries before reliable case-based surveillance can be established.

- Pharma may not produce representative data
- Limited laboratory capacity in LMICs
- Limited influence on policy
- Limited sustainability
- Limited scope of surveillance

Bale et al. 2010

Our study illustrates important improvements in individual laboratory and network capability achievable through application of these established principles over time when the 'correct ' result was ultimately obtained for 95.6% of tests performed in the Indian GASP in 2007. This highly satisfactory outcome was achieved through continuing close collaboration between the WHO collaborating centre and the SEAR regional reference laboratory on the one hand, and the liaison of this same laboratory, in its role as the Indian GASP coordinating laboratory, with Indian GASP participants on the other. A single proficiency survey for AMR in N. gonorrhoeae in the UK with six gonococcal strains sent to 411 laboratories in 1986 saw an 11% error rate [14] for gonococcal sensitivity tests. In a survey from 14 laboratories from Western Europe [15], overall concordance using all methods (disc diffusion, agar dilution, E test) was highest for ceftriaxone (93%) and lowest for tetracycline (72%) which is in contrast to our study. Disc diffusion gave the lowest overall concordance (72%) compared to MIC determination (>88%) by either method in the study from Western Europe [15]

- Improvements in capability through principle
- Collaboration between WHO and SEAR

The Indian GASP EQAS also confirmed the value of repeat challenges with identical but anonymized strains accompanied by general anonymous feedback to participants and detailed comment to individual centres. The importance of this feedback, including that in relation to technically correct, but wrongly interpreted laboratory data was especially noted. The outcome of this study suggests that this continuing educative process, conducted anonymously by dialogue between the coordinating centre and participants on an individual basis, means that the Indian EQAS has extended its programme beyond that of proficiency testing to establish a viable network forum that has substantially strengthened the Indian GASP. Other national and regional GASP EQAS programmes obtained similar results using, initially, monthly challenges accompanied by similar feedback and network-based educative processes [4, 16]. However, the Indian GASP EQAS was restricted to annual evaluations because of limited resources. Improved laboratory performance may have been achieved earlier if more frequent challenges, accompanied by education and consultation, had been possible.

- Feedback from participants
- Anonymous educative process
- Dialogue between coordinating centre and participants
- Network based educative process.
- Limited resources

One major limitation of the disc-diffusion method revealed here was the failure to distinguish strains with 'decreased susceptibility ' to ceftriaxone in a high proportion of tests. This finding provided valuable field data on the lack of utility in peripheral centres of the proposed system for ceftriaxone disc-diffusion testing that had worked well in a central reference laboratory. Recently additional published information provided further insights into the possible reasons for the failure of this trial of ceftriaxone discdiffusion testing. A number of inter-related molecular changes occurred in multiple genes that are responsible

- Failure to distinguish strains
- Lack of ultility in peripheral centres

information required for QMRA modeling, and risk assessors having poor comprehension of how WGS data are analyzed, including what type of information can be generated and any limitations on these analyses. Risk assessors and epidemiologists need to be better trained in next generation sequencing techniques and molecular epidemiology (De Lamballerie, 2009; Arts and Weijenberg, 2013). Risk assessors also have to be proactive and should be involved from the early stages of development of WGS databases and bioinformatics tools, so that they can make their needs more explicit and influence the type and format of data being generated, rather than simply acting as 'end-users' of WGS data. Input from both bioinformaticians and risk assessors should also be considered while designing surveillance programs and data collection initiatives to ensure these are capturing the data needed to perform the types of analyses described in this review.

- Poor comphrension of data/analysis
- Better taining regarding techniques
- Both be considered in designing surveillance prrograms

The different procedures through which WGS data are generated and analyzed have a strong influence on the results. For example, the choice of different sequencing platforms introduces systematic biases that have an impact on the inferred phylogenies (Kaas et al., 2014). With foodborne AMR pathogens easily traversing jurisdictions or countries, it can be problematic when different laboratories use different procedures and do not arrive at identical conclusions (Pightling et al., 2018).

- Different procedures in WGS data
- Problematic as different laboratories use different procedures

Efforts toward harmonization and standardization of WGS techniques are ongoing, for example under the Global Microbial Identifier (Moran-Gilad et al., 2015) and the PulseNet International initiatives (Nadon et al., 2017), but a consensus on methods, quality measures and thresholds for data generation and analysis of foodborne AMR pathogens has yet to be established (Lüth et al., 2018). In the absence of a current consensus, it is important to ensure all sequenced genomes are made publicly available where possible, or available privately across jurisdictions, as to allow researchers to repeat analyses within their pipelines or under their parameters. Standardization of epidemiological data (also referred to as 'metadata') accompanying WGS data is also critical to provide requisite contextual information necessary to any microbial risk assessment activity (Hill et al., 2017). The Genomic Epidemiology Ontology (GenEpiO) Consortium seeks a global standard for the metadata associated with WGS data, including laboratory, clinical and epidemiological data fields (e.g., strain names harmonized and compatible with previous classification schemes), as well as existing food categories (Griffiths et al., 2017). Data sharing and inter-operability (e.g., between laboratory and epidemiological databases) should also be addressed as 2http://www.genomicepidemiology.org/ key priorities to ensure the efficient use of WGS data (Pightling et al., 2018).

- Harmonization through Global Microbial Identifier and other International initatives
- Consensus has yet to be made
- Standardization of epidemiological data accompanying WGS
- Consortium for global standard
- Data sharing and interopability
- Global standard

Chandrasekera et al. 2015

Implementing MHS-wide AMR surveillance has proven more challenging due in part to the sheer size of the system (more than 230 MTFs) and the stovepipe approach to data handling. Two strategies have been taken to address the need for AMR surveillance across the MHS: one by the Walter Reed Army Institute of Research with the Multidrug-Resistant Organism Repository and Surveillance Network and the other by the Navy Marine Corps Public Health Centers EpiData Center (NMCPHC-EDC). Together, the Multidrug-Resistant Organism Repository and Surveillance Network (MRSN) and EpiData Center (EDC) form the Antimicrobial Resistance Monitoring and Research Program (ARMoR).

- Implementing surveillance difficult due to size
- Collaboration between sectors

Collaboration between the EDC, theMRSN, and theMTFs, serves as a quality assurance process to ensure that MDROs are being captured. When the EDC identifies a suspect MDRO of interest, the MRSN and the MTFs can cross-check their records and determine if the isolate should be obtained by the MRSN for characterization (if this has not already been done).

- Quality assurance to capture MDRO
- Collaboration between sectors

To increase MTF participation in the NHSN module pertaining specifically to MDROs, the EDC is piloting an effort that leverages its capability to access, identify and monitor resistant organisms from Composite Health Care System microbiology laboratory data, and report this AMR data on behalf of all MTFs to the NHSN

- Increase participation
- Leverage data in system and report on behalf

Nevertheless, although AMR surveillance programs may be functional at some MTFs, the data collection and analyses are not necessarily done in a standardized manner between facilities, and therefore, data are less directly comparable. To be confident in the interpretation of antimicrobial resistance data across different sites, consistent, robust criteria must be applied for specimen collection, testing methods for organism characterization, and data reporting

- Data collection and analysis not standardardised
- Need for robust, consistent criteria across sites

With regard to laboratory testing, adoption of the nationally recognized Clinical and Laboratory Standards Institute (CLSI) guidelines by U.S. clinical microbiology laboratories,38,39 increases the comparability of data. In addition, use of commercially available automated ID/AST platforms which incorporate CLSI cut-off values for AST which are widely utilized at MTFs can also add an aspect of standardization. However, consideration should be given to a number of potentially data biasing factors with regard to use of automated ID/AST platforms: (1) the type of automated platform in use at each site can differ; (2) MTF ID/AST machine software updates are conducted on different timelines, which complicates uniform reporting of data; and (3) ID/AST platforms in the United States are required to report antibiotic susceptibility results from minimum inhibitory concentration values using Food and Drug Administration-approved breakpoints, however, there is a delay between timing of CLSI and FDA breakpoint [illegible]

- Guidelines increase data comparability
- Commerical platforms increase standardisation
- Platforms can bias data
- Can complicate uniform reporting

for the alterations in susceptibility to extendedspectrum cephalosporins [18, 19]. However, the impact of these changes (both known and unknown) affects the extended-spectrum cephalosporins unequally. The injectable antibiotic ceftriaxone is least affected in terms of MIC increases and clinical treatment failure. The orally administered members of the group, such as cefixime and ceftibuten, are associated with greater relative MIC change and treatment failure [9, 18]. Consequently, results of susceptibility testing for ceftriaxone cannot represent the susceptibility status of all members of this group of antibiotics. Alternative testing methods relevant to all the different cephalosporins have therefore been developed and will be re-evaluated under peripheral centre conditions. Until these methods have been fully verified, the importance of using accurate MIC methods and appropriate internal controls for detecting reduced susceptibility to cephalosporins should be restated [11]. Finalization of these methods is relevant not only to India because of the detection of cephalosporin 'non-susceptible ' strains of N. gonorrhoeae there [20], but also more widely because of the increasing spread and prevalence of these gonococcid earlier if more frequent challenges, accompanied by education and consultation, had been possible.

- Alternative methods for verification
- Education and consultation

AMR surveillance of N. gonorrhoeae is critical for public health purposes [1, 6], but the data must be rigorously validated by ongoing appraisal of the quality of the results of the surveillance if it is to be used with confidence to alter treatment schedules [1, 6]. While the results of the Indian GASP EQAS challenges were encouraging, there is a clear need for continuing network-based educational programmes that emphasize adherence to proper laboratory testing methods, the importance of quality control, and the basic concepts of quality assurance, the latter including increased frequency of challenges accompanied by relevant feedback [6]. The lessons from the Indian GASP EQAS can provide helpful information in other settings at a time when there is a demonstrated need for more and better quality AMR data to assist in the control of gonorrhoea.

- Data validation by appraisal
- Clear need for educational programmes
- Adherence to testing methods
- Quality control and assurance with feedback

Bennani et al. 2021

We identified 30 key organisations directly involved in the national surveillance system (Table 1). Out of these, 15 belong to government organisations, nine to the private sector and six to international institutions.

- 30 organisations involved in surveillance

Planning, data collection, analysis, interpretation and reporting are conducted by each sector separately with the exception of Scotland where there is an OH surveillance report (Scottish One Health Antimicrobial Use and Antimicrobial Resistance Report [SONAAR]).19 There are also variations in the surveillance systems in the four UK countries, especially in humans where the implementation of the AMR strategy is fully devolved. In animals, there is a UK wide annual surveillance report for AMR and AMU, which is the Veterinary Antimicrobial Resistance Surveillance (VARSS) report,while in humans there are separate surveillance reports in each UK nation.

- Separation of activities
- OH surveillance report
- Variation in AMR system

In terms of cross-sectoral collaboration, the most important activity is the ResAlert contingency plan, which refers to the response upon identification of a resistant bacterial isolate from an animal considered to pose a potential risk to human and/or animal health.20 This is a UK wide plan initiated in 2015 and coordinated by the VMD in collaboration with government agencies covering human and animal health, food safety and the devolved administrations. Depending on the hazard identified, relevant advisory committees are notified by the relevant agencies.20,21 Other examples of cross-sectoral collaborations include the UK OH reports and the DEFRA Antimicrobial Resistance Coordination (DARC) group. The UK OH report on human and animal antibiotic use, sales and resistance was produced by PHE and the VMD for the first time in 2015 and this helped to align the data across sectors and included also recommendations to address data limitations and to improve integrated analyses.22 A second report was published in 2019, which in addition to the data included in the first report had also data on AMR in isolates from retail meat.21 The DARC group coordinates, advises and reviews DEFRA activities on AMU in animals and AMR Veterinary Record 5 of 12 in micro-organisms from feedingstuffs, animals and food. This advisory group has representatives fromscience and policy fromEngland, Northern Ireland, Scotland and Wales covering human and animal health and the environment.3

- ResAlert contingency plan
- UK wide plan
- Different sectors encompassed
- Advisory group has representatives from science and policy
- UK OH report aligned data across sectors
- Addressed data alimitations and integrated analyses
- DARC group coordinates DEFRA activities

The UK contributes to European and Global surveillance through different programmes that are represented as follows: 1. The European Antimicrobial Resistance Surveillance Network (EARS-Net): Run by ECDC and collects data onAMRin eight bacterial pathogens from invasive infections in humans (Streptococcus pneumoniae, Staphylococcus aureus, Enterococcus faecalis, Enterococcus faecium, Escherichia coli, Klebsiella pneumoniae, Pseudomonas aeruginosa and Acinetobacter spp.).23 2. The European Surveillance of Antimicrobial Consumption Network (ESAC-Net): Run by ECDC and collects data on the consumption of antimicrobial agents in humans.23 3. The European Food and Waterborne Diseases and Zoonoses Network (FWD-Net): Run by ECDC and collects data on AMR in Salmonella spp., Campylobacter spp. and Shiga toxin/verocytotoxinproducing E. coli (STEC/VTEC). The data contribute to the EFSA/ECDC annual summary report. To facilitate comparison of the data between countries and with results from the AMR monitoring performed in isolates from animals and food products, EU protocol for harmonised monitoring of AMR in human Salmonella and Campylobacter isolates was developed by the ECDC.24,25 4. The EU harmonised monitoring of antibiotic resistance in zoonotic and commensal bacteria from healthy FPAs and food: Run by EFSA and collects resistance data on Salmonella spp. and Campylobacter spp. and indicator E. coli and Enterococcus spp.24 5. The European Surveillance of Veterinary Antimicrobial Consumption (ESVAC): Run by EMA and collects data on the use of antimicrobial agents in animals.26 6. The Global Antimicrobial Resistance Surveillance System (GLASS): Run by WHO and collects information on resistance among human priority bacterial pathogens from clinical specimens considered a threat globally.18

- European and global contribution
- Surveillance network ran by ECDC
- Data collection of agents, food, and disease
- Summary reports
- Comparability of data
- GLASS ran by WHO collects information on AMR

In animals, the veterinary antibiotic resistance and sales data monitoring programme is commissioned 3 https://www.gov.uk/government/groups/defra-antimicrobial-resistancecoordination- darc-group. and funded by the VMD, which is an executive agency of DEFRA. The VMD is also responsible for the publication of the VARSS report, which includes data on antibiotic consumption (sales data and usage data), and antibiotic resistance from animals in the UK.9,27,28

- Funding by VMD

updates

The centralized laboratory services of the ARMoR program can provide standardization for resistant bacteria collected within the MHS and allow uniform interpretation of data and comparison of data across regions.

- Data standardisation by centralised laboratory service
- Increases comparability across regions

In addition to employing centralized molecular characterization, the ARMoR program is in the process of adopting the NHSN case definitions and testing guidelines which will enable data comparisons across the civilian sector as well.

- Centralised molecular characterisation
- Adopting case definitions enable data comparisons

However, it is feasible to harmonize across labs, as a number of key pathogens are already tracked by all facilities (e.g., methicillin-resistant Staphylococcus aureus, Klebsiella spp, etc.), and although antibiograms may differ by MTF, standardizing minimum data elements for inclusion on an antibiogram is possible. Additionally, if the same standards are being held (CLSI) by all microbiology laboratories, this would be a step closer to being able to compare data across MTFs. At a DoD-wide level, the ARMoR Program is a significant achievement and resource for the military and has generated substantial MDRO surveillance data in the last 4 years that allows uniform interpretation of data. With continued support, MTFs can provide data of higher quality to the ARMoR program while the program continues to provide central reference hub capabilities.

- Harmonization across labs possible
- Standardising data elements for inclusion on an antibiogram
- CLSI standards for all laboratories

Fluit et al. 2006

A certain sample bias is present in every surveillance system, because different criteria are used by different physicians when selecting patients for microbiological investigation [8,9]. In addition, since isolates or data may be used in several studies, there are dangers in concatenating results or comparing the studies [10]. The longitudinal component of these studies may also be a problem, in that some centres may withdraw from a multicentre study. The problem of biased isolate inclusion is even greater for surveys of antibiotic resistance in the community. Usually, only samples from patients with persistent infections or infections refractory to treatment are referred to a central laboratory by a general practitioner, and it is not clear how representative these isolates are of strains causing communityacquired infections in general. Therefore, neither hospitals nor isolates may give a clear representative perspective of the global danger of resistance. Although longitudinal studies that include the same hospitals and types of isolates give some indication concerning trends, it is risky to provide guidance to clinicians for empirical antibiotic therapy based on this information, because of differences at the local level. Therefore, local resistance data must be available to physicians working in hospitals; regional data concerning community pathogens must be available to general practitioners; and national data must be available to healthcare policy-makers. Moreover, European and worldwide data must be freely available to all to provide early warning signs. One example of the need for a global information system is vancomycin-resistant Staphylococcus aureus. To date, there have been three reports of true vancomycin-resistant S. aureus [11–13], and because such bacteria pose a serious health-threat, early warning is very important.

- Sampling bias in surveillance systems
- Samples from persistent patients
- Isolates may not give a clear representative perspective of the global danger of resistance

Good-quality susceptibility data are essential to detect trends, as well as new and rare resistance phenotypes. Unfortunately, the quality of resistance data, especially from clinical laboratories, is often questionable [13–16]. Quality control of the susceptibility data is therefore crucial. A further complication in Europe is the use of different definitions for 'resistant': i.e., when is an isolate 'susceptible', 'intermediately-resistant' or 'resistant' to an antibiotic?

- Good quality susceptibility data needed
- Questionable quality of data
- Definitions differ for resistance

With few exceptions, surveillance efforts in the veterinary sector are limited and the different programmes are not coordinated. Furthermore, the criteria for testing isolates differ widely among countries, as do the antimicrobial agents tested. As a result, there is no clear picture of the extent of antibiotic resistance among isolates of veterinary origin [9]. The same is true for isolates from food; in many cases, it is only in following cases of food poisoning that an attempt is made to track antibiotic-resistant isolates to their source. The cost of adequate surveillance, despite the issue of food safety, and the small profit margins in the veterinary sector make the financing of surveillance in the veterinary and agriculture sectors difficult. Finally, although antibiotic resistant isolates have been described, no major efforts have been made to assess the reservoirs formed by pets and the environment.

- Veterinary sector limited
- Criteria differ from country to country
- Between sectors there is overall difference
- Cost of adequate surveillance

An additional problem is the limited availability of genetic data concerning antibiotic resistance. In most cases, it is unknown whether the spread of resistance is caused by the clonal spread of a resistant strain, or by the horizontal transfer of resistance determinants among different strains and species. Reference centres have responsibility in this area, but are often not well-equipped or supported to perform such research. Despite (e.g.) the HARMONY [17], GENE [18], CAMPYNET (http://campynet.vetinst.dk) and PULSENET (http://www.cdc.gov/pulsenet/) projects, in which a start has been made on the development and implementation of standardised and exchangeable typing methods to monitor clonal expansion, more work needs to be done in this field. A major problem is that standardised protocols are not implemented in all studies, and that testing for clonal spread is limited because it is costly and time-consuming. Another problem is that the spread of antibiotic resistance through horizontal transfer is rarely monitored. This includes spread among different reservoirs. Some surveillance programmes have reported data on a limited number of isolates and resistance determinants, but no large-scale structured efforts have been made to study such spread. The WHO recently identified this lack of information concerning microbial genetics and ecology in antimicrobial-resistant bacteria as a gap in current knowledge and hence a key need [3]

- Limited availability of genetic data
- Reference centres have responsibility but are not well equipped
- Other systems have developed and implemented
- Standardised protocols throughout studies
- Horizontal transfer rarely monitored

Antibiotic consumption Antibiotic sales data are submitted annually by Veterinary pharmaceutical companies to the VMD; this has been a statutory requirement since 2005. The data are presented according to the Anatomical Therapeutic Chemical Classification System for veterinary medicinal products (ATCvet) and analysed using a Population Correction Unit (PCU). For usage data, the livestock industry and VMD developed collaboratively antibiotic usage data collection systems.9 This initiative is coordinated by RUMA;4 the UK agriculture and food industry alliance that promotes responsible use of medicines in farm animals. RUMA has 24 members including the British Veterinary Association (BVA) and the Agriculture and Horticulture Development Board (AHDB). Antibiotic usage data refer to the amount of ABs purchased, prescribed and/or administered. Usage data are available for meat poultry (chicken, turkey and duck), pigs, gamebirds, laying hens, dairy sectors, beef, trout and salmon industries.27,28 Reporting coverage of AB use differs between sectors with coverage of 90% or more for poultry, laying hens and aquaculture sectors, 30% coverage for dairy and 5.5% in beef sector. For companion animals, antibiotic usage data are not systematically collected but the VMD funded studies to investigate it and the results were included in the UK-VARSS 2016.9,29,30 These studies looked into AB prescriptions using the data that were extracted from practice management systems by VetCompass and the Small Animal Veterinary SurveillanceNetwork (SAVSNET) system that aremanaged by the Royal Veterinary College and the University of Liverpool, respectively. Antibiotic sales data in dogs and cats were published in the UK-VARSS 2018.28

- Statutory requirement for veterinary pharmaceutical companies
- VMD developed collaborative antibiotic collection systems
- Data differes across sectors

This study showed that the surveillance system for AMR and AMU has a multitude of stakeholders, processes, activities, data sources, within the human, animal and food sectors.

- Multitude of stakeholders
- Data sources
- Various sectors

The study highlighted that there are variations in the surveillance system for AMR and AMU between the four UK nations, and identified integration points as well as opportunities for further integration. In animals, surveillance of antibiotic consumption ismainly based on sales data, however these data are an estimate of the quantity of ABs used and do not enable detailed analyses such as the consumption by animal species or production system. Although AB usage data are submitted voluntarily by some livestock stakeholders, there are still substantial differences in reporting coverage between livestock sectors. For AMR surveillance in animals, one of the main programmes is the EU harmonised monitoring. However, with the uncertainties around Brexit, it is not known whether the UK would continue to contribute to this programme. In humans, surveillance of AMR is based on voluntary reporting by hospital microbiology laboratories. Although there is a high reporting rate, weaknesses of this system remain due to incomplete data collection. Also, resistance data are collected from clinical cases and do not include isolates from healthy persons. Regarding AMU in humans, data from private prescribers are not reported. Having a system that captures data from private healthcare practices is important to assess the extent of AMU from these sources.

- Variation in surveillance systems
- Integration points are different
- Data differences
- Coverage for livestock sectors
- High reporting rate
- Private data not integrated
- Data completeness

There is a lack of data from the environment, which is considered to represent a significant pathway for the transmission of AMR to humans and animals.41 This has been acknowledged by the UK government and one of the priorities in the new AMR strategy is to understand better the role of the environment in the spread of AMR.8 In addition, in 2018, a network of 23 partners (including UK government agencies APHA, Cefas, EA, PHE and VMD) was awarded funding from the Joint Programme Initiative on AMR to identify robust, measurable surveillance indicators andmethodologies for assessing AMR levels in the environment.6 The UK contributes also to European and global surveillance through different programmes.18,24,26,42 This collaboration helps harmonise how the data are collected and shared and allows comparison between countries. However, there remain significant differences on how these data are collected and analysed between the different sectors including the use of different laboratory methods and different interpretative criteria for resistance, which hinders cross-sectoral comparisons.24,25

- No environmental data
- UK government - priority in strategy
- Funding by joint programme initatives
- Data selection differences
- Interpretative criteria for resistance

At the level of data collection, integration can be increased by including resistance data from companion animals and from locations in the environment; by including isolates from vegetables, fruits and herbs; by collecting antibiotic usage data from species that are not covered and by increasing coverage in species with low coverage. Integration can also be increased by harmonizing data analyses methods and interpretation across sectors to enhance the comparability of the information produced. This was highlighted in the new AMR strategy and one of the commitments is to "explore how to coordinate and harmonise surveillance schemes across the different sectors".8 At the level of information dissemination, integration can be increased by moving from producing different surveillance reports for each sector to integrating all surveillance information in a joint report. Also, these surveillance reports need to include data for the whole of the UK.

- Further integration by incorporating more data
- Higlighted in AMR strategy and comittments

Chandy et al. 2013

There was a lack of enthusiasm from GPs and pharmacists in participating in the surveillance programme. Permission from GPs was difficult to obtain at times. Some believed that granting permission would lead to inspection of their prescriptions by the authorities. Permission from pharmacy shops was also difficult to obtain since some owners feared the possibility of subsequent tax audits if sales details were released. Selection of facilities was therefore based on feasibility

- Difficulty in engaging GP and pharmacists
- Permission from pharmacies were difficult to obtain

On many prescriptions the handwriting was illegible. Deciphering and identifying brand names took time and effort

- Difficulty in data collection

Poor prescription and dispensing practices contributed to the challenges. In some facilities, medicines were prescribed on small pieces of paper or outpatient charts. In pharmacy shops and government hospitals, documenting antibiotic encounters was quite difficult in crowded situations.

- Poor dispensing
- Difficult to chart data

Although the optimal criteria for good surveillance programmes have been well-established in the scientific literature [4], practice shows that this ideal has not been achieved. Two main causal factors have been identified: lack of financial resources and lack of standardised methods. Despite the limitations of current and past surveillance programmes, much has been learnt regarding antibiotic resistance among pathogens such as Mycobacterium tuberculosis, S. aureus, Vibrio cholerae, Salmonella and the other Enterobacteriaceae, Pseudomonas aeruginosa, pneumococci and enterococci [19].

- Optimal criteria for surveillance has not been established
- Due to lack of financial resources
- Standardisation of methods

One approach to overcome this problem would be to have all isolates tested in a central reference laboratory. In this way, each isolate could be analysed using the same methods, interpretation criteria and rigorous quality control. However, the logistics and cost of shipment are prohibitive for such a proposal. A better approach would be to send the susceptibility data for isolates from each patient to a reference centre, and ensure that these data are quality-controlled

- Isolates tested in central laboratory
- Standardisation of testing procedures
- Logistics and shipment may be a barrier
- Reference centre for isolate processing

In the short term, all large hospitals should provide annual reports on resistance levels. The programme and criteria set by the European Antimicrobial Resistance Surveillance System (EARSS) may be a useful starting point. The objective of the EARSS is to amass and summarise comparable and reliable antimicrobial resistance data to benefit public health across Europe, taking into account laboratory methods as well as epidemiological principles [20]. However, the EARSS is limited by the number of microorganisms and participating hospitals. A good approach, therefore, would be to collect data for a larger number of microorganisms from all EU member states for analysis by a central organisation, which might become part of the European Centre of Disease Control (ECDC).

- Hospitals reporting
- EARSS for standardisation of criteria
- Limited by participation

Standardised methodology yielding quantitative data to enable the detection of small shifts in susceptibility would be ideal, but this cannot be achieved in practice, because a number of different techniques exist for antibiotic susceptibility determinations (e.g., VITEK, Phoenix, Walkaway, disk-diffusion assays).

- Standardised methodology for susceptibility

Quality control is therefore of extreme importance. Organisations that provide external quality control already exist (e.g., NEQAS, EARTHNET), but operate on a voluntary basis and with contributions from the participants. A much larger effort is required to monitor the performance of all laboratories providing antibiotic resistance surveillance data. Perhaps it can be based on the good example of effectively addressed quality control issues found in the EARSS programme [8,22]. The EU should help to fund these activities

- Quality control is of extreme importance
- Already exist but voluntary with contributions
- Quality control to be addressed
- EU funding of activities

A number of European countries, including Finland, France, Germany, Spain, Sweden, The Netherlands and the UK, have some form of surveillance of veterinary isolates, or have conducted at least some surveillance studies in the veterinary field. However, the most extensive programme is in Denmark. The Danish Integrated Antimicrobial Resistance Monitoring and Research Programme (DANMAP) addresses the problem of antibiotic-resistant bacteria in animals, food and humans [23]. The programme has four objectives: to monitor trends in resistance among bacteria from animals, food and humans; to monitor the consumption of antimicrobial agents in animals; to determine the association between consumption and the occurrence of resistance; and to model the transmission of resistance from animals to humans. The programme examines representative isolates of human and animal pathogens, zoonotic bacteria and indicator bacteria. Animal isolates are derived from both healthy and diseased animals, and the antibiotics are representative of the major classes of antibiotic. Furthermore, antibiotic consumption data are collected for food animals according to the VETSTAT programme and are recorded for each herd. Ideally, the veterinary sector should follow the DANMAP/VETSTAT approach. An important route for infections and possible transfer of resistance is via food. Therefore, improved microbial food safety will reduce the number of episodes of food poisoning, and thereby the need for treatment and the opportunity for transfer of resistance. In order to achieve improved microbial food safety, the continued development of alternative methods of animal husbandry in the veterinary sector, with reduced or optimised use of antimicrobial agents, will be necessary.

- Surveillance in veterinary field
- DANMAP addresses holistic AMR
- Integration of multiple programmes

Frost et al. 2021

The UK's Fleming Fund is one of the largest multinational investment programmes aimed at addressing and strengthening critical gaps in AMR and AMC surveillance in LMICs. The Fund, a £265 million investment supported by the UK Department of Health under its overseas development assistance programme, UK Aid, is built on a One Health approach and provides funding at institutional, national and regional levels

- Fleming fund used to strengthen AMR surveillance in LMIC
- One Health approach at a institutional, national and regional level

The Regional Grants programme in Africa has the following implementation partners: CDDEP, the African Society for Laboratory Medicine (ASLM), IQVIA [formerly QuintilesIMS (Intercontinental Medical Statistics)], Africa Centers for Disease Control and Prevention (CDC), West African Health Organization (WAHO), the East Central & Southern Africa Health Community (ECSA-HC) and Innovative Support to Emergencies, Diseases and Disasters (InSTEDD). The aim of the Regional Grants programme is to collect retrospective AMR and AMC data from public and private laboratories, pharmacies and health facilities in 14 African countries in order to establish a baseline understanding of the AMR burden in these countries. Additional analyses will also seek to identify and better understand drivers of resistance—such as socioeconomic and clinical factors and vaccination coverage rates—at the individual and community levels

- Regional grants in Africa for funding
- Collect retrospective AMR and AMC data
- Establish baseline for AMR burden

Patients visiting the facility in a given period was the denominator for DDD methodology. This may make it difficult to compare our findings with those of studies using fixed populations or inhabitants as the denominator

- Data cannot be compared

Bulk antibiotic data were much quicker to collect than percentage antibiotic encounters. However, many facilities did not have or would not reveal sales records. Even when available, sales records were not necessarily organized in a systematic manner and therefore appeared incomplete.

- Difficulty in sourcing data

The data collected were based on prescriptions, dispensations and bulk sales. Actual consumption and patient compliance, which is necessary to reveal true antibiotic burden, were not determined

- Representativeness of data

Data collection for such a large number of patients over a 2 year period resulted in a huge dataset. Customized software development, data entry and data verification took a great deal of time. Intense effort over a period of years was required for data cleaning and sorting out the analytical issues due to the methodological issues involved

- Huge dataset produced
- Data verification was problematic

There is a need for relatively sophisticated data management software. Software for drug use surveillance customized to the needs of this project had to be developed. This software can be used for similar studies in the future.

- Data management software needed

A multidisciplinary team approach was needed for proper conduct of surveillance. Such teams are harder to assemble for long periods than single-discipline teams. Projects involving multiple disciplines therefore become a challenge in terms of sustainability

- Multidisciplinary teams needed for surveillance
- Sustainability of project when involving multi-disciplinary teams

This surveillance system was set in place with project finance support. Sustainability requires a continuous source of funding, especially for the salaries of data collection officers. Political will to fund long-term surveillance is imperative

- Funding needed for data collection
- Sustainability needs political willingness

This surveillance assessed antibiotic encounters only in humans. The challenge will be to survey not just human antibiotic use, but also antibiotic use in animal husbandry, agriculture and other industries.

- Data also necessary from other sectors

Deckert et al. 2010

In Canada, there is no mandatory reporting of national sales data by pharmaceutical companies and no centralized system for recording/collecting antimicrobial prescriptions (Health Canada, 2002).

- No mandatory reporting of prescriptions to collect data
- Difficulty for representativeness

Considering this complex distribution system, CIPARS considers the optimal AMU surveillance system in Canada to include national sales figures and end-user data. The Canadian Animal Health Institute collects national sales data from member pharmaceutical companies. However, these data are incomplete as they do not include data from non-member companies, generic antimicrobial manufacturers or direct importation of antimicrobials by producers (Government of Canada, 2007). Therefore, in the current context, although expensive, logistically challenging to collect and potentially biased, end-user data are the most feasible option for antimicrobial use surveillance in the Canadian agri-food sector.

- Institute collects data from members
- End user data most pragmatic option
- Data is incomplete
- Does not include data from non-member companies

The CQA program was not designed specifically for the purposes of antimicrobial use data collection and therefore some critical data elements are not included (Anonymous 2007). The utilization of the herd veterinarian to complete the antimicrobial use questionnaire with the producer may improve the quality and validity of the data collected as a result of the ongoing relationship and knowledge that the veterinarian has of that operation. This may pose an advantage over systems where thirdparty veterinarians with no prior knowledge of the operation complete these questionnaires with the producer (Dewey et al., 1997, 1999). In the CIPARS Farm program, the involvement of the herd veterinarian also protects the confidentiality of the participating producer thereby improving candor regarding antimicrobial use although a potential bias toward under-reporting of certain antimicrobials is still possible. This model has been utilized in previous regional research studies (Rajic et al., 2006; Rosengren et al., 2008b).

- Quality assurance not for antimicrobial use data
- Improve validity via utilization of herd veterinarian
- Protects confidentiality

Improvements to the program would include an increased number of participating herds, electronic submission of data, partnership with animal health surveillance initiatives and potentially expansion to other stages of production and other commodities

- Improvements through increased number
- Electronic submission of data
- Partnership initiatives to expand surveillance

Surveillance programmes such as DANMAP that include both national AMU data and prescription data provide more complete information on AMU than the CIPARS Farm program and are able to

- DANMAP - integration of data
- Integration provides more complete data

Fleming Fund activities are complementary to other AMR plans, including the national action plans for AMR control, antimicrobial stewardship programmes, development of empirical treatment guidelines, and frameworks such as the African Union's framework for AMR control [17]. These activities will make primary AMR and AMC data available for the region both by gathering data from previous years and setting up systems for ongoing data monitoring. Through various regional and national grant schemes and programmes, the Fleming Fund aims to establish sustainable AMR surveillance systems in over 30 countries of Asia and Africa and to strengthen partnerships among government, private sector, non-government organisations, academia, and pharmaceutical and research sectors. Systems developed by the Fleming programme can be leveraged for continuing AMR surveillance in the future. As part of these efforts, a comprehensive database of biorepositories is being created, which will help researchers to locate institutions and research facilities where biospecimens are being stored/distributed. It will support the research and development sectors during new product development (vaccines, diagnostic, antimicrobials), advanced testing of pathogens as well as improving clinical care practices

- Activities are complementary to national plans
- AMR and AMC data availability
- Strengthen partnerships
- Support research

he Fleming Fund's stated goal is to invest in systems that generate robust AMR and AMC data and to improve other surveillance platforms that can aid in the assessment of AMR prevalence for WHO priority pathogens in LMICs. Key data collected include patient demographics, clinical profile (including antimicrobial usage), laboratory (isolate) results and AMC information (Table 2). The Africa arm of the project covers 14 countries, namely Burkina Faso, Cameroon, eSwatini, Gabon, Ghana, Kenya, Malawi, Nigeria, Senegal, Sierra Leone, Tanzania, Uganda, Zambia and Zimbabwe (Fig. 1)

- Generate robust AMR and AMC data
- Improve surveillance platforms
- Covers 14 countries

The Alliance is a private sector coalition with a diverse membership across biotechnology, diagnostics, generics and research-based pharmaceutical companies and associations, and with International Federation of Pharmaceutical Manufacturers & Associations (IFPMA) as the secretariat. The Alliance has not established a surveillance platform, however it has committed to share surveillance data generated by its members with publichealth bodies and healthcare professionals, with the aim of under standing resistance trends, improving surveillance capabilities, and generating evidence for appropriate antibiotic and vaccine use. In July 2020, IFPMA announced the launch of an AMR Action Fund with the help of generous investments by the pharmaceutical industry. It aims to bring two to four new antibiotics to patients by the year 2030 [20]

- Private sector collaboration
- committed to sharing data amongst partners
- AMR fund will help with investments by pharmaceutical industry

However, various challenges exist with current systems and it is essential to address them in order to improve surveillance. First, information systems are often paperbased, which makes the task of data collection and aggregation very challenging. Often where the records are electronic, laboratory management systems are not interlinked with the hospital or pharmacy information systems. This results in a lack of information on potential drivers of AMR such as patients' clinical profiles and antimicrobial usage. Second, consumption data cannot be linked to resistance data if both AMR and AMC are not reported at the same geospatial level. Results from national-level aggregation may prevent the development and implementation of subnational/community-specific policies. This becomes further complicated in countries where antibiotics for animal and human consumption are sold through the same channels [21]

- Information systems are paperbased
- Systems are not interlinked
- Lack of meta-data for drivers of AMR
- Further complications when sold through same channels

Third, the lack of standards and established norms for interpreting AMC and AMU data are also a hurdle. While GLASS [5] and ResistanceMap [9] use DDDs as a metric of antibiotic consumption, other databases use different measures. For example, the IQVIA MIDAS database reports standard units (SU), which represent one pill, capsule or liquid equivalent of a particular medicine in kilograms of drugs sold. This can make comparisons between surveillance systems challenging (Table 1). In addition, using sales data can overestimate consumption because people may not consume all of the purchased antibiotic [22]. Prescribing data have similar limitations because not all prescriptions are filled and many of the antibiotics consumed may not have been prescribed [23]. Fourth, financing of AMR control activities is sector-specific and usually focuses on human health. In many LMICs, AMR control activities in non-human health areas are either underfunded or undeveloped. Besides, antimicrobial management in the non-human health sector is often unregulated. Fifth, poor linkages across human health, animal and environmental sectors leads to underestimation of the AMR burden. This may stem out from opposing economic and political aims. Thus, building robust surveillance systems requires close co-ordination between all stakeholders, standardisation of methodologies, capacity building, a One Health approach and strong political will

- Lack of standards
- Standards are not uniform
- Sales data can over-represent
- Prescribing data has limitations
- Robust surveillance systems
- Poor linkages throughout sectors

Hedman, Vasco and Zhang 2020

In resource-limited settings, disease surveillance typically captures a marginal understanding of the system. Surveillance is heavily hindered by the lack of resources and political commitment in supporting AMS agendas [279–281]. Many middle-income countries, especially within South America, fall short of delivering effective AMR surveillance due to inadequate political support [7]. Quantitative metrics of AMU within LMICs are not available [28]. On the other hand, international reports estimating presence of AMU exhibit high levels of variability due to the lack of standardization [28]. Effective interventions are dependent upon a more comprehensive understanding of AMU from established baselines [85]. Many LMICs lack trained personnel and resources to effectively monitor antimicrobial administration [116]. In many LIMCs, bacterial culture independent methodologies (e.g., partial or full genome sequencing) that allow screening drug resistance are not readily available due to high costs [140]. International standards for AMR surveillance are essential for monitoring antimicrobial use in poultry farming. Important projects carried out in LMICs depended on international collaborations with researchers coming from HICs. Moreover, global capacity is required to prevent the fast spread of AMR considering that international borders are crossed by over one billion people each year. AMR is a problem of a pandemic scale that should be better understood by veterinarians, farmers, policy makers, and the general public.

- Marginal understanding of the system
- Hindered by lack of resources
- Hindered by lack of political willingness
- Limited metrics
- Testing not readily available due to high costs
- Depend on international collaborations in LMIC

Ibrahim et al. 2018

Roll-out of the AMR surveillance network is occurring in three phases in order to ensure successful implementation and to effectively prepare sites for sample collection, diagnostic testing, data management and reporting. In order to prioritise sites for roll-out, EPHI, with assistance from ASM, assessed laboratories across Ethiopia in late 2016. A standardised assessment tool was used to better understand the conditions and capacities of potential AMR surveillance sites.

- 3 phases for success
- Prepare sites for sample collection
- Diagnostic testing
- Reporting
- Data management

provide a more detailed interpretation of trends (Jensen et al., 2004; DANMAP, 2009). However, because of a number of AMR and AMU Surveillance in Canadian Finisher Pigs A. factors this type of program is not currently feasible in Canada. Other surveillance programmes that are based on mandatory reporting of national importation and/or sales data provide overall estimates of AMU but may be limited in some cases as a result of a lack of information on species of animal, route of administration or reason for use (Joint Expert Advisory Committee on Antibiotic Resistance, 1999; National Veterinary and Food Research Institute, 2007; NORM/ NORM-VET, 2008, 2009)

- Integration provides more complete data
- Program not feasible as requires mandatory reporting

A potential factor that could impact the interpretation of these AMR data is the relatively few number of Salmonella isolates available each year for antimicrobial susceptibility testing and therefore the limited power of the data. This is compounded when the data are stratified by serotype, in that different serovars have a propensity to express different resistance phenotypes and therefore the serovar distribution can have an impact on AMR prevalence (Gebreyes et al., 2000; Brun et al., 2002; Rajic et al., 2004; Rosengren, 2007a). These data were not robust enough to account for variation at the serovar level in the statistical analyses and therefore this limitation must be kept in mind when interpreting overall Salmonella spp. data

- Few isolates available each year
- Limited data interpretibility
- Different subtypes have impact AMR prevalence
- Data not robust enough to account for variation

A primary concern with a sentinel-based surveillance system is selection bias and the representativeness of the participating herds. The herds and veterinarians in the CIPARS farm program were not randomly selected. A national sampling frame of swine veterinarians and grower-finisher operations does not exist in Canada. As well, producers vary in their willingness and ability to provide accurate and ongoing antimicrobial use information (Dunlop et al., 1998a). If veterinarians and herds that chose to participate are different from those that did not participate, there may be an impact on AMR prevalence estimates and AMU data. Recognizing these limitations, efforts were made to minimize the selection bias while maximizing the quality of the AMR and AMU data that were obtained and the sustainability of the program overall. However, the close similarity of farm level AMR estimates with those from the national abattoir component of CIPARS which randomly samples pigs at slaughter and is not based on producer or veterinarian willingness, indicates that the convenience sample of farms are likely representative of Canadian pig production

- Representativeness of the sample
- Not randomly selected
- Producer willingness to provide accurate AMU data
- Data limitations need to be recognised

There is no ability within the program to validate how closely veterinarians adhered to these selection criteria. The quality of producer records and their willingness to participate were additional critical selection factors. Without the willingness and the ability of participating producers and veterinarians to provide the required records in the appropriate detail, then no AMU data would be available. Therefore, it is a matter of attempting to balance the level of potential bias with the quality of the data. Operations with grower-finisher pigs in the five major pork-producing provinces were the target population. When the CIPARS Farm program was launched in 2006 Statistics Canada was reporting that of the 89% of farms with any pigs, 96% of the hog inventory and 98% of hogs marketed were located in these five provinces. The industry has undergone a substantial reduction since 2006 but at the end of 2008 these proportions were unchanged with the exception that the percentage of hogs marketed originating in these five provinces had increased to 99.6% (Canadian Pork Council, 2009; Government of Canada, 2009).

- Validation of selection criteria
- Bias associated with the quality of the data

Ferguson et al. 2020

A lack of laboratory infrastructure and microbiological expertise in many Pacific island nations has made AMR surveillance unreliable. Most published data are based on studies from major hospitals without details of quality control (QC),5,6 or the testing standard used.7 In addition, external to the laboratory, pre-analytical factors such as proper specimen selection and collection are often deficient. Reliably sensitive blood culture systems are generally unavailable, preventing effective diagnosis of severe bacterial infection and greatly impeding AMR surveillance.

- Lack of laboratory infrastructure
- Microbiological expertise
- Unreliable blood culture systems
- Laboratory tools are deficient

Blood-containing media are required for some species (e.g. Streptococcus pneumoniae and Haemophilus influenzae), which remains problematic where required sheep or horse blood is unavailable. Expired human blood is therefore used widely in the Pacific region; however, this is not an appropriate substitute. The incubation temperature and duration must be correct. Antimicrobial discs must be in-date, of the right strength and stored correctly to avoid degradation. Discs from certain manufacturers have variable quality, highlighting the need to obtain them from reliable sources.14 The technician reading the AST must ensure that the disc inhibition zone is measured and interpreted correctly

- Blood containing media unavailable
- Expired material used but not appropriate
- Correct storage
- AST technician must interpret correctly

Laboratory capacity development in PNG (at Port Moresby General Hospital [PMGH]) and in Honiara, Solomon Islands by the Pacific Region Infectious Diseases Association has focused on improving the training of scientists and establishing standard procedures for the microbiology service. Commercial blood culture systems have been introduced to increase positive culture detection and reduce the turnaround time (reduced from 5 days to 2 days). Clinician education concerning the effective use of blood cultures, AMR and its detection has been conducted as a prelude to the introduction of hospital antimicrobial stewardship

- Laboratory capacity increased with education
- Establishment of standard procedures
- Commerical blood cultures

Internal QC systems have been introduced to provide assurance that AST results are correct. All antimicrobial discs are tested against QC bacterial strains weekly. If the tested zone size is within a specified range, then the process is controlled and the laboratory can issue a valid result. Both laboratories participate in EQA to regularly test unknown isolates from a reference organization

- Internal quality control introduced
- Tested and validation with EQA

A range of issues have had to be addressed, including ordering and supply of consumables and availability of serviced incubators and fridges. The electricity supply has not been a limiting factor in these sites to date.

- Addressing logistic issues

Regular visits and teleconference support to train and mentor technical and scientific staff and assist with supervision by the in-charge pathologist have been useful.

- Mentoring to assist staff

The national reference laboratory at EPHI is coordinating and overseeing all surveillance activities. Focal persons from EPHI have been assigned to each of the initial four sites to support implementation. Implementation began with personnel training for clinicians and laboratory staff. ASM supported EPHI to train laboratory staff from the initial four surveillance sites in basic microbiology, antibiotic susceptibility testing and data management. A model of laboratory mentorship has been put in place. The four sites have begun receiving hands-on mentorship during which experienced laboratorians from ASM and EPHI work alongside the staff from the respective surveillance sites to ensure procedures are understood, followed and refined. To ensure that quality laboratory data is generated, all sites have been enrolled in an external quality assessment programme to evaluate diagnostic and reporting ability. As the quality of specimens sent for testing also affects laboratory data quality, Ohio State University's Global One Health initiative conducted training for clinical and laboratory staff on proper methods for clinical specimen collection. In addition, the training aimed at introducing the purpose and goals of AMR surveillance and broadly conveying the importance of stewardship, infection prevention and control in the context of AMR surveillance.

- Reference laboratory coordinates activities
- Training of clinicians and laboratory staff
- Laboratory mentorship in place
- Quality data assurance through EQA
- International collaboration, Ohio State University

The evaluation identified a number of challenges that EPHI is now taking strides to address. A primary challenge has been the integration of electronic data capture for AMR surveillance into the normal work and laboratory processes at the sentinel surveillance sites. One reason for this is that none of the sites has an established electronic laboratory information management system in use for microbiology and thus staff are not accustomed to inputting data electronically as a regular activity. As an effect of incorporating electronic entry of AMR results into their normal workflow, microbiology staff at some of the sites have reported slower turnaround times for getting laboratory results back to the ordering physicians. Frequent microbiology staff turnover at the sites has also made electronic data entry challenging, as fewer staff are available to run culture and input data.

- No sites have electronic laboratory information systems
- Not accustomed to inputting data in electronically
- Slow turn around for results
- Fewer staff for culture and inputting of data

Laboratory capacity building through on-site mentorship at the surveillance sites and provision of necessary supplies has proved a useful method for ensuring sites are producing quality data. The use of focal persons from EPHI in monitoring progress at each surveillance site has been crucial for identifying problems and supply needs, and for facilitating corrective action on laboratory practices and data reporting. Prior to the evaluation, focal persons from EPHI were visiting sites irregularly and thus creating gaps in communication. Communication between the EPHI focal persons and surveillance sites has since been improved by establishing weekly calls and arranging monthly site visits for the focal persons at EPHI to work on capacity building and quality improvement activities.

- Capacity building through mentorship
- Irregular mentor visits
- Gaps in communication
- Weekly calls and arranging monthly visits

Local procurement of quality microbiology supplies is a challenge in Ethiopia. In the meantime, ASM purchased the needed AMR supplies for EPHI and the sentinel surveillance sites. For long-term sustainability, EPHI has begun working with the Pharmaceutical Fund and Supplies Agency, Ethiopia's central procurement agency, to ensure adequate, quality supplies are available in future

- Local procurement of microbiology supplies is a challenge
- ASM purchased supplies.

Iskandar et al. 2021

Weak laboratory infrastructure
In LMICs, the basic requirements for a functional laboratory infrastructure are not met [24–26]. These include issues with the quality of water, electricity supply, light sources, climate control and ventilation, bio-safety requirements, limited internet coverage and connection speed, lack of soap/alcohol gel, dust, insufficient toilet facilities, inadequate construction that hinders deep cleaning [26]. Inadequate laboratory infrastructure can influence the quality and reliability of pathogen detection and antimicrobial susceptibility testing [27, 28].

- Weak laboratory infrastructure
- Quality of water, electricity, light sources, climate control, internet, ventilation, covarge, soap, toilet, construction
- Inadequate laboratory infrastructure decreases data quality and reliability

Limited staff capacity and training Under-staffing [24–26, 29], lack of dedicated staff, the limited number of trained clinical and laboratory personnel [13, 14, 26, 29] affect the adequacy of data management [30]. Te number of microbiologists and healthcare professionals with expertise in the field is limited [5, 27]. Lack of established professional standards or profile of clinical microbiologists and post-graduates activates is an additional major problem [26]. The limited involvement of microbiologists in staff training and orientation, the lack of governance and leadership, out -of hours calls issues [31], and poor management are challenging barriers [5, 25, 26]. Leaders provide a focal point of activities, advocacy, and championing [5]. With the absence of guidance and logistical management related to stock and waste management and inventory control, the quality of activity and level of performance may be deeply affected [25, 26].

- Staff capacity for laboratory is a challenge
- Lack of established professionals in the field
- Inadequate standards
- Limited involvement, lack of governance and leadership
- Poor management are challenging barriers

Communication issues Poor communication between laboratory staff and the medical team [32] is crucial. Te lack of confidence in laboratory results [26] is related to delays in reporting results [13], lack of relevant reported information such as not providing minimum inhibitory concentration (MIC) [13] in addition to the frequent shortages of diagnostics and reagents. As a result, physicians may rely on clinical judgment and disregard test results [33] or may be reluctant to request a laboratory test [34–36]. Other causes may include the costs and inability of the patient to cover these expenses [13]

- Communication issues
- Poor communication with medical tream and laboratory
- Results in delay and inadequate information
- Costs associated

Limited or lack of availability of consumables, diagnostics, and reagents Omelet and colleagues (2018) [26] discussed the need for diagnostics and reagents adapted to developed countries [26]. High temperatures and humidity are harsh conditions that may affect the quality of diagnostics and reagents that require sustainable and secured cold chain storage. Environmental conditions may be harmful to electronic equipment and other consumables [37]. Shortage of items, lack or limited local manufacturing, substandard local quality [38], and the use of cheap, low quality reagents, and diagnostics may affect the accuracy of laboratory results [25]. Supply chain issues that include strenuous regulations for air shipment delivery may lead to long delays and may challenge the need for secured cold chain storage. These problems in the supply chain may be particularly harmful to products sensitive to temperature and humidity [25, 26, 39]. In poor resources settings, there is a need for robust equipment that is easy to repair and requires maintenance at low cost, which consumes low energy such as electricity-free incubators [40] and autoclaves powered by solar energy [41, 42]. Due to the potentially low return of investment, there may be no special [illegible]

- Lack of consumables for microbiological investigation
- Climate affects quality of consumables and diagnostics
- Shortage of items and substandard materials are an issue
- Supply chain issues with climate

Development of standard operating procedures, in collaboration with scientific staff, and more general quality management systems have also been important. The WHO approach to stepwise implementation of laboratory quality management systems has been useful.

- Developing standard procedures in collaboration with scientific staff
- Developing standard procedures in collaboration with scientific staff

Sheep or horse blood for production of blood containing agar media is generally unavailable. Most laboratories are substituting expired, donated human blood. However, such media usually fail to grow important organisms such as Streptococcus pneumoniae and Neisseria gonorrhoeae

- Generally, material unavailable.

A range of issues have had to be addressed, including ordering and supply of consumables and availability of serviced incubators and fridges. The electricity supply has not been a limiting factor in these sites to date.

- Generally, material unavailable.

Maintaining scientific supervision of QC testing to ensure it is properly performed regularly and that results are formally reviewed and acted on

- Maintenance of QC testing

Continued development of microbiological diagnostic capability and reliability is paramount for clinicians and AMR surveillance. Owing to the unavailability of appropriate media, culture and AST of N. gonorrhoeae and S. pneumoniae are rarely performed. Each Pacific island nation needs to develop the capability for at least one central laboratory to culture these pathogens and subject them to quality-controlled AST or arrange for suitable referral to a nearby country.

- Increase of capacity for diagnostic capacity
- Central laboratory needed

Hannon et al. 2020

For the CanFASP, we identified five key veterinary management groups/consulting practices that provide animal health services and production advice to feedlot producers representing a large proportion of Canadian fed cattle. These veterinary management groups often have proprietary, standardized, data collection and data management software systems (e.g., iFHMS), or use specific commercial software programs to collect individual-animal data at each chute (processing barn, hospital) within a client's feedlot.

- Proprietary software
- Standardised systems
- Data collection and management

Surveillance system administrators must coordinate all moving parts of the system including sample submissions, laboratory analyses, standardized data uploads, summarization and interpretation of findings, timely dissemination of results to stakeholders, random troubleshooting/enquiries, and assisting with ongoing sourcing of funds to support the network. All the above teams are part of the necessary infrastructure for the appropriate and accurate collection and assembly of the surveillance data. Strong communication between these teams is essential for optimum functioning of the surveillance system

- Administrations should coordinate system
- Standardised data uploads
- Laboratory analysis
- Dissemination of resutls need standardisation

To collect the most meaningful data for understanding cattle AMU on a national scale, the objectives and anticipated outcomes of the surveillance initiative must be clear and specific (41). To do this, it is critical that the system is designed with input from those knowledgeable about all aspects of the production system and with input from all key stakeholders. All data come at a cost (i.e., to veterinarians, producers, and the surveillance system), and the cost-benefit of each piece of data must be considered. Understanding the specific objectives and identifying the data points necessary to achieve these, along with the ability to revisit/redefine objectives and data points over time is critical (41). A great deal of effort was spent prioritizing and specifying objectives and outcomes for the CanFASP, including rounds of consultation and discussion within the expert group to ensure that the program scope was broad enough to be useful to the majority of stakeholders, while appropriately focused to be practical and cost effective for implementation.

- Objectives of surveillance need to be clear
- Knowledge to be incorporated about all aspects of production
- Cost-benefit needs to be considered
- Prioritization of objectives through consultation can help implementation

To address challenges around the cost-benefit and logistical issues of implementing a national surveillance project and to ensure that the program was relevant to stakeholders, the Canadian approach developed inclusion/exclusion criteria that aligned with how the majority of feedlot cattle in Canada are raised (Supplementary Table 2). Based on the inclusion/exclusion criteria, each of the participating five major feedlot veterinary management groups provided an anonymized list of their practices feedlots that met the inclusion criteria. The feedlot bunk capacity was also provided for each feedlot. Feedlots were randomly selected from a list of those eligible, after stratification by feedlot size (one-time bunk capacity of 1,000–5,000 cattle, 5,001–10,000 cattle, 10,001–20,000 cattle and >20,001 cattle) and veterinary management group.

- Cost benefit and logistical issues addressed
- Inclusion/exclusion criteria for samples
- Collaboration with veterinary management groups

Specific and established case definitions are required to standardize data across provinces, feedlots and veterinary management groups compiling the data. Within each research project, and then later within the CanFASP, case definitions, response formats and standardized categories for responses were defined and agreed to a priori, to facilitate accurate data collection and summarization. Although the CanFASP definitions will be regularly revisited to ensure relevancy, the intent is to minimize changes in scope to allow data comparability over time.

- Standardised case definition across all data
- Response formats standardised for data collection
- Increases comparability

The CanFASP was designed to have veterinarians work with the feedlots to collect data, as do the other CIPARS farm AMU programs. CIPARS has traditionally worked with veterinarians

- Stakeholder engagement with veterinarians

solar energy [41, 42]. Due to the potentially low return of investment, there may be no special commercial interest in investing in the development of new diagnostics adapted for use in low resource settings [5, 26]. More efforts are being invested in the field and testing innovative and low-cost diagnostic [26]. Omelet and colleagues (2018) [26] also highlighted the suggested use of the blood of a sheep breed adapted to tropical climates as an alternative for sheep blood, horse blood, and rabbit plasma [26, 43, 44]

- Low return on investment
- Need for alternatives

Questionable quality assurance Laboratory Guidance for the selection, sampling, and transport of specimens is absent [26]. There is a limited quality assurance of the process, and no systematic monitoring of quality indicators [39]. Updating standard operating procedures and other documents are challenging [25] due to multiple languages and cultural barriers to the good understanding of such procedures [38]. External quality assurance schemes for all laboratories involved in AMR surveillance is also challenging [14]. Integration of bacteria standardized criteria by international guidelines such as the Clinical and Laboratory Standards Institute (CLSI) and the European Committee on Antimicrobial Susceptibility Testing (EUCAST) into automated antimicrobial susceptibility testing are lacking in LMICs [26, 45, 46]. Guidelines are mainly only available in English, and poorly updated and followed [39] or may be complicated for use by staff that lack expertise in microbiology [26].

- Quality assurance is questionable
- Guidance for sample selection and transport of specimens is absent
- Multiple languages and cultural barriers to understand procedures
- Lack of expertise in microbiology
- Poorly updated guidelines

Relying heavily on funding External funding to strengthen the laboratory capacity and implement AMR surveillance programs is granted by agencies like the Fleming fund, the WHO, and U.S Centers for Disease prevention and Control (CDC) [15]. Funding initiatives' primary goal is to improve AMR surveillance in LMICs. Te United Kingdom (UK) Department of Health launched the Fleming Fund to support low-income countries in developing AMR surveillance systems [47]. Te fund is aligned with the WHO's Global AMR Surveillance System (GLASS) [whom glass] to support the Global Action Plan on AMR [1, 12]. To build capacity in LMICs, the Fleming fund awarded a total amount of 265 million pounds [15] to different countries. Bangladesh, India, Laos, Nepal, Pakistan, and Vietnam have been awarded Fleming Fund country grants to initiate or strengthen AMR surveillance activities [15]. Te challenge resides in sustainability [1] of the progress when funding initiatives are short-term which highlights the need for internal funding and government engagement [14, 15]. Funding can support research and the creation of networks needed in specific circumstances to provide quality data such as the Institute for Health Metrics and Evaluation funded by a joint award from Wellcome, the UK Fleming Fund, and the Bill and Melinda Gates Foundation to gather, map and analyze disease and mortality attributable to drug-resistant infections. There have been multiple calls [48] for the development of a Global Antimicrobial Conservation Fund [49] to support Global Innovation Fund for non-commercial research to further support the provision of basic bacteriology services in low-resource settings [14, 49].

- Heavy reliance on external funding
- Fleming fund, Wellcome fund, are examples
- Sustainability of endeavours because funding is short term
- Funding helps with provision of quality data and address basic bacteriology services in low resource settings

The data challenge on surveillance of AMR in low- and middle-income countries The need for high-quality data One of the fve strategic goals of the WHO global action plan is to strengthen data on AMR through surveillance and research [3]. Data can serve to alert for emerging communicable diseases outbreaks, inform health policymakers, provide the evidence base for developing treatment guidelines and monitor the trends and spread of resistance [12, 13, 15, 30]. Data can inform the implementation of infection prevention and control programs such as antimicrobial stewardship programs [30, 50]. Aggregated multi-sectoral National Surveillance data on AMR help to track trends of resistance across sectors, benchmark data, and implement and update health policy to tackle AMR [50].

- Need for high quality data is there
- Data can serve to inform decisions
- Aggregation of multiple data helps to track burden and inform policy

Despite the evidence base improvement detected in recent years, the laboratory capacity for AMR surveillance in LMICs is still thought of as limited and fragmented [3, 7].

- Limited and fragmented capacity for AMR surveillance

The GLASS enables a standardized data collection and reporting of official national AMR data [12, 15] that secures data reliability and representativeness. The system allows the collaboration of the WHO with existing regional and national AMR surveillance systems through harmonized global standards to produce timely and comprehensive data. Three large regional surveillance networks implemented a report with the participation of LMICs, routine AMR surveillance data on the target pathogens as defined by the GLASS [12]. These networks include the European Antimicrobial Resistance Surveillance Network (EARS-Net) [55] and Central Asia and Eastern Europe (CAESAR) [56], Latin American (Red Latino americana de Vigilancia de la Resistencia a Los Antimicrobianos, ReLAVRA) [7, 57].

- GLASS enables standardisation of data and reporting of AMR data
- Harmonize global standards to produce timely and comprehensive data
- Systems already include LMICs with examples

The main barriers are the lack of standardization of data management, the lack of quality assessment and accreditation of the sources of data, and the lack of quality checks on data collection, analysis, reporting, and sharing [5, 12–14, 25, 26]. As a result, data is subject to bias such as sampling bias and duplication, which may have limited representativeness [12]

- Barriers are lack of standardisation
- Data management and quality
- Bias in sampling which may have limited representativeness

In LMICs, the challenges are high [5, 6, 12–14, 25, 26]. Inadequate health systems governance, absence of health system information, lack of laboratory capacity and infrastructure[50], limited government engagement, loose rules and regulations, lack of resources, and limited staff with adequate experience, expertise, training, and experience in the field are additional limitations [5, 6, 12–14, 25, 26]. Te scarcity of financial resources and reliance on funding to strengthen laboratory capacity is an additional problem because these investments are usually shortterm [13, 14]. This issue can influence the sustainability of the progress made if the government did not implement internal funding and health prioritization plan to strengthen the health systems tackle resistance [13, 14]. Other challenges include supply shortages, supply chain issues, counterfeit products, environmental challenges, diverse socio-economic drivers of resistance, and absence of leadership [26]

- Inadequate health governance
- Lack of laboratory capacity
- Lack of resources
- Inadequate trained staff with expertise and experience
- Reliance on external funding

the other CIPARS farm AMU programs. CIPARS has traditionally worked with veterinarians because of their comprehensive understanding of the AMU on their client farms (improving data quality), existing veterinary-client-patient relationships, and their ability to ensure confidentiality. While producers have access to individual animal or group data, veterinarians are responsible for providing AMU oversight. If required, veterinarians could be compelled (through professional and/or government regulation) to report AMU as part of maintaining licensure, whereas there are no similar incentives to encourage producers to disclose AMU data. Compensation for the extensive time and effort currently needed to collect and report AMU information is important as these data are often sought for public good. If producers were the main target for data collection, much thought and planning would be required to keep them engaged, maintain data quality, demonstrate value, and compensate them for their time.

- Improving data quality
- Compensation for time and effort for AMU information

Challenges in data acquisition and organization arise when surveillance is not the intended purpose of the data (41). In the Canadian feedlot cattle industry, individual animal data recording systems are designed to be a version of computerized medical records and include direct recording of all individualadministered product use including AMDs. Summarizing these data is relatively straightforward because the collection/storage systems are designed for AMU data input and retrieval. However, in-feed AMU data summarization is not as straightforward because current feed-related data collection systems are designed to collect feed data not product data such as AMDs. For successful, accurate and efficient data acquisition, collectors must be able to access and assemble clearly defined/required pieces of information prospectively and easily. This does not mean that all raw data must come from the same data collection system(s), but they must be easily accessible. Furthermore, a standardized AMU storage system with mandatory fields and controlled data formats must exist for compilation, storage and timely summarization of data. The challenge is then to identify who bears the associated cost and responsibility to create, support and maintain the required infrastructure

- Data acquisition is difficult
- Summarizing data is relatively straightforward
- Feed-related data designed for feed-data and not product data
- Standardised AMU storage system with mandatory fields must exist

It is expected that veterinary management groups will continue to optimize data collection through software development, targeted data mining strategies, and a priori collaborations with feedlot management to streamline AMU data collection critical control points such as ration naming, composition recording, and access to feed data. As well, development continues on the integrated database that receives, stores and maintains the CanFASP AMU surveillance data to improve system efficiency and the speed at which analyses and results can be compiled and distributed to industry stakeholders, veterinary management groups and producers.

- Optimization data collection
- Improve system efficiency of analyses
- Faster results compilation

CanFASP has funding for three years from 12 different sources (Table 1), including the feedlot industry, pharma, retail, federal, and provincial governments, with extensive voluntary time contributions from government, academia, veterinary practice, feedlot producer and industry stakeholders. However, without dedicated funding and human resource allocation, continuation, maintenance, and upgrading of the system will be a challenge.

- Funding sources
- Dedicated funding needed or improving system will be a challenge

Regardless of the computerized data collection system used at each feedlot, it is key that: (1) feedlot team members are appropriately trained and engaged to capture all relevant data points in real-time, and (2) the computerized data collection system is sufficiently user-friendly to ensure data entered at the feedlot are as accurate and complete as possible. Once data have been entered, veterinarians or veterinary management group team members compile data for upload to surveillance system .Canadian Feedlot Cattle Antimicrobial Use administrators. It is critical that the database into which the raw data are entered can be specifically mined to allow relevant data points to be searched, identified and efficiently compiled into a standardized format for reporting

- Feedlot members are trained to capture data
- Platforms are sufficiently user friendly to facilitate that
- Accuracy in data

Individual-animal data collected must provide sufficient detail for required AMU calculations. Identifying in-feed antimicrobials is relatively simple (with only oxytetracycline, chlortetracycline, and tylosin phosphate commonly administered in feedlot production). However, for group-level in-feed AMU data, accessing accurate data is complicated and time consuming as described above.

- Sufficient data is to be provided
- Accessing accurate data is complicated
- Accessing accurate data is complicated

Once compiled, data should be uploaded into a centralized surveillance program database in a formatted and standardized manner so that the administration team members can easily oversee the incorporation of uploaded data. There are several ways to ensure standardization, including enforcing case definitions, using protected software to ensure data entry mistakes are not made, and promoting excellent communication between surveillance system administrators and those compiling AMU data during the upload phase.

- Centralised data platform for processing
- Standardisation of data
- Data entry mistakes
- Standardisation of case definitions

Currently, the international community lacks consensus as to the most appropriate indicator to describe AMU, which depends on the intended objectives of the surveillance system.

- Lack of consensus to appropriate AMU indicators
- Dependent on the objectives of surveillance

The best approach to convey key messages to stakeholders for both AMU and AMR data has not yet been clearly identified. The AMU data are complicated to explain, and often require an in-depth understanding of the topic to comprehend important nuances in calculations and indicators. Without a way to share results so that the appropriate stakeholders can meaningfully act on the findings, the power and purpose of the surveillance system is diminished. It is unlikely that there is a single approach to reporting AMU data that will be useful to all stakeholders that desire these data. For the CanFASP, an annual aggregated summary report will be prepared, as well as reports to individual feedlots comparing their data to national indices.

- Dissemination of information to stakeholders has no centralised approach
- Convey the nuances
- Sharing of results is important

Lim et al. 2021

The mainstay to strengthen the laboratory capacity is government engagement. Health is a political decision [16]. The WHO [174] constitution principles state that "Governments have a responsibility for the health of their peoples which can be fulfilled only by the provision of adequate health and social measures" and "The enjoyment of the highest attainable standard of health is one of the fundamental rights of every human being without distinction of race, religion, political belief, economic or social condition". When the government prioritizes health and the community wellbeing and aims for building a better country for its future generation, one of the main goals is to tackle AMR. Evidence-based data show the detrimental impact on health and economy in LMICs that bear the highest burden of communicable diseases [1]. Te government plays a major role in health systems governance and collaborates with multiple national and international stakeholders to set health policy rules and regulations like antibiotic use and the problem of counterfeit products in addition to secure a reliable supply of other medicines and technologies depending on the country needs [22, 23]. The government must set a national multi-sectoral health plan of the human, animal, and agriculture interfaces and establish evidence-based policies to prevent and treat diseases [172, 173]. The government has the responsibility to conduct gap analysis in the field for a better understanding of the country AMR context, drivers, challenges, and trends and prioritization of expenditures on programs to tackle resistance and for building health systems information and laboratory capacity [22, 23]. Te government assesses the health workforce for health coverage [175] and to fulfill the engagement toward achieving pre-set sustainable goals [176]. Other important decisions include setting a surveillance focal point and a national coordinating center (NCC) that enhances the national to international collaboration [177]. Te NCC can enhance communication and collaboration between the prescribers and the local and national laboratories generated data by highlighting and addressing the gaps and contributory factors to the lack of trust [177]. Reaching these goals means less empirical treatment, less use of broad-spectrum antibiotics, and better disease management, better patient health, and antibiotic use [176]. To enhance AMR awareness, government collaboration with different national and international stakeholders can plan education and training on antimicrobial susceptibility testing standards and data management across different disciplines [177].

- Call upon government engagement to improve laboratory capacity
- Priorisation of health by governments as a mechanism
- Procurement of materials
- Procurement of materials
- Government has responsibility for gap analysis
- Look at gaps in laboratory capacity and staffing
- Increase AMR awareness through collaboration

Investing in education and high-quality research will bridge the lack of sustainability gap.

- Education to address sustainability gap

Laboratories should operate according to national rules and regulations but to standard operating procedures that should be revised and updated regularly. Staf must be aware, educated, and offered periodic training to make sure the same process is applied in each shift to secure laboratory results [13–15, 26, 177]. Securing the supply chain and investing in adapted equipment, diagnostics, and reagents adapted to the environmental, logistic, financial challenges of LMICs is crucial [26]. Locally manufactured diagnostics must comply with standardized norms that secure the quality and efficacy of these products [25, 26].

- Standardisation of laboratory techniques
- Staff need to be trained
- Supply chain must be robust and adapted

Factors that can infuence the quality of data are the methodology of data collection and the adequacy of data interpretation and analysis [55]. Other challenges include the use of unifed internationally accepted techniques and clinical breakpoints guidelines, considered crucial for the interpretation of antibiotic susceptibility testing (AST) results [179]. Te limitations of conducting comprehensive population surveillance on AMR are high in LMICs [5, 13, 15, 26]. Some countries may report data from national surveillance systems with broad population coverage, and others may report data from a subset of local laboratories, clinics, and healthcare settings focusing on one area and limiting the representativeness of data on the national level [55]. As the validity of surveillance systems relies on the comparability of participating laboratories [180], each may have diferent trends of AMR surveillance and diferent level of capacity for identifying the microorganisms and may show diferences in the applied methodology and quality assurance limiting benchmarking [55, 181, 182]. Other inconsistencies across participating laboratories include diferences in sampling, the use of diferent clinical case defnitions, and the heterogeneous healthcare utilization [181]. Standardization of data management is the most challenging task if health information technology and adequate training are lacking.

- Quality of data collection and interpretation
- Internationally accepted guidelines needed
- Inconsistent reporting of AMR data
- Comparability of participating laboratories
- Limited QA benchmarking

At the global level, GLASS published protocols to standardize AMR surveillance data processing. In general, it is recommended that repeated isolates from individual patients are to be removed before estimating prevalence and incidence of AMR infections (deduplication), and data should be stratified by infection origin. Increasingly, open-access applications such as WHONET [55] and AMASS [56] are available to support local sentinel sites, where there is a lack of time and expertise, to process AMR surveillance data and to generate reports. It is acknowledged that there will be challenges in integrating these applications into routine practice of microbiology data collection and into the existing LIS at a local sentinel site. Innovative methods of processing and reporting AST profiles, including WISCA, have been shown to be useful but are so far not commonly applied in LMICs. Frameworks such as Microbiology Investigation Criteria for Reporting Objectively (MICRO) are useful to enhance reporting of microbiology data and comparability of AMR surveillance data, as well as providing some quality assurance [29]

- GLASS protocols to standardise AMR data processing
- Open access applications such as WHONET and AMASS to generate reports
- Frameworks has enhanced data comparability and data with quality assurance

At the national and global level, population catchment and pattern of healthcare utilization in the population are ideally to be included in AMR surveillance reports to interpret AMR surveillance data. However, such statistics are not commonly available in LMICs. The use of data from existing Health and Demographic Surveillance Sites (HDSS) to combine community- and hospital-based clinical and microbiology data has been recommended [8,57] but has not been sufficiently used in LMICs

- Population catchment and pattern of healthcare utilization not present
- Statistics exist but not integrated.

A paradigm shift in the value of microbiological testing and prioritization of the detection of drug-resistant infections is needed in many LMICs [9]. Innovative methods to improve quality and reduce the costs of microbiological testing are needed. Incentives such as patient health insurance coverage and hospital reimbursement coverage to support the costs of microbiology testing may support this shift and motivate the use of microbiology testing in LMIC settings. A healthy and dedicated liaison between the qualified microbiology laboratory and clinicians is also crucial to increase mutual trust and to optimize the use of existing diagnostic tools [62e64]. Moreover, a clear potential advancement in career pathways for the qualified microbiologist and laboratory technicians could further enhance trust and uptake of microbiology results

- Methods to improve quality and reduce microbiological testing are needed
- Shifts in reducing microbiological costs needed
- Liasion needed to increase mutual trust and to optimize the use tools

While the use of microbiology in many LMICs is gradually increasing, essential parameters such as blood culture use rate and

- Standardise measures for data reporting

Hazim et al. 2018

Surveillance readiness assessments revealed that, despite clinical microbiology being conducted, improved laboratory capacity was needed to ensure the available data were consistently interpretable and useful in a surveillance context. In general, assessment findings identified the following gaps with the potential to affect data use:

A lack of standardized procedures for quality assurance and quality control practice; Limited laboratory staff training and experience in microbiology; Limited access to reliable reagents and essential microbiology supplies and equipment; and Inefficient data management and reporting

- Barriers in capacity
- Lack of standardisation
- Inefficient data management and reporting
- Limited laboratory staff training
- Limited access to supplies

Following the identification of phase 1 sites, repeat site visits were conducted to specify laboratory deficiencies, determine resources available, and collaborate with laboratory staff to establish a 1-year capacity building work plan for each site, including the NRL. The hallmark of all these work plans was the establishment of individual laboratory mentorship led by ASM. While costly, direct 1-on-1 mentorship provided the individualized guidance required to address the many types of issues made evident by the readiness assessments. Mentorship helped identify and leverage individual site strengths and promote creative ad hoc solutions to challenging issues. Lastly, in place of a rigid set of requirements for participation, site mentors were able to recommend sites for inclusion based on ongoing process improvement resulting in more rapid surveillance scale-up.

- Repeat site visits to specify deficiencies
- Mentorship and guidance
- Mentorship promoted creative solutions to issues
- Improvement resulted in rapid surveillance scale up

All mentored sites were enrolled in an internationally recognized external quality assurance (EQA) program to help monitor and quantify improved capabilities over time. In addition, sites were provided with relevant quality control (QC) strains as needed and licensed Clinical and Laboratory Standards Institute (CLSI) guidelines for results interpretation and reporting

- External quality assurance enrollment
- Sites provided with quality control strains
- Standardisation with CLSI guidelines

The short-term objective of laboratory capacity building was to move enrolled sites toward providing reliable surveillance data, with an ultimate goal of achieving international accreditation in microbiology. As of June 2018, the NRL had attained international ISO 15189;2012 accreditation, and 1 additional phase 1 site was preparing to be assessed for accreditation. To help ensure laboratory quality improvement efforts are sustained, EPHI-NRL has been gradually capacitated to take leadership of the laboratory mentorship activities. EPHI-NRL staff accompany and support the ASM staff during quarterly mentorship visits and have begun to conduct independent interim site visits to meet this objective.

- Laboratory capacity focused on providing reliable surveillance data
- National reference laboratory capacited to take leadership of laboratory mentorship

The lack of a full complement of reagents, standard microbiology supplies, and the equipment required for culture, identification, and antibiotic susceptibility testing was a critical limiting factor at all sentinel sites. Due to the need to ensure timely availability of essential supplies and equipment for rapid surveillance scale-up, the working group created a list of essential bacteriology reagents and supplies and obtained a 6-month to 1-year supply for each sentinel site from established international sources. For long-term sustainability, this essential list was used to negotiate with the central procurement agency of Ethiopia to help ensure uninterrupted availability moving forward. Laboratory equipment was assessed at each site and was replaced or repaired as needed. Additionally, a system to capture and document issues pertaining to the quality and availability of supplies was established to improve communication between laboratories and allow more coordinated response to supply issue

- Working group created list of essential reagents
- Obtained long term supply of materials
- Laboratory equipment was assessed at each site

A combined surveillance introduction for key clinicians and training workshop on microbiology clinical specimen collection best practices was developed. The training emphasized the importance of appropriate sample collection for use by healthcare facilities and the national AMR surveillance initiative. For long-term sustainability, training materials and job aids supporting the ongoing training of frontline clinical staff were developed. An annual training of trainers program targeting facility-level training staff was established to provide guidance in using the developed materials and to promote effective methods of adult learning

- Training workshop for clinicians regarding microbiological clinical specimen collection
- Annual training of trainers program targeting facility level staff implemented

The national bacteriology reference laboratory at EPHI needed additional assistance to fulfill their vision of being a national coordinating center for AMR surveillance. EPHI participated in early efforts to train and redistribute staff, sought laboratory mentorship, established formal and informal lines of communication with surveillance sites, and improved the supply systems within the AMR surveillance sentinel network. Their efforts were ultimately successful in preparing the EPHI-NRL to fulfill its role as the national coordinating center for AMR surveillance. To ensure long-term sustainability of the AMR surveillance and laboratory mentorship programs, the NRL is continuing to advocate for dedicated staff and resources from EPHI and ministry of health leadership.

- National reference laboratory needed assistance to be a coordinating centre
- Successful in developing a national reference laboratory
- EPHI trained and redistribute staff, sought laboratory mentorship
- Lines of communication were established between surveillance sites

Due to a lack of established procedures and the logistics required for specimen and isolate transport, bacteriology at EPHI-NRL had limited opportunity to engage in reference (providing testing for unknown isolates sent from limited capacity laboratories) or confirmation testing (retesting isolates for results comparison). An innovative but limited public-private partnership between EPHI and the Ethiopian postal service had successfully implemented specimen referral and transport of HIV and TB isolates within a nationwide laboratory network.

- No established procedures for specimen transport
- Innovative partnership created to transport specimen

Evaluations of the laboratory data flow at each sentinel site revealed varied paper-based data management with no consistent methodology for AMR surveillance data capture. Small pilot projects exploring the integration of WHONET software for surveillance site data entry were initially challenging because of the perceived disruption to existing workflow and increased workload. Ultimately, individualized solutions for data entry were created to ensure data collection was integrated with existing practices and was acceptable to laboratory staff and management

- Paper based data management was inconsistent for AMR data capture
- WHONET software for data integration trialed but challenging
- Individual solutions for data entry integrated with existing practices and laboratory staff and management

rates of AMR stratified by antibiotic usage should also be used to standardize the measures of prevalence and incidence, and increase the comparability of AMR burden across space and time [25]

Investments in improving microbiology quality and use through expensive automated equipment do not always deliver, as operating costs and costs of maintenance and consumables have not been taken into account. Maintaining power supply and cold chain often remains challenging. Innovative tools to simplify microbiological diagnosis [65] and to conserve diagnostic reagents will be useful to fill the AMR data gap from LMIC settings

- Investments needed to improve microbiological quality
- Maintaining power supply chain and cold chain remains challenging

Most importantly, financial support is needed to build the local capacity, including establishing surveillance teams and increasing availability of diagnostic tools, in order to ensure a sustainable case-based surveillance system. Increasingly, different international organizations have devoted efforts to strengthening AMR surveillance in LMICs. An example is the Fleming Fund (UK government), which supports 24 LMICs in Africa and Asia [66].

- Financial support needed to build local capacity
- Examples included Fleming fund

Quality assurance and control (QA/QC) are essential to ensure accurate identification of pathogens and AST profiles, and need to be improved in sentinel surveillance sites in LMICs. Commercial QA/QC schemes and accreditation programmes are available, but often unaffordable for laboratories in LMICs. Training and support to ensure quality performance and methods of bacterial identification and AST are needed for laboratories in LMICs [67]. Web-based tools such as the laboratory quality stepwise implementation tool and other quality management-strengthening programmes, are useful to guide laboratories, especially those in LMIC settings, towards implementing quality management systems [68e70]. In addition, prioritizing the microbiology testing in the local setting, increasing the number of microbiology testing facilities, and strengthening the local capacity of the existing laboratory system are needed

- Quality assurance essential
- Training and support needed to quality performance
- Quality management strengthening programmes
- Increasing number of microbiology testing facilities, strengthening local capacity and existing laboratory systems

Verifying AST results of bacterial isolates is an important component of surveillance to ensure quality data are generated. However, data verification and highlighting isolates with unusual AST results can be a complicated and time-consuming process to perform manually. Commercial laboratory information management systems with functions to support microbiology data verification are unaffordable for many hospitals in LMICs. The open access WHONET programme has functions to support data quality checks [55]. Open-access, offline, and user-friendly tools that can automatically process and analyse microbiology data can be useful in resource-limited settings in generating AMR surveillance reports for local use and sharing in a timely fashion

- Data verification and highlighting isolates is complicated
- Information systems support microbiological data verification unaffordable
- WHONET has data quality checks

Finally, there is a lack of evaluation frameworks for continuous AMR surveillance systems in LMICs. Regular evaluations on the performance of AMR surveillance systems in LMICs would be useful to identify limitations and areas for improvement as well as to quantify and qualify progress when it occurs. Examples of evaluation tools for AMR surveillance network are the AMR Progressive Management Pathway tool developed by the Food and Agriculture Organization of the United Nations, NEOH and SURVTOOLS [71]. The implementation of such tools in LMIC settings is uncommon

- Lack of evaluative frameworks for systems
- Regular evaluations on the performance in LMICs
- Examples: management pathway by FAO

Mader et al. 2021a

However, limitations in human resources at ANSES combined with a decreasing budget over the years have prevented the integration of additional laboratories, significantly impacted communication and laboratory training activities, and reduced the proportion of samples subjected to molecular analyses. To enable these developments, an increase in financial and human resources in the coordination team was recommended

- Limitations in human resaources
- Limitations due to budget
- Barrier to integration of additional laboratories
- Financial and human resources in coordination team recommended

In the end, the coordination team receives a great diversity of file formats and had to develop a series of semi-automated solutions to convert laboratory files into the appropriate format to reduce the workload of data cleaning and harmonisation. In addition, most laboratories do not send their data every three months, leading to heavier workloads during some periods. This situation led to a sub-optimal score for data collection (Fig. 2) and timeliness (Fig. 3). However, all laboratories send their data at least once a year, enabling the analysis of the complete RESAPATH database each year. Another weakness is the existence of two RESAPATH databases in two different ANSES laboratories which are not integrated: one in Ploufragan-Plouzané-Niort with data from swine, chickens, turkeys, rabbits and fish and another one in Lyon with data from other animal species, mainly ruminants, equids and companion animals.

- Coordination team developed standardised data harmonisation
- Heavy laboratory workload leads to inconsistent data collection
- Weakness with unintegrated databases

Its capacity to conduct multi-disciplinary analyses due to skills in bacteriology, epidemiology, biostatistics and IT within the coordination team was considered a strong asset

- Capacity to conduct surveillance in the multi-discplinary analyses

From this evaluation, key success factors for RESAPATH were identified and included:
• A strong central institutional organisation with the inclusion of many relevant actors in the animal sector defining clear surveillance objectives, scope and procedures that meet their expectations.
• Strong skills in epidemiology and microbiology at the central level, including the capacity to complement phenotypical surveillance with molecular surveillance.
• A collaborative win–win approach between member laboratories and the coordination team, leading to the voluntary participation of numerous field laboratories throughout the whole country.
• The provision of free technical support to laboratories including an annual PT enabling the production of high-quality and harmonised AST data.
• Strong internal and external communication with all possible end-users of surveillance data, including in the human health sector.
• Continuous efforts to assess its performance due to laboratory PT, performance indicators and OASIS evaluations, followed by the implementation of training and improvement measures.

- Strong central institution helps define scope and procedures
- Strong staff skills helps capacity
- Collaborative approach
- Free technical support to enable harmonized data
- Strong internal and external communication
- Continuous for assessment

. The most important one referred to data management and a lesson to learn would be to always consider scalability, a parameter that is often overlooked at the setup of a system. Taking into account the limited IT capacities of laboratories is key to maintaining a strong volunteer network, but this requires a lot of flexibility from the coordination team and can be very [illegible]

- Data management need to be considered for scalability
- Limited IT capacities of laboratories

Developing a common vision for AMR surveillance allowed public health leaders throughout the Ethiopian public health system to constructively discuss and debate current limitations in laboratory infrastructure, microbiology supply procurement, laboratory data quality, and the far-reaching implications of the lack of information technology and digital laboratory information management systems. Through the implementation of AMR surveillance, GHSA provided access to the funding and the technical expertise needed to sustainably address some of these issues, thus building public health capacity through the performance of core public health functions. Following the successful initiation of AMR surveillance, the Ethiopian AMR working group identified several potential areas for growth: Expansion of surveillance activities to additional sentinel sites Establishment of bacterial isolate and specimen transport between peripheral laboratories and the reference laboratory at EPHI Exploration of alternative AMR surveillance strategies, including an isolate-based referral system in which participating laboratories submit presumptively identified isolates for confirmation of culture identification and antibiotic susceptibility testing; and/or time-limited surveillance, where laboratory capacity is temporarily augmented to provide reliable quantification of resistance over relatively short time periods

- Current limitations in the system addressed by developing a common vision for AMR surveillance
- Expansion of surveillance through sentinel sites
- Transport mechanisms to be established
- Standardisation of procedures and criteria

Kakooza et al.

The establishment of Kampala EGASP demonstrated the feasibility of implementing the WHO EGASP protocol in a resource-limited setting,1 through collaboration and partnerships between government, WHO and academic part

- Establishment of EGASP shows success in resource limited setting
- Partnerships between government, WHO and academia

In Kampala EGASP, the surveillance is systematic and based on WHO standardised laboratory methodologies and surveillance protocols,12 13 within the largest city, which is a strategic sentinel site for emerging NG AMR given national and international travelling and the frequency and poorly regulated access to empiric antimicrobial treatment

- WHO standardised laboratory methodology and protocols

Furthermore, the Kampala EGASP being led by the National STI Control Programme at MoH presents an opportunity of a long-term sustainability since the surveillance activities have been integrated into the daily routine patient care at the health fa

- EGASP lead by MoH
- MoH leads to sustainability of surveillance activities

The use of the WHONET system for data collection, collation and analysis has also bridged gaps in data management while achieving WHO targets for AMR surveillance.1

- WHONET has assisted in data collection, collation, and analysis

The main limitations of the present surveillance included that only male urethral samples were collected and no samples from other anatomical sites and/or from women, relatively low culture positivity rate of Gram-stained positive samples due to for example long transport times and sub-optimal sampling swabs in some time periods, lack of recommended WHO NG reference strains for quality assurance and control (will be included in future surveillance)7 9 24 and lack of follow-up of participants to determine treatment outcomes.

- Only male urethral samples
- Low positivity rate due to transport times and sub-optimal swabs
- Lack of quality assurance and control

Through public–private partnerships, we show the feasibility of setting up a structured, standardised and quality-assured EGASP based on a WHO protocols in a resource-limited setting

- Public and private partnership
- Facilitated standardised and quality assured

Kariuki et al. 2018

These challenges range from inadequate resources and weak supply chains for consumables for microbiological laboratory procedures, lack of human resources well trained in AMR surveillance and poor or lack of commitment by facility management to embrace AMR as a healthcare issue. Where facilities do exist, under-utilisation is common. However, despite the lack of infrastructure and other resources required to perform optimal surveillance for AMR, most laboratories in Africa can still generate, collate and disseminate quality data, albeit with some ingenuity. If there is a lack of samples for systematic evaluation of AMR trends, mutually beneficial partnerships between centres responsible for coordinating surveillance and local hospitals or healthcare facilities can jumpstart focused surveillance on specific organisms. Such centres can then gradually expand the surveillance to include more pathogens and more facilities

- Challenges in surveillance
- Inadequate resources and weak supply chains
- Training in AMR surveillance and lack of commitment by facility management
- Lack of infrastructure
- Mutually beneficial partnerships between centres

Indeed, we could perform systematic sentinel surveillance so as to generate reliable, accurate and quality-controlled data, using the minimal resources typically available in our settings, for implementation of infection control and prevention programmes.

- Sentinel surveillance to generate reliable accurate data

For quality assurance, which is a hallmark of a standard surveillance system, it will be important for laboratories to be registered and participate in an external quality assessment scheme operating to internationally recognised standards, some of which are supported by WHO and other partners and are therefore potentially more affordable for laboratories in our African setting

- Quality assurance is important for laboratories
- International recognised standards

On funding AMR surveillance activities and for a sustainable AMR surveillance programme, it will be critical that budget commitments made by national governments to support the national action plan activities are adequately maintained. This too will require some ingenuity so as to demonstrate that data from AMR surveillance actually impact on delivery of

- Funding of AMR surveillance requires budget commitments
- Work with health economists

a lot of flexibility from the coordination team and can be very time-consuming

Another weakness of RESAPATH lies in its possible sampling biases, as a passive laboratory-based surveillance network that does not integrate the sampling stage in its procedures. However, these possible biases were not considered as having a major impact on representativeness in this evaluation. On the other hand, the current organisation of RESAPATH brings a lot of simplicity, which is key to its sustainability.

- Sampling bias
- Representativeness of this surveillance system

This detailed investigation of the strengths and limitations of RESAPATH provides valuable information to countries aiming to set up a national AMR surveillance system in diseased animals using a passive surveillance approach or to those wishing to improve their current system. It shows how participative win– win surveillance networks are of strong value for national authorities and can succeed in triggering highly positive collateral impacts, such as capacity building in veterinary diagnostics through greater skills acquired by all members of the network.

- Shows participative approach can succeed

Mader et al. 2021b

Whereas most AMR data in the human sector originate from diseased individuals, the existing surveillance systems lack AMR data from clinical isolates of animals. They are therefore of limited help to assist veterinary practitioners in antibiotic choice and policymakers in regulating veterinary antibiotic use, towards the shared goal of reducing AMR while ensuring optimal treatment of animal infections.

- Lack AMR data from clinical isolates
- Limited in the use to veterinary practice

Some programmes (VetPath, ComPath and MycoPath), managed by the European Animal Health Study Centre (CEESA, Centre Européen d'Etudes pour la Santé Animale in French) on behalf of a consortium of pharmaceutical companies, produce harmonised AMR data in diseased food-producing and companion animals across EU countries [8], but they cannot replace a continuous European surveillance system for AMR in veterinary medicine

- Programs on behalf of a consortium produce harmonised AMR data
- Cannot replace continuous surveillance

However, these systems are fragmented; do not always monitor the same animal species, bacterial species and antimicrobials; and do not always use the same antimicrobial susceptibility testing (AST) methodologies and interpretative criteria. On the other hand, some European countries do not have a surveillance system for AMR in diseased animals in place, even though research-based information may be provided occasionally.

- Systems are fragmented in data capture
- Standards are not the same

The number of European countries implementing an AMR surveillance system in veterinary medicine is expected to rise and some countries (e.g. Spain) are currently developing surveillance systems, while other countries' national action plans (e.g. Italy or Belgium) highlight the need and willingness to improve AMR surveillance in this domain. These initiatives present an opportunity to launch coordinated surveillance at the European level and to develop a common surveillance framework to address the lack of harmonisation between national surveillance systems.

- Need to improve and launch coordinated surveillance
- Address harmonisation at national level

It is in line with Regulation (EU) 2016/429 on transmissible animal diseases ('Animal Health Law'), which states that "effective collection and management of surveillance data should be established at Union level […] when relevant, for emerging diseases or antimicrobial-resistant pathogens" [19], and Regulation (EU) 2019/6 on veterinary medicinal products, which points out the need for AMR data [20]

- Animal health law points out need for AMR data

EARS-Vet would provide the necessary coordination to collectively define common microbiological and epidemiological standards for the surveillance of AMR in animal bacterial pathogens in Europe, as well as a strategy to reach effective harmonisation. More broadly, EARSVet would represent an opportunity to build a European scientific community and knowledge hub to support the establishment, improvement and harmonisation of respective national surveillance systems, the interpretation of surveillance outputs and their translation into interventions. This EARS-Vet community could also prove useful in urgent contexts; for example, when an emerging resistance mechanism is discovered and its spread across Europe needs to be quickly evaluated, as experienced in 2015 upon the discovery of plasmidmediated colistin resistance [23].

- EARS-Vet provides standardisation
- Standardisation of interpretation of surveillance outputs

To reach harmonisation, we suggest that standards are defined in an inclusive and bottom-up approach, i.e. according to what is considered relevant and feasible within countries. In the beginning, we envisage a transition period where different AST standards would be accepted, as originally done by EARS-Net over two decades, before accepting only those methods complying with the European Committee on Antimicrobial Susceptibility Testing (EUCAST)

- Harmonisation when standards are defined in a bottom up approach
- Transition period when moving to EUCAST

The possible sampling biases are linked to the fact that national surveillance systems usually collect AST results routinely produced in veterinary diagnostic laboratories. As culture of veterinary diagnostic specimens is often not performed until treatment failure, AMR levels tend to be overestimated compared with first-time infections [24]. Such biased estimates can have important consequences, including wrongly recommending the use of critically important antimicrobials as first-line treatments when other antimicrobials will actually be effective in the majority of cases. In order to ensure comparability of AMR data between countries, the representativeness of AMR data would need to be assessed before results are interpreted. As in EARS-Net, a series of indicators of national geographic coverage and representativeness could be defined and regularly calculated to understand the validity of the surveillance data. In addition, pragmatic solutions should be explored collectively to address sampling biases. Of note, some countries have decided to subsidise ASTs (e.g. Czech Republic and Spain) to collect more representative AMR data comprising a broader range of cases, i.e. not only those after treatment failure

- Sampling biases linked to national surveillancew
- AMR levels can be overestimated
- Comparability of data, representativeness needs to be assessed
- Indicators for geographical coverage and understanding need to be established

Several successful initiatives, such as EARS-Net and ESVAC, have proven that a European surveillance network can be developed gradually, starting with a group of countries collaborating despite initial lack of harmonisation. Long-term success of EARS-Vet would depend on political commitment at national and European levels, accompanied by sustainable

- Success examples with collaboration depsite harmonisation

healthcare, food security and sustenance of a healthy environment. Thus, governments are constantly reminded that good surveillance systems are actually cost efficient, particularly in the long term. We need to work with health economists to deliver data on those economic costs associated with a lack of action on the AMR menace in our countries in a language that policymakers will easily understand

- Commitments from governments
- Governments reminded surveillance is cost effective

In the meantime, we will need to utilise the resources currently available through various initiatives, such as the tripartite United Nations agencies (WHO, the Food and Agriculture Organization and the World Organization for Animal Health), as well as other partners who have specific interests in promoting and supporting One Health AMR surveillance in Africa. The outlines of two successful routes to national surveillance, Ibrahim et al.17 and Opintan18, showcase how donor funds were leveraged to support local planning and initiatives in Ethiopia and Ghana. In making the most of external initiatives and opportunities, however, we must remember that these will only serve as short- and medium-term solutions – we have to own the process of fully implementing our national action plans, using our own resources, if AMR surveillance and containment will be sustainable, and serve our principal national goals and objectives.

- Utilise current funding through United Nations agencies
- Utilise partners
- Donor funds to support local planning and initatives
- Short term funding and the need for long term sustainability

Kaur et al. 2019

Although used worldwide, some of the limitations of collectors include issues with data security, integrity, confidentiality and corruption in shared computational resource settings, limited client-server architecture, platform dependence such as limitation in installation and data entry from other platforms, and lack of data validation. Some limitations of integrators include closed source with constrained capability for adding new modules, lack of data validation, limited client server architecture along with restricted ability to enforce uniform standards for data collection, and analysis across the network

- Limitations include data security, integrity, confidentiality, corruption in shared computation resource settings
- Limitations include data security, integrity, confidentiality, corruption in shared computation resource settings

To overcome the limitations, a hybrid solution (Figure) that can bridge individual laboratories and hospitals to complete picture of AMR in the country would be required. Some of the desirable qualities of the hybrid AMR surveillance tool are use of latest technologies, freely available in the public domain, standardization in data collection and analysis, customization, scalability and extensibility, built-in tools for real-time data analysis, and import and export AMST data in standardized data exchange format.

- Hybrid solution in partnership with laboratories and hospitals
- Standardise data exchange formats
- Use latest technologies
- Standardise data collection, analysis, customization and scability

One of the mandates of both the programmes included developing an informatics solution which could enforce both quality antimicrobial susceptibility testing in laboratories and provide analytics to support the development of national policies on antimicrobial usage.

- Mandates for developing an informatics solution for quality susceptibility testing and provides analytics

Leger et al. 2011

The success of this national farm-based surveillance program for AMU/AMR depended greatly on extensive consultation and transparency given the contentious nature of these issues in food animal agriculture and their potential public health impacts, and that data provider participation was voluntary. Preliminary consultations with commodity groups and provincial Ministries of Agriculture and Food provided information that led to the decision to pilot this surveillance program in swine. The Canadian swine industry had a mature certified on-farm food safety and quality assurance program (CQA®), and this commodity had not experienced a recent foreign animal disease outbreak, unlike the beef (BSE, 2003) and broiler poultry (Avian Influenza, 2004) sectors. Also the United States, a major trading partner in this commodity, had launched a similar surveillance system in swine (6)

- Success depended on extensive consultation
- Commodity groups and ministries which led to pilot surveillance
- Assurance program.

The engagement of a commodity-specific ERP and AC ensured the surveillance infrastructure would be practical, efficient and effective. Involvement of national and provincial industry organizations and government agencies in the development phase of this surveillance initiative ensured timely and transparent communications to constituents. Major concerns for industry were related to time management, farm biosecurity, data confidentiality and the dissemination of surveillance findings

- Engagement with commodity specific ERP
- Collaboration of industry and government ensured communication
- Industry, data confidentiality and reporting surveillance findings

There is no legislated mechanism in Canada that facilitates the collection of antimicrobial usage surveillance data from the pharmaceutical industry, feed and farm supply retailers, veterinarians or producers (2), although since 2006 the Canadian Animal Health Institute has been voluntarily providing veterinary antimicrobial distribution data aggregated to antimicrobial drug class (3). Given the volunteer nature of this surveillance system, members of the AC recommended that producers and veterinarians be compensated for the time required to complete the detailed questionnaire. Situations where payment for data is appropriate and effective are limited (7) but there is evidence for the importance of monetary compensation in sustaining the participation of data providers in this particular surveillance program. Through 2009-10 there were significant declines in hog prices, slaughter volumes and exports (8). In the face of this significant down turn in the Canadian pork industry, the CIPARS Farm program continued with minimal erosion in the number of sentinel sites. The importance of compensation in maintaining ongoing surveillance operations was also supported by comments provided by collaborating veterinarians

- No legislated mechanism for collecting surveillance data
- Volunteer nature of the system
- Payment for data has been effective
- Compensation of maintaining surveillance is needed

The sustainability and relevance of a surveillance system is a function of its methodological and operational flexibility and responsiveness (4, 7). Refinements made during the implementation year were in response to poor questionnaire compliance and data quality. Further refinements were made for the 2009 sampling year based on variance component analysis, which indicated little temporal variation. Sampling protocols changed from two pens sampled per sentinel site three times per year to 6 pens sampled per sentinel site once per year, and revisions were made to further simplify the questionnaire.

- Refinements made to surveillance
- Poor questionnaire and data quality
- Sampling protocols changed with revisions

- Communication plans between AC members [illegible]

funding

- Success dependent on commitment and funding

Mitchell et al. 2020

A participant working within an international organisation spoke of the limited collaboration between the sectors, he mentioned, "we [the organisation] felt there was a lack of intersectoral communication regarding AMR surveillance". (Participant 1, man, human health sector). It was recognised by one senior official that, "the most difficult thing lays in the intersectoral collaboration, at the same moment, focus on the investigation together to give out the result, that is the difficulty" (Participant 5, man, human health sector). One animal health government researcher described the lack of respect the animal sector received, and it was this that caused barriers between collaborations

- Limited collaboration between sectors
- Animal sector not appreciated
- Barrier in collaboration

Twelve of the 17 participants referred to a One Health approach to enabling "a fuller understanding" or a "more complete view" of the AMR situation in Vietnam. One participant spoke of absence of team work in addressing AMR amongst the government sections. Increasing the cross-sectoral training was thought to be a mechanism to increase team work and collaboration and to ultimately build knowledge across the sectors, as one participant from the provincial level mentioned, "we can see different perspectives from different sectors for the overview of current [AMR] situation" (Participant 16, woman, animal health sector).

- One health approach increases complete view of AMR
- Cross-sectoral training to increase collaboration

From the participants perspectives, sharing of information was beneficial; however, some participants also expressed the need for increased resources to enable collaboration and increase sector knowledge. "It's the biggest issue, because in our country we have very limitation on the resources so when we work in the One Health [framework] we can share data, share information and publication our work" (Participant 11, woman, human health sector).

- Need for increases resources to enable collaboration

At the national and provincial government level, perspectives focused on when collaboration was required and limited level of cohesion between investigative methods and analysis. Five of the participants spoke of the need for a One Health approach to be established at every level of an AMR surveillance system to coordinate surveillance protocols, data collection and analysis and share information across sectors. For example, one national government employee from the human health sector spoke of "all must have the intersectoral collaboration. For instance, the problem in the stage of, that is, to evaluate the current situation also needs the intersectoral collaboration. Secondly, in the stage of develop the cooperative solution, there also need the intersectoral collaboration. And after that, it also need [s] the measurement on the impact, the result, and the cooperation of the, the sectors" (Participant 5, man, human health sector).

- Limited cohesive in investigative methods
- Need for intersectoral collaboration

There were differing opinions on the diagnostic capacity of the animal health and human health sectors; including, the human resources available, equipment and laboratory capacity and the available finances. At the provincial level, the participants spoke of the need to send samples to Hanoi for further testing, time constraints and the high financial cost. From a national perspective, participants stated instances of not having the appropriate equipment, adequate human resources or finance to conduct some laboratory tests and that samples are then sent for testing overseas. Within the hospital system, the participant spoke of the limited ability to isolate patients and inadequate financial resources causing difficulty and delays to conduct the most appropriate tests, "the condition in our country Vietnam it, the infrastructure it is not as good as the overseas. Don't know that… there is no ward for isolation, no sanitary ward, that's it." (Participant 12, man, human health sector)

- Diagnostic capacity limited by time and financial costs
- Do not have appropriate equipment
- Infrastructure is limited

Participants from the animal health and human health sectors spoke of the challenges in acquiring sufficient supplies and resources to perform laboratory tests. The challenges included the large amount of paperwork limited supplies and poor equipment. However, in contrast one officer in animal health management noted that it is easy for people in the field to collect the samples and conduct the most appropriate test. She believed that this was similar to the situation when collecting samples for antibiotic residue testing, "[here] we do the coordination… we design then we ask them to do this and that, so they proceed". (Participant 8, woman, animal health sector)

- Challenges in acquiring supplies and resources to perform tests
- Large amounts of paperwork, limited supplies and poor equipment

Poor data quality and insufficient comparable data on AMR in the human and animal health sectors are a key barrier to accurate comparisons between human and livestock populations and between countries and regions [26]. Participants believed that once these are overcome, information sharing across sectors may increase. The challenges of collaboration and information sharing between sectors have been reported elsewhere in Asia, where cooperation may be written in policies but lacking in practice [33]. Information sharing amongst sectors in this study was highly dependent on informal relationships rather than the collaborations based on formal policies. The participants at the provincial level spoke of the limited financial support to undertake interdisciplinary work or collaborate with other sectors due to institutional barriers set by higher management. Limited resources allocated to facilitating cross-sectorial collaboration has been a reported barrier to enabling networks [34]; although, it is essential to support the establishment of networks amongst the different levels of government, non-government and international organisations.

- Data quality is poor and incomparable
- Written in policy by lacking in practice for data sharing
- Limited financial support to undertake collaborative work
- Limited resources to facilitate cross-sectorial collaboration

Government agencies have pledged their commitment through the development of two National Action Plans (one in the animal and one in the human health sector) and convened a National Steering Committee on Antimicrobial Resistance involving actors from across the human and animal health sectors [21, 22]. Yet many of the participants believed there was limited commitment by the Vietnamese government to address AMR. From the perspective of the participants in this study, this lack of uniformity in the government's National Action Plans on antimicrobial resistance and the limited number of meetings between the steering committee signalled a lack of commitment by the government. Without a more unified approach between sectors, it may create difficulties for partnerships and information sharing to develop lower down the hierarchy. This was highlighted by participants working at the provincial level, who continuously mentioned that they need direction and confirmation from National senior management if they are to collaborate with other sectors. However, one participant mentioned involvement in the One Health activities in Thai Nguyen as part of the Vietnam One Health University Network and stated that this created valuable insight and networking opportunities which would not have been possible otherwise. Academic institution level strategies that develop skills in interdisciplinary training may be able to be translated to government agencies. Participatory modelling approaches to improve crosssectoral collaborations have been advocated previously for South East Asia, with provincial governments being a key focus area [35]

- Limited government committment
- Lack of uniformity in AMR plan
- No unified approach to AMR between sectors
- Need direction from national senior management
- Training and academic skill may improve interdisciplinary training

Industry concerns regarding the reporting of surveillance findings were addressed through the development of a communications plan that provides notification to core AC members prior to publication. Oversight of data management and analysis is provided by the WG; other than input from experts on the utility of different parameters in presenting data, industry is not involved in data interpretation or reporting.

- Communication plans between AC members.
- Data management done by working group

Malania et al. 2020

In general, AMR surveillance requires an enabling environment and a commitment to quality of care, which would allow professionals from different areas of the healthcare system to adhere to good clinical practice, to communicatewell, to harmonize practices, and to carry out tasks in a timely and high-quality fashion. This multifaceted or multidisciplinary approach relies on a functional infectious disease diagnostic cycle: clinicians taking and submitting clinical samples to the microbiology laboratory, a bacteriology laboratory able to perform species identification and AST, as well as a system to report, collate, analyse and interpret data to inform those who need to take action [13,14]. With the successful implementation of an AMR surveillance system, the collected standardized and validated data can guide countries in developing empirical treatment guidelines, evidence-based public health policies and interventions. Fig. 1 shows a set of essential elements for developing AMR surveillance. When these building blocks are in place, the system can expand in a stepwise manner

- Harmonize practices
- Requires functional disease diagnostic cycle
- Successful implementation with standardised and validated data

Governmental support

Experiences with implementing AMR surveillance in eight South Asian and Southeast Asian countries have shown that the sustainability of a surveillance system and continued training depends on internal government funding and sustained support from policy-makers [5]. This includes the development of a national action plan (NAP) for AMR as well as funding sources for its implementation [13,15,16], the establishment of a national reference laboratory (NRL) and the forming of a coordination committee with Ministry of Health (MoH) engagement [13,15,16].

- Government support
- Sustainability with continued training
- Sustained funding from policy-makers

Laboratory capacity and quality management

Laboratory capacity is too often the bottleneck for AMR surveillance. Establishing internal quality assurance and participation in external quality assessment (EQA) programmes for laboratories, development and updating of national standard operating procedures (SOPs) to ensure standardization and harmonization of laboratory procedures, as well as continuous training and motivation of staff are all important tools to build this capacity and ensure quality [5,13,15,17,18]. Seale et al. recommend that countries appoint a central coordinating laboratory which could fulfil and/or coordinate the above functions [15]. In addition, using guidelines for surveillance ensures standardization of the selection of specimens, organisms and antibiotics for testing [5,16].

- Laboratory capacity is important
- EQA for laboratories needed
- Harmonization of procedures
- Training and motiviation by staff important to build capacity

Materials and supplies

To meet international standards for AST, bacteriology laboratories require an adequate infrastructure, including reliable equipment, a sustainable supply of quality consumables and appropriate staffing [13,17e19]. LMICs in particular struggle to address specific shortages in supplies of water, electricity and infrastructure to allow laboratories to function properly [5,13]. The successful implementation of a sustainable AMR surveillance programme in Nepal a country with poor access to good-quality reagents, inadequate storage facilities of reagents and frequent power failuredhas shown that encouraging laboratory management in incorporating supplies in their laboratory procurement plan is effective and sustainable [17].

- Adequate infrastructure including reliable equipment sustainable supply of quality consumables
- Successful AMR surveillance requires access to good-quality reagents, inadequate storage, reagents and frequent power
- Successful AMR surveillance requires access to good-quality reagents, inadequate storage, reagents and frequent power

Sample collection, data management and reporting

An effective AMR surveillance programme requires a high level of diagnostic stewardship, including all stages of diagnostic practice, from procedures that guide specimen selection and collection, through processing of the clinical samples in the laboratory, to the reporting and interpretation of results [5,20]. Clinical guidelines should be in place to ensure that the right patients are properly sampled. Implementing laboratory information management systems (LIMS) in LMICs, instead of paper-based data recording, improves AMR surveillance by reducing workload and errors, and enables standardized data reporting to a national collection point [13,19]. The WHONET data management software provides these features and is freely available [21]. However, for the many laboratories without such systems or sufficient IT support, appropriate human resource allocation is required for manual data entry

- Laboratory information systems reduce workload errors and help standardise data reporting
- Appropriate human resources needed with training
- WHONET software allows for standardisation

Experiences from piloting the PoP project in Georgia

Before Georgia joined CAESAR in 2015, clinical microbiology laboratories in hospitals were heavily underutilized (1.8 blood cultures/1000 patient-days), most laboratories were working with low-quality materials for AST, using outdated guidelines, without quality control or confirmatory testing. In the last decade, however, Georgia has made significant progress in reforming the healthcare system. Universal Health Care was introduced in 2013, allowing citizens access to medical services, and the healthcare budget was doubled [11]. Efforts to address AMR were increased, as demonstrated by the development of a National Strategy for Combating AMR according to theWHO's Global Action Plan on AMR [28], and by joining the CAESAR network. In addition, Georgia participated as the first country in the PoP project, which was carried out from July 2015 to December 2016 in four hospitals and led by a team of trained national project coordinators.

- Georgia as an example to improve surveillance through CAESAR network
- Led by trained national project coordinators

Governmental support

The rising threat of AMR and the WHO resolution (WHA68.7) calling for member states to develop a national AMR strategy urged the Georgian government to the following actions.

- WHO resolution calls for participation

Sustainable finance.

As microbiological diagnostics consumables were funded through the CAESAR project for the duration of the PoP project, sustainable funding was needed in order to keep up activities after the project ended. Management of participating hospitals was willing to increase the budget for microbiological diagnostics because the PoP project showed that better targeted treatment, resulting from using microbiological diagnostics, led to cost savings. In addition, new regulations required hospitals to monitor the occurrence of specific infections and laboratories to establish a quality management system (QMS). Also, the government decided to subsidize the NRL for AMR surveillance activities, covering expenses for confirmatory testing.

- Sustainable funding for upkept activities
- Management of participating hospitals increased budgets
- Management of participating hospitals increased budgets

Establishment of an NRL

- National reference laboratory created a support network

Monnet 2000

In this study, the majority of the participants reported that the One Health surveillance system should be developed with all sectors involved from the design stage. The participants perceived a One Heath approach to strengthen their capacity to achieve their own sectors objectives to tackle AMR by gaining new knowledge and understanding. To achieve successful multi-sectorial participation, the development of trust, transparency, equal representation and consensus amongst all relevant sectors is needed [24].

- One Health surveillance should include collaboration
- Consensus across sectors is needed

Inversely, data from clinical microbiology laboratory information systems generally do not include clinical information on the isolates, which makes it difficult to differentiate between infecting and colonizing isolates and between nosocomial and community isolates. However, they are more adapted to the specific objectives of antimicrobial resistance surveillance because they are produced daily by clinical microbiology laboratories without additional workload. These data can easily be extracted from most existing electronic laboratory databases and analyzed with the help of simple software package such as WHONET, that can be obtained free of charge from the World Health Organization (WHO) [4,5]. To standardize and improve local antimicrobial resistance surveillance, the recent WHO guidelines for antimicrobial resistance surveillance recommend a selection of indicator microorganisms and antimicrobial agents to be tested [6]. Additionally, these guidelines present summary protocols for antimicrobial susceptibility testing and emphasize the importance of quality control programmes.

- Meta-data from clinical systems are absent
- Data can be easily extracted using softwar like WHONET
- Standardise surveillance using WHO guidelines
- Standardisation of testing protocols and quality control

Despite these methodological variations, ongoing comparative antimicrobial resistance data should soon be available within each system. However, one may wonder why their implementation has been delayed for so many years. It is clear that methodological and technological problems played a major role in this delay. Additionally, while public funding was crucially missing, renewed funding provided by the pharmaceutical industry for time-limited studies may have given the false impression that antimicrobial resistance surveillance systems were not needed.

- Methodological variations limits comparability
- Technological problems

Lack of standardization of the method used and the antimicrobial tested, as well as differences in quality of susceptibility testing results, are obstacles for reliable comparison of data originating from various microbiology laboratories [33]. The use of standardized methods, a quality assurance programme including internal quality control procedures and participation in an external quality control programme, are essential for obtaining good quality and comparable susceptibility data.

- Lack of standardisation
- Difference in quality and susceptibility results
- Standardised procedures are needed

Another problem is represented by possible differences in the frequency and the distribution of sampling among countries or regions due to physicians' habits and patient recruitment [39]. Similarily, the number of isolates from samples performed to screen patients for colonization by multi-resistant bacteria may vary greatly and these isolates should be excluded when comparing resistance levels. Because of these limitations, one should be extremely cautious when performing comparison of resistance levels from specimens other than the ones corresponding to clinical situations, where sample referral is the least likely to vary such as blood cultures or spinal fluid.

- Represented samples across countries is different
- Representativeness a limitation
- Performing comparisons is limited

Absence of consensus on a mininum set of data to be collected has also been an obstacle. Collection of national patient identification numbers is difficult or even illegal without special agreement in certain countries. There is also no agreement on whether hospital and unit codes, as well as type of unit categories, should be recorded. Some surveillance systems focus on isolates that are clearly associated with an infection such as blood or cerebrospinal fluid isolates. Although they provide a very specific estimate of the level of resistance in isolates of indisputable clinical relevance, these surveillance systems are probably not sensitive enough to detect emerging resistance mechanisms that are more likely to appear first in colonization and peripheral samples such as sputum or urine [40]. Consequently, surveillance of all body sites should be recommended for building an early warning system for antimicrobial resistance.

- Absence of data consensus
- No agreements between to collection identifiers
- Not sensitive enough to detect emerging resistance mechanisms

There seems to be an agreement on the necessity to collect quantitative susceptibility data, i.e. zone diameters or MICs instead of S:I:R interpretations. Quantitative data allow precise analysis of the whole distribution of isolates, early detection of emerging resistance, quality control of susceptibility testing and possible redefinition of breakpoints [41,42]. However, they may be difficult to obtain because zone diameters or MICs are generally not recorded in laboratory information systems.

- Agreement in susceptibility data to allow analysis

There are problems with the standardization of databases and the level of stratification of reports. In order to build surveillance systems, similar codes must be used when aggregating data from different microbiology laboratories. Transformation of data from various commercial susceptibility testing and laboratory information systems into standardized codes can easily be achieved automatically by using the BACLINK application of the WHONET software [5];

- Problems in standaridsation of databases
- WHONET and BACLINK are programmes to facilitate database standardisation

Surveillance systems should have clear objectives, i.e. to follow resistance trends, to evaluate the effect of interventions to control antimicrobial resistance and to provide alert for emerging resistance mechanisms. These systems should use existing electronic databases containing susceptibility patterns, which are available in most clinical microbiology laboratories, rather than requiring declaration of selected resistant microorganisms using a questionnaire. Recent studies comparing the sensitivity of these two methods found that the simpler and less expensive use of existing databases had a sensitivity equal or even greater than mandatory declaration of cases [55,56]. Again, considering additional workload and costs, epidemiologic typing and studies on the genetic determinants of resistance should not be implemented as ongoing surveillance but as time-limited projects within surveillance systems. Similarly, antimicrobial resistance surveillance systems should not primarily aim at identifying risk factors for acquisition, colonization or infection by antimicrobialresistant microorganisms but promote appropriate additional studies to study these risk factors when a problem has been identified.

- Should have clear objectives
- Use existing infrastructure

Establishment of an NRL.

The R. Lugar Centre for Public Health Research at the National Centre for Disease Control and Public Health was appointed as NRL, as a prerequisite for the PoP project implementation. During the project, laboratories sent bacteria isolated from blood cultures to the NRL for confirmation of identification and AST results, which strengthened the role of the Lugar Fig. 1. Essential elements for developing AMR surveillance, supporting the infectious diseases diagnostic cycle [4,5,13,15e19,22e27], NAP, national action plan; NRL, national reference laboratory; EQA, external quality assessment; IQA, internal quality assurance; SOP, standard operating procedure; LIMS, laboratory information management system. Centre as reference laboratory, and created ample opportunity to engage and communicate with clinical laboratories participating in the project. As a result, the Lugar Centre has maintained its status as NRL, and has been providing technical and human resource support across the country. In addition, the NRL processes blood cultures for clinics in the state programme that do not have the in-house laboratory capacity to perform such tests.

- National reference laboratory created a support network to communicate with other laboratories
- To create national reference laboratory EQA, interal assurance, LIMS are needed
- Support laboratory capacity for those who do not have it

Coordination at central level.

A national AMR expert committeewas established after the PoP project ended, which provides coordination and oversight at the central level, and evaluates the implementation of the NAP.

- Coordination at a central level with expert comittee

Legislation for surveillance.

Several orders and decrees have been adopted since 2015 to promote AMR surveillance through accountability, and to regulate, among other things, QMS in laboratories and monitoring of infections in hospitals.

- Several decrees have been adopted to promote AMR surveillance

Laboratory capacity and quality management

External and internal quality assurance, and accreditation. The NRL and the network laboratories have been enrolled in the CAESAR EQA programme since 2015. Furthermore, the NRL provides a quarterly national EQA programme for the country's laboratories, starting with 11 laboratories in 2016 and expanding to 25 laboratories in 2020. Results of this programme are used for feedback and education of the laboratories. In addition, the NRL currently mentors 17 laboratories to establish internal quality assurance programmes. Further quality control is ensured by confirmatory testing of exceptional phenotypes and unexpected results performed at the NRL, using phenotypic and genotypic methods. Since 2016 it is mandatory for laboratories to send to the NRL 'alert' organisms and organisms with unlikely AST results requiring confirmation. After the Lugar Centre initiated its function as NRL, it received ISO 15189 accreditation in 2017. In 2021e2022 the NRL will work to acquire accreditation as EQA provider (ISO17043).

- Laboratory capacity and quality management through EQA, IQA, and accreditation
- National reference laboratory provides EQA
- Feedback to help educate laboratories

Standardization and harmonization of guidelines and standard operating procedures (SOPs).

The NAP requires all laboratories to use the European Committee on Antimicrobial Susceptibility Testing (EUCAST) guidelines for AST. At the moment, approximately 60% of laboratories participating in the national network have adopted EUCAST guidelines, and the other 40% will switch in the near future. The PoP project's SOPs for blood sampling, sample processing and AST were shared within the national network and are now used by most of the hospitals and laboratories.

- NAP requires all laboratories to use EUCAST for AST.

Training and knowledge.

Training of clinicians and laboratory staff was an important aspect of the PoP project. Initially, training was given by WHO experts and consultants, but during the project training and support was taken over by the NLR. After the project, the NRL continued to train new members of the network and organizes annual microbiology network meetings, data collection workshops, symposia and lectures. As a member of the CAESAR network, Georgia has access to annual training and support from WHO/Europe and ESCMID.

- Training of clinicians is an important aspect
- NRL continued training of members of the network

Materials & supplies

Sustainable supply of good-quality materials. To ensure timely receipt of supplies, the NRL aids laboratories with tender procedures. The NRL performs quality verification testing and provides laboratories with a list of reliable manufacturers. This way, the quality of consumables procured by laboratories is ensured. In addition, the national laboratory network frequently communicates with manufacturers and providers within Georgia, to prevent and address supply-chain issues. In case of severe disruption of the supply chain, hospitals have emergency funds to procure materials following other procedures

- Materials need to be good quality
- NRL verified consumables
- Hospitals have emergency funds

Sample collection, data management and reporting

Access to laboratory information management systems. Enabling electronic data collection for laboratories using paperbased forms is one of the priorities for the national AMR committee. In 2019, a WHONET training was organized by the WHO for the national team and the network laboratories, but follow-up training is needed

- Electronic data collection using paperbase
- WHONET training implemented

Electronic data capturing and standardized and consistent data collection.

As a result of the PoP project, the same isolate record forms are used by all laboratories, or alternatively, AST results are entered in WHONET. The NRL developed a routine for standardized data collection from the network laboratories and enters the data into an electronic database to be exported to CAESAR. Postanalytical steps (e.g., data entry, feedback report) were standardized by the national AMR committee.

- AST results entered into WHONET
- NRL established a standardised data collection procedure

Malla et al. 2014

A total of 10 training workshops and five consensus meetings among participating laboratories were organised during 1999 to 2003, and from 2004 to 2012 annual refresher training was organised among participating laboratories. Training workshops mainly focused on improving the skill of local technicians, technology transfer and creating a pool of local [illegible]

- Training workshops helped train local technicians

Participating microbiology laboratories should use standardized methods, internal quality control procedures and participate in an external quality control programme. They should implement and adhere to a continuous quality improvement programme including the whole process from collection and analysis of specimens to the interpretation and validation of test results [39]. Although participation only of laboratories that satisfy external accreditation procedure has been advocated by some experts, it may not be feasible for the moment because of the small number of accredited laboratories.

- Laboratories should use standardised methods
- Adhere to quality assurance

Opintan et al. 2015

In the current laboratory-based surveillance of AMR, data sets were received and processed from .70% of the 24 laboratories that participated in the training workshop. However, six laboratories did not submit any data during the surveillance period. Our preliminary investigations revealed some lapses within these hospitals, including breakdown culture facilities, clinicians not making request for culture, and some internal managerial issues. More than two-thirds of the data analyzed in the current surveillance were from the southern sector, with less than one-tenth from the northern sector. In 2003, a similar nationwide surveillance of AMR also received a relatively small number of isolates from the northern parts of Ghana.19 Such disparities may introduce some biases while interpreting the results to direct antibiotic policy in Ghana. Considerations such as the general lack of access to health care facilities in remote and rural parts in the northern parts of Ghana, economic and social reasons, and patronage of traditional medications (herbs) compared to orthodox medicine have to be factored into interpretation of surveillance data.41 The Korle-Bu Teaching Hospital alone submitted .50% of the total data sets. Generally, academic tertiary referral laboratories are known to be over-represented in national and multicenter surveillance systems.42 This overrepresentation of data introduces some biases in the overall AMR surveillance results. The Komfo Anokye Teaching Hospital did not submit enough data in the current surveillance compared to their output in the previous study.19

- Investigations revealed lapses in hospital for microbiological services
- Internal management issues
- 2/3 of data from one locality
- One facility sent more than 50% of all samples
- Over-representativeness

Some proposals have suggested that AMR surveillance systems should be coordinated by PHRLs.43 In the case of Ghana, perhaps PHRLs are not yet ready to spearhead AMR surveillance activities. The mandate, direction, and functions of PHRLs in Ghana must be critically aligned to address the global public health threat of AMR. Grundmann et al suggested that global AMR surveillance systems must have separate functions including reference work, quality assessment, and the actual surveillance.43 In Ghana, the NPHRL may play a vital role in future laboratory-based surveillance, by participating in quality assessments. In the present study, faith-based and district hospitals also submitted data. Since these hospitals contribute greatly to the health needs of Ghana, they should be included in future national AMR surveillance programs. In general, the current study did not observe disparities in susceptibility results compared to the results of the previous study in Ghana.1

- Coordination of system through reference laboratory
- Laboratories are not ready to lead surveillance
- Mandates must be critically aligned
- Quality assessment by reference laboratories

Perovic and Schultsz 2016

AMR surveillance relies on diagnostic laboratories; in sub-Saharan Africa, the need for laboratory improvement is evident, with some countries in need of laboratory system built from the ground up. This prevalent lack of laboratory resources and the subsequent difficulty of obtaining accurate results on antimicrobial susceptibility testing is an important challenge to address when proposing a stepwise approach

- Surveillance requires diagnostic capacity of laboratories
- Lack of resources leading to inaccurate results

In Africa, the development and improvement of laboratory capacity, including standardised testing, external quality assessment programmes, procurement, and timely and cost effective reports, should eventually lead to the establishment of an integrated and coordinated surveillance system for AMR in the region, which will strengthen knowledge about AMR through surveillance and research. This surveillance should increase understanding of the implications of AMR and its epidemiology and allow for monitoring the effectiveness of guidelines and policy implementation. In addition, it would permit research and development of new diagnostics and novel technologies, optimisation of treatment, and other interventions as well as build human resource capacity as a secondary objective. Collaboration within a team of human and animal experts in AMR surveillance would allow for a One Health approach. This requires the establishment of coordinated surveillance in animals, the ability to control and regulate antibiotics use in animals, and the identification of alternative options for growth promoters in agriculture.

- Development and improvement of laboratory capacity needed
- External quality assessment
- Procurement
- Effective data reporting
- One Health approach

There are several approaches toward surveillance of AMR, including population-, sentinel- and laboratory-based surveillance. The general perception is that laboratorybased surveillance is currently the most efficient method of surveillance of AMR, which is what the WHO and GHSA advocate.1,4,5 However, laboratory-based surveillance is often biased, because of the potential barriers to and selection processes for submission of clinical specimens to laboratories for culture and susceptibility testing, particularly in resourceconstrained settings.2 This bias may result in laboratory based surveillance data being skewed toward a higher prevalence of AMR.

- Different approaches to surveillance
- Population, sentinel, and laboratory
- Laboratory based may be most efficient
- Laboratory surveillance may also be biased

For laboratory-based surveillance to yield comprehensive data, a functional infectious disease diagnostic cycle is required. This cycle includes clinicians submitting samples for culture and susceptibility testing, a bacteriology laboratory that can generate quality culture and susceptibility test results, and a reporting system that includes not only the clinician requesting the test, but also a laboratory information system (LIS) that can inform the surveillance programme, which may be steered by a central body (Figure 1). In addition, for laboratory surveillance programmes that are technically carried out by national public health laboratories, a crucial element of the cycle is the rapid and complete transfer of all required materials, isolates and/or data from peripheral laboratories to the national site, and back reporting of results to ensure continuous engagement of laboratories and clinicians.

- Comprenehsive data through clinicians submitting samples and quality microbiological testing
- Need for laboratory information system (LIS)
- Ensure continuous engagement of laboratories and clinicians

For a national laboratory-based surveillance programme to be successful, government commitment to support the surveillance programme for AMR at the country level is essential [illegible]

- Government commitment is crucial for AMR surveillance programmes

technicians, technology transfer and creating a pool of local technicians to compensate for transfer and retirements.

Consensus meetings were aimed to i) incorporate local innovative ideas generated by local experts (e.g. incorporating salmonella in the surveillance), and ii) encourage participating laboratory management in incorporating surveillance supplies in their respective laboratory procurement plan. Annual meetings were organised among key partners and stakeholders of the programme to i) communicate the progress and the key challenges ii) receive input from all key partners, iii) advocate for increasing the budget for the laboratories and iv) minimise the transfer of surveillance programme staff.

- Meetings engaged local experts
- Encouraged participation
- Inclusion of key stakeholders

The training programme and consensus meeting contributed significantly to national laboratory capacity building, standardisation and use of protocols for selected pathogens, and added new pathogens for surveillance as per local need. The programme also helped NPHL to build capacity for storing the isolates in Nepal and thereby create a sense of ownership of the programme (isolates and data generated through the programme). The programme created a pool of trained technicians who can compensate for transfer and separation of staff and contribute to expansion of programme staff. Stakeholders' meeting contributed in motivating the MOH, Nepal to increase the resources for laboratories, and incorporating the programme as a core activity of the NPHL and participating laboratories. The programme laid a solid platform for conducting laboratorybased surveillance in Nepal. Annual meetings and dissemination programme helped to establish a forum for discussion on surveillance of AMR in Nepal.

- Training programs helped to build laboratory capacity, standardisation
- New pool of technicians
- Stakeholder meeting contributed in motivating MoH

Electronic data capturing and standardized and consistent data collection.

As a result of the PoP project, the same isolate record forms are used by all laboratories, or alternatively, AST results are entered in WHONET. The NRL developed a routine for standardized data collection from the network laboratories and enters the data into an electronic database to be exported to CAESAR. Postanalytical steps (e.g., data entry, feedback report) were standardized by the national AMR committee.

Mugerwa et al. 2021

Governance structures are fundamental and pertinent for the pragmatic implementation of any strategy. Uganda's AMR surveillance system has been rooted and structured in a governance structure that embraces One Health. This structure comprises the One Health Platform, the Uganda National AMR Committee and the different AMR TechnicalWorking Groups defined by the National AMR Action Plan (Figure 1).

- Governance structure follows One Health approach

Uganda's AMR-NAP is a blueprint for the strategic plan on how the country will tackle AMR, and it recommends the development of critical documents to implement surveillance. The following documents were developed in the first year of the AMR-NAP; (a) Human Health AMR Surveil-lance Plan, (b) Human Health Antimicrobial Use and Consumption Surveillance Plan, (c) Diagnostic Stewardship Manual for AMR Site Surveillance and Clinical Protocols, (d) In-service Microbiology Training Curriculum, the Uganda National Policy for Bio-banking and Achieving Microbiology Isolates, and twelve sets of standard operating procedures (SOPs), which can be harmonized and adopted by all microbiology laboratories in the network with the inclusion of animal health, environment, and food and beverage laboratories. Taken together, these documents define the standard operating procedures for the delivery of the various components of the surveillance plans under the following key pillars.

- Documents produced for surveillance foundation
- Standard operating procedures for Bio-banking
- Inclusion of multiple sectors in approach

These share antimicrobial susceptibility data and isolates with the National Microbiology Reference Laboratory (NMRL). All of this surveillance and system design work is coordinated by the National AMR Coordination Centre, which is housed within the Ministry of Health Department of Laboratory (NHLDS).

- Coordination by AMR centre
- Sharing of data

It could also reflect inefficiencies in quality assurance in the diagnostic laboratories but highlights areas for monitoring and improvement in this surveillance framework.

- Inefficiencies in quality assurance

Uganda has leveraged existing Ministry of Health laboratory information platforms built on the health laboratory infrastructure to form a laboratory information system known as the African Laboratory Information System (ALIS). This information system has been developed as a generic tool for the country's laboratory system and programmed to integrate with other systems and tools like WHO-NET. The system takes all the critical variables on the patient laboratory request form (Table S2) to automatically analyse patientlevel data to generate epidemiological reports. The system in development and piloted at the National Reference Laboratory can interoperate with different micro-biology equipment platforms and technologies to share data in the form of analytical reports with the National AMR Coordination centre. We have piloted this electronic laboratory information system in at least four AMR Sentinel sites. These results are reported in this document and form the foundation for our AMR surveillance. Finally, our strategy is to develop an AMR real-time surveillance dashboard to inform local facility decision-making and resource allocation at the sub-regional and national levels. It is this information that would be shared with WHO via WHO-NET for the global AMR reports.

- Leveraged existing infrastructure to integrate other systems
- National reference laboratory can interoperate and share data
- Sharing information using WHO-NET

At the sub-national facility level, in the microbiology laboratory, request forms and the daily activity registers (Table S2) patient-level data is entered into WHONET. This software application analyses these data, and working with the facility implementing partner, data entry is validated at the facility level before being shared with the National Coordination Centre. Data validation at the national level is performed by the Technical Working Committee on surveillance that scrutinises the data before external uploading it onto the WHO-GLASS. SOPs and developed guidelines for data collection, sampling and reporting in surveillance sites are standardised: strengthen data management at AMR secretariat, NCC and sentinel sites by delivering IT infrastructure, paper-based forms and registers to sites, e.g., microbiology referral forms and registers; and train laboratorians to analyse microbiology lab AST data. Anonymised isolate level data are submitted to the AMR-NCC through an electronic system at the national level. This will eventually link with the national database (DISC) to evaluate the quality and report monthly AMR and microbiology data to DHIS2.

- Data entry through WHONET and analyse data
- National Coordination Centre does data validation
- SOP has developed guidelines to standardise data management

(Figure 2). Such commitment can best be demonstrated by the design of dedicated policies and plans for AMR control at government level, which include implementation plans and budgets. Governments are required to provide human and financial resources, which may come from a variety of sources. Governments of some countries may need to address specific deficiencies in supplies of water, electricity and infrastructure to allow laboratories to function appropriately. The national AMR surveillance implementation plan could be used as an advocacy tool for government funding to achieve this. Departments of Health and other governmental structures should work in conjunction with partners, such as the WHO, GHSA and the African Society for Laboratory Medicine (ASLM), on AMR programmes that require an inter-sectorial approach. The roles of WHO and ASLM should be in capacity building, financial support, promoting and communicating abroad on the AMR framework

- Design of dedicated policies
- Provide human and financial resources
- Physical deficiencies in water, electricity, and infrastructure
- Collaboration with other parties to support surveillance

Establishment of a national laboratory-based surveillance system at the country level, as established by each country's National Department or Ministry of Health, also includes the establishment of a coordinating centre with the responsibility to systematically collect, analyse and share data at the national and international level. Governments may decide to set up their coordinating centres differently, but all will need expertise in epidemiology, clinical microbiology, infectious diseases, veterinary medicine, data management and governance. Such expertise may be built and subsequently sustained through continuous education programmes that take place locally or within the region. For example, South Africa, Kenya, Ghana, Uganda, Nigeria and Burkino Faso all provide a Field Epidemiology Training Programme

- Establishment of coordinating centre for surveillance
- Reporting and sharing data
- Continous education needed
- Expertise needed for surveillance

Establishment of at least one reference laboratory that can undertake infectious disease diagnostics and antimicrobial susceptibility testing, and that has competence in phenotypic and genotypic confirmation of the presence of resistance genes, is required. Initially, in the absence of capacity to perform advanced phenotypical and molecular testing in the national reference laboratory, a clinical hospital bacteriology laboratory with demonstrated capacity to perform these functions, could be selected as a reference laboratory. In addition, instead of performing genotyping at the national level, molecular typing and gene characterisation could take place in another reference laboratory in the African region. National reference laboratories perform their role in close collaboration with provincial and district clinical laboratories, which generally receive samples directly from patients. Together, these laboratories form the national surveillance network. Countries should also consider including private laboratories in their network. Although specifics vary by country, such laboratories may receive large numbers of samples from a substantial proportion of the population. Strategies on how to involve and include private laboratories in national AMR surveillance networks must be developed to achieve this inclusion.

- One reference laboratory with competence is needed
- Molecular typing and other procedures carried out at another reference laboratory with competency
- Together these laboratories form a network
- Inclusion of private laboratories in networks

The organisation of regional AMR surveillance programmes creates opportunities for concentrating specific expertise in dedicated national laboratories, thus potentially reducing costs and increasing efficiency. In addition, such regional programmes may provide support for solving logistical issues, such as those described below.

- Organisation of regional programmes create opportunities to concentrate expertise to reduce cost and increase efficiency

The quality of laboratory-based AMR surveillance data depends to a large extent on the microbiology laboratory. Firstly, the quality of a laboratory system should be assessed in order to identify gaps and areas for improvements. In order for participating laboratories to meet minimal quality standards requirement as defined by the network (e.g., accreditation, external quality assessment programme, and other standards), establishment of a laboratory quality system is essential. However, the logistical difficulties of joining external quality assessment programmes and receiving quality control materials present significant challenges to countries in sub-Saharan Africa, because of customs and other policies for shipment of samples and microorganisms between different countries.

- System should be assessed to identify gaps
- Laboratories meet minimal quality standards required
- Establishment of quality assessment is necessary

Laboratory assessments should include pre-analytical, analytical and post analytical steps for reporting of antimicrobial susceptibility testing (AST) and its limitations. For the pre-analytical phase, diagnostic stewardship is critically important. Diagnostic stewardship is the process that guides and improves the use of microbiological tests to ensure they are timely, appropriate and accurate. Reports sent to clinicians should include a feedback form to help on-going improvements. To apply required standards for AST, laboratories must have the required infrastructure, equipment, supplies and staffing in place. Laboratories should be able to identify gaps and recommend improvements. For example, some laboratories may have all the equipment needed but no supply of reagents or other consumables needed for basic microscopy, culture and AST implementation. This, in turn, affects diagnostic stewardship as clinicians may lose confidence in laboratory reporting when timely, appropriate and accurate results cannot be achieved. A country's national AMR surveillance plan and the regional AMR surveillance network may provide for policies and procedures that make timely ordering and receipt of consumables and other supplies feasible and affordable. ASLM could play an active role in such advocacy activities. Laboratory experts should be involved in ordering supplies to avoid receiving supplies that are irrelevant, not useful or of poor quality.

- Laboratory assessment should look at AST for limitations
- Feedback to clincians post assessment
- Required physical infrastructure needed for assesment
- Staffing must be sufficient
- Laboratory experts should be involved in ordering supples

The use of a LIS is limited in most LMICs, which is unfortunate, as it improves AMR surveillance by reducing workload and errors, thus improving the overall quality of microbiology laboratories. Laboratories should use LIS to retrieve and share AST data with national and regional bodies (Figure 1). Whilst databases preferably allow sharing of data, confidentiality must also be guaranteed. The WHONET data management software provides these features and is freely available.12 Other initiatives such as those from the RESAOLAB network13 have created their own open source LIS that includes an option to store and report AMR data.

- Laboratory information systems are limited
- Improves surveillance through decrease of workload and increase laboratory capacity
- WHONET is an example software to facilitate sharing of AST data

Human resource capacity may be the most challenging issue, as shortages of qualified and trained professionals can have a serious negative impact on the ability of laboratories to perform testing. Human resources are a weak spot in microbiology and have been ignored. Quality microbiology requires sufficient number of qualified technicians, as well as supervision by MD or MSc level clinical microbiologists who are capable of reporting results to clinicians. Finally, veterinarians should be involved in the process of developing surveillance systems to strengthen diagnostic stewardship in animal health, provide assistance about status of AMR in animal health, and implement preventative measures in animal health, if needed at a later stage. For capacity building that includes education, training and refresher courses and other forms of networking, the WHO and ASLM should be excellent sources of support.

- Human resource capacity is most challenging
- Needed for trained professionals
- Vetenarians should be involved in the process to improve AMR stewardship

Considering the growing impact of AMR worldwide and the lack of quality surveillance systems in the African region, an enormous effort must be undertaken through international and multi-

- Lack of trust, demand and capacity

To be most effective, antimicrobial surveillance systems should be coordinated and complementary to each other in respect of the National Action Plan. A cohesive surveillance system looks to the One Health approach to provide a more complete picture of AMR and AMU and facilitate analyses of trends over time and space and of relations among sectors

- Effective surveillance should be coordinated
- Provides more complete picture of AMR

After primary microbiology cultures and sensitivity testing, isolates are sent to the National Microbiology Reference Laboratory (NMRL) for validation and archiving. At the end of every quarter of a year, the NMRL must conduct site visits for retrospective on-site data cleaning and support supervision for improved microbiology culture and sensitivity laboratory procedures. It is noteworthy that for some sites, data cleaning and entry are performed in real-time using WHONET software

- Provides more complete picture of AMR

Sentinel sites are connected to a national microbiology network that includes the National Microbiology Reference Laboratory, academia, private sector and other research laboratories performing microbiology culture and sensitivity testing. All laboratories in the network are required to refer isolates to the National Microbiology Reference Laboratory for validation and archival in the biorepository. In addition, all regional reference laboratories participate in the Laboratory Quality Management Systems for internal quality control. A new scheme for microbiology in-country EQA was recently rolled out by the National Microbiology Reference Laboratory, beginning with Gram's reaction and panel test to 16 laboratories in the network. Antimicrobial susceptibility testing is performed following the CLSI guidelines.

- National microbiology network with reference laboratory incorporates all sectors
- Laboratory quality management systems needed for quality control

The WHONET is integrated with the existing laboratory information systems as customised by the AMR surveillance in Uganda; these include the African Laboratory Information System (ALIS). Figure 6A–C displays the functioning units of the AMR surveillance system and how the data generated displays the WHO-GLASS priority pathogens and specimens. This is ideal for any AMR surveillance system that focuses on AMR priority pathogens and specimens. When integrated, these two software platforms will provide the basic digital infrastructure for local surveillance and reporting to WHO-GLASS and the AMR- National Coordination Centre of the Ministry of Health. The exported data sets were preliminary analysed and generated preliminary reports as presented in the results section below.

- WHONET integrated with existing laboratory information systems
- Software integration will provides local surveillance infrastructure

The fundamental limitations of the system are as follows:
Non-uniformity of testing isolates against different priority organisms, thus a need to adopt the standardized national microbiology protocols.
Lack of awareness leading to less utilization of the microbiology laboratories, hence less demand.
Inadequate microbiology supplies and logistics. This has failed to provide results consistent with culture and sensitivity test requirements. A large share of these supplies is donor-funded, thus raising questions for the program's sustainability at the national level.
Lack of human resource capacity for microbiology susceptibility testing.
The small number of sentinel sites submitting data limits the magnitude of the data. Significant efforts are needed to scale up to other sites in the shortest time possible

- Non-uniformity of testing isolates
- Further need for standardised microbiological protocols
- Lack of awareness and utilisation of laboratories
- Lack of human resource capacity for microbiological susceptibility

Nabadda et al. 2021

In 2015, Uganda responded to the WHO call for countries to enroll in the GLASS program. By enrolling in the GLASS program, Uganda committed to collecting and sharing national AMR surveillance data. As part of this process, the country also acquired the WHONET software [18] used to report AMR surveillance data. A WHO GLASS focal person was designated by the MoH to support the coordination of AMR data validation, quality assurance, and the reporting process. Uganda now participates in the annual AMR data submissions to GLASS, and the country AMR data are part of the WHO global AMR surveillance reports [19].

- Uganda enrolled into GLASS
- WHONet software used with MoH coordinating AMR data validation

The National Coordinating Center (NCC) at the MoH has been set up to oversee the national AMR surveillance program in human health, including the collection and aggregation of data from surveillance sites. The NCC works in collaboration with the Uganda National AMR Sub-Committee (UNAMRsC) of the One Health approach to provide strategic oversights of the national AMR program. The UNAMRsC has also been established as part of the governance structure for AMR in the country. The membership of the UNAMRsC also includes representation from other relevant line ministries, such as animal health, wildlife, and the environment. The mandate of the UNAMRsC includes defining the national AMR surveillance objectives; developing and disseminating protocols; coordinating data collection, analysis, and reporting; and reviewing data before reporting to GLASS. To date, the NCC has supported the development of key AMR surveillance documents, including national AMR surveillance plans, protocols, guidelines, curricula, and microbiology standard operating procedures

- NCC oversees surveillance program
- Works in collaboration with sub-committees
- Representation from other committees
- NCC supports policy, plans, and operating procedures

However, the capacity of the Central Public Health Laboratories has been built gradually, and it is now the designated national microbiology reference laboratory for AMR surveillance. The capacity built at Central Public Health Laboratories includes human resource development, quality management systems toward accreditation, isolate transportation, enhanced biorepository, and enrollment of laboratories in an External Quality Assurance scheme. In addition, the state-of-the-art Becton and Dickson–manufactured equipment, including matrix assisted laser desorption/ionization time of flight [20] and Phoenix M50 [21], has been installed at the reference laboratories and BACTEC blood culture systems, including FX 200 and FX40 [22], at selected RRH laboratories. These results support bacterial ID and AST.

- Laboratory capacity built over time
- Enrolment into EQA
- Laboratory equipment installed

However, the capacity of health facilities to conduct AMR surveillance varied between the different health facilities. As a result, selected sites have reported AMR surveillance data to the WHO and the capacity of the surveillance sites has been gradually developed. The Medicines and Therapeutics Committees oversee the implementation of the AMR surveillance program at the surveillance sites, which have been trained in collecting, analyzing, and reporting epidemiological, clinical, and laboratory data. The Medicines and Therapeutics Committee is usually headed by a senior consultant (Internal medicine, Gynecology, Surgery, and Pediatrics) who leads the stewardship of the AMR program at the site.

- Varying capacities across health facilities
- Committes have overseen the implementation of AMR surveillance
- Headed by senior consultant

The AMR surveillance subcommittee technical working committee representatives conducted [illegible]

- Technical committee conducts quality assessments

sectorial collaboration. A recent report analysing the AMR surveillance capacity of the East Africa Public Health Laboratory Network by the Center for Disease Dynamics, Economics & Policy, commissioned by the World Bank, summarised the following key issues for laboratories to carry out AMR surveillance:
• Lack of demand of bacteriology diagnostics from clinicians, related to length of time to get results (at least two days); lack of trust in results; lack of laboratory capacity for blood cultures, which are needed for many of the most serious, life-threatening infections.
• Low priority given to bacteriology diagnostic supplies by hospitals and other decision makers who control purchasing.
• The multi-component nature of bacterial culture and antimicrobial susceptibility testing, which makes it especially vulnerable to weak supply chains and frequent stock outs; results cannot be obtained if essential components are unavailable when testing is needed.
• Lack of recognition that microbiology requires dedicated, trained personnel, leading some facilities and/or ministries of health to rotate staff in and out of microbiology.
• Few options for automated testing relative to haematology, chemical pathology or HIV testing, which may be more satisfying to staff, leading to low morale.
• Requirement for patients to pay out of pocket for diagnostic tests in many countries.

- Lack of trust, demand and capacity for microbiological services
- Low diagnostic supplies
- Weak supply chains
- Need for trained personnel
- Need for personnel engagement
- Financial costs

Schrijver et al. 2017

At the national level, there is no uniform European system of monitoring and reporting of AMR in zoonotic agents and veterinary pathogens in livestock (Table 2). In several countries, national AMR reports are lacking, or information is only available in unpublished databases in the local native language. In many available reports, details are lacking about sampling methods, antimicrobial methods and cutoff values used (Tables 2 and 3). Reports also greatly differ in content and layout, and materials and methods of reports are not homogenously presented. As a result of these shortcomings, a critical appraisal of the reports to determine the quality of the surveillance programmes cannot be performed.

- No uniform reporting of AMR
- National AMR reports lacking or not available
- No details in sampling
- No homogenity in reporting of content

Only four of 31 countries (DE, DK, SE and UK) published yearly reports with results of mandatory EU reporting and monitoring of veterinary pathogens in English. Further, only 11 of the 31 countries published annual national AMR reports more than 2 years in a row in English or a local native language. Three countries do publish on a regular basis, but reports are based on 2-year periods (BE (Federal Agency for Safety of Food Chain, FAVV), FI, FR). Fourteen of 31 of the countries published national reports fully in English. Five countries published national reports written in a local native language; three of these were accompanied by an English-language written abstract. Two countries only published national reports once: Spain (Veterinary Antimicrobial Resistance Surveillance Network (VAV), 2005) and Italy (Italian Veterinary Antimicrobial Resistance Monitoring (ITAVARM), 2003).

- Hetereogeneous timelines for reporting
- Languages of reporting

The mandatory EU antibiotic resistance programmes in foodproducing animals currently implemented in EU countries provides only limited information. Many EU countries supplement this lack of data with national surveillance programmes for antibiotic resistance of veterinary pathogens, which however are not linked with the mandatory reporting systems. The industry-funded programmes are executed independent from the EU and national programmes. Although VetPath does not provide direct evidence for susceptibility of human pathogens for antimicrobials used in human medicine, many of the antibiotics evaluated are also used in human medicine, and several of the pathogens tested, notably S. aureus, E. coli and Streptococcus suis, also bear relevance as potentially human pathogens.

- Limited information on food producing animals
- Lack of data supplemented by national surveillance

At the national level, AMR data contain a great variety in sample origin (diseased or healthy animals), sampling numbers, test methods (dilution or diffusion tests, genetic tests), standardization (EUCAST or CLSI guidelines) and cutoff levels (clinical or epidemiologic cutoffs), which as a result does not allow comparison of resistance data among countries [13e15]. Better harmonization of sampling methods exists for monitoring AMR in E. coli, Enterococcus spp. and Campylobacter spp

- AMR data is heterogenous
- Limits comparability
- Better harmonization of sampling exists for specific species

Although the EU/EFSA/ECDC programme increases harmonization of AMR monitoring, results are published with a 2-year delay. The programme provides relevant information on AMR patterns on ESBL-, AmpC- and carbapenemase-producing bacteria with zoonotic potential from cattle, pigs and poultry and meat at retail. The CEESA programme applies a more uniform methodology than the EU/EFSA/ECDC programme. The availability of a laboratory data network that is able provide online data on AMR would allow the use of more recent data and would result in a shorter delay in taking action

- EU/EFSA/ECDC programme helps harmonization of results
- CEESA applies mode uniform methodology
- Online network would provide more recent data

At the national level, AMR data contain a great variety in sample origin (diseased or healthy animals), sampling numbers, test methods (dilution or diffusion tests, genetic tests), standardization (EUCAST or CLSI guidelines) and cutoff levels (clinical or epidemiologic cutoffs), which as a result does not allow comparison of resistance data among countries [13e15]. Better harmonization A mandatory network needs to be set up, with clear guidelines on how to report on samples, sampling methods, susceptibility tests, which cutoff values have to be used and results, but also availability of the reports in English would be preferable, as well as a much shorter delay between sampling and publication so that we can more quickly identify changes in AMR in zoonotic bacteria and veterinary pathogens.sampling methods exists for monitoring AMR in E. coli, Enterococcus spp. and Campylobacter spp

- Network with clear guidelines on reporting sampling and testing needed
- Single language use for comparability

Seale et al. 2017a

We recommend a sentinel surveillance system8, with step-wise expansion to increase the numbers of participating sites and their scope. In the first instance, we propose that countries should identify or develop capacity in a single site that can undertake surveillance to an acceptable core standard. Having achieved that standard, the primary site should support the development of good practice in secondary sites, with the long-term aim of building a comprehensive network of sentinel sites which can provide highquality representative AMR data. Sentinel sites that have achieved core capacity may aspire to higher standards (extended and advanced, Supplementary File 1: Appendix D) by developing and extending their capabilities.

- Sentinel surveillance system with stepwise expansion recommended
- Single site development with future expansion
- Approach provides high quality representative data

Public health surveillance is usually legally mandated by the national government. For public health surveillance programmes, the probability and the magnitude of harm to the population arising from not reporting surveillance data must be moderate to major to justify the use of [illegible]

- Mandates by national government

The AMR surveillance subcommittee technical working committee representatives conducted data quality assessments on a quarterly basis to inform key performance indicators and reports submitted to NCCs. Uganda has been consistently submitting data to GLASS reporting since its enrollment in 2016

- quality assessments

The findings of our surveillance program show the feasibility of setting up a national AMR surveillance program based on the WHO GLASS manual recommendation while building systems for quality assurance, data sharing, linking results to patient care, and building partnerships. AMR is a global health threat, and the establishment of national surveillance systems is necessary to identify the emerging drug-resistant infections [2]. The African region still has suboptimal microbiology laboratory capacity and surveillance systems for AMR [3].

- Success in building surveillance system
- Based on GLASS manual recommendations
- Suboptimal microbiological laboratory capacity

The success of establishing the national AMR surveillance program in Uganda highlights the feasibility of implementing the WHO GLASS program in LMICs. AMR surveillance programs are fundamental in Sub-Saharan African countries such as Uganda for generating antibiograms that can inform the development of treatment guidelines and antibiotic procurement plans and contribute toward standardized reporting [25,26]. Clinicians at surveillance sites can also access bacterial ID and AST results to inform patient care because of the availability of strengthened quality microbiology services.

- Success in Uganda highlights feasibility of implementing AMR system
- Programs are fundamental to standardisation of reporting

In Uganda, the AMR surveillance system has been established using a systematic capacity-building pyramid model [17] and in alignment with the London School of Hygiene and Tropical Medicine stepwise road map for participating in GLASS [16]. The rolling out of the NAP for AMR [13], AMR national and subnational structures with Terms of Reference, and supporting surveillance plans and protocols has strengthened antimicrobial stewardship. In addition, the establishment of data-sharing platforms, including software programs such as WHONET [18], has supported data collation, analysis, reporting, and electronic archival, supplementing the existing paper-based methods.

- Designed with pyramid model with London School of Hyigene road map
- Data sharing platforms help support data collation, analysis and reporting

The main limitations included suboptimal recovery of the AMR GLASS priority pathogens and inconsistent setting of the recommended antibiotics against the pathogen in the laboratory. This was attributed to the fact that staff members were still undergoing comprehensive training on microbiology skills, stock-outs, and acquiring extensive knowledge on AMR surveillance [32]. The number of samples sent to the microbiology laboratory was relatively low, coupled with low rates of completion of the microbiology laboratory request forms. This was partially attributed to the lack of a laboratory-clinician interface to bridge these anomalies.

- Limitations in recovery of pathogens
- Staff still undergoing training on microbiology skills
- Samples sent are relatively low with low completing rates
- No interface between clinician and laboratory

In Uganda, the AMR surveillance system has been established using a systematic capacity-building pyramid model [17] and in alignment with the London School of Hygiene and Tropical Medicine stepwise road map for participating in GLASS [16]. The rolling out of the NAP for AMR [13], AMR national and subnational structures with Terms of Reference, and supporting surveillance plans and protocols has strengthened antimicrobial stewardship. In addition, the establishment of data-sharing platforms, including software programs such as WHONET [18], has supported data collation, analysis, reporting, and electronic archival, supplementing the existing paper-based methods.

Queenan, Hasler and Rushton 2016

A centralised programme is required to set standards for data collection, which is critical at all levels for ease of analysis and interpretation. This will also improve communication and networking between disciplines and sectors through shared meetings, discussions and report editing.

- Standardisation of data collection is needed
- Improve networking between disciplines

Centralised leadership and budgeting will be responsible for supporting and enabling peripheral capacity and compliance. However, this ideal, multidimensional, integrated surveillance may be difficult to achieve across all categories given the high level of capacity and logistics required and the commonly found barriers between siloed institutions. A tiered approach to integration may assist in building capacity. Within each tier, globally recognised techniques and standards must be adopted and adhered to. The initial tier would involve relatively standard collection, sharing and analysis of data and therefore involve less integrated interpretation. The progression into the higher tiers would involve broadening data collection sources (from Fig. 1). Thiswould require a shift in institutional mindsets to support the increasing degrees of co-operation and integration required to facilitate the analysis and interpretation of results and evolution of joint recommendations

- Central leadership and budgeting is needed to enable capacity
- May be difficult due to the high capacity
- Globally recognised techniques and standards must be adhered
- Data workflow standardisation with interpretation

A lack of data quantity and quality persists, the latter affected by a lack of standardisation [30]. There is still insufficient comparable data of AMR in humans and livestock to develop global mapping of resistance and to allowaccurate comparisons between humans, various livestock species, industries, countries and regions [31].

- Lack of data and standardisation
- Insufficient comparable data to develop global mapping

In 2012, the WHO published results of a survey of existing surveillance networks [32]; 5 international and 22 national networks responded. Only 30% had a nationally co-ordinated body, whilst <40% had formal quality assurance requirements and antimicrobial usage data were generally absent. In the WHO's global report on surveillance of AMR [10], there was no formal consensus on methodology and data collection amongst the 129 contributing member states. At the European level, although EARS-Net advises and encourages standardisation using EUCAST guidelines, only 64% of the participating laboratories do so [23]. Global systems are reliant on data collected nationally. Therefore, standards within data collection need to be applied at source to allow meaningful interpretation at all levels, which will require significant investment in developing capacity. Therefore, in the next section we discuss a business case for a One Health approach to AMR surveillance.

- Only 30% have national coordinating body
- Low formal quality assurance
- No formal consensus on methodology
- Global systems reliant on national data

Although costs are incurred in the setup of or conversion into a One Health system, savings are expected from the effects of shared and more efficient use of resources, shared running costs and improved outcomes. Using the H5N1 (highly pathogenic avian influenza) campaign as an example, theWorld Bank estimated cost savings of 10–30% from joint investment and 20–40% in costs of ongoing surveillance through shared staff and facilities. Further savings in training and research are estimated at 5–10% [34].

- Initial costs associated
- However, cost benefits for one health implementation

individual patient data without individual patient consent9. In this context, the WHO has recently recognised AMR as a significant potential global health threat10. Reporting the characteristics of resistant pathogens rarely represents a threat to patient confidentiality, but the inclusion of simple clinical data such as age, sex, collection date, specimen type and syndromic diagnosis adds considerable value to the information obtained from the laboratory, and there are clear benefits from AMR surveillance at patient, pathogen and population levels6. Examples of the application of AMR data include timely feedback to clinicians to support patient care and enable rationalisation of antibiotic treatment; use of data to inform local antimicrobial prescribing guidelines and infection control policies; analysis of clinical surveillance data (at international, national and / or local level) to enable public health interventions; cross-policy collaboration and support for research institutions to analyse clinical surveillance data, adopting a One Health to understand the emergence, transmission and dissemination of pathogens at the human-animal interface.

- Inclusion of meta data adds considerable value to laboratory information
- Cross-policy collaboration and support for research institutions is needed

Given the need to integrate data from different sources, including individual patient data, it is essential that there are data governance agreements and procedures in place. These should protect the confidentiality of individual patients, but also facilitate the sharing of AMR surveillance data to inform policy locally, nationally and internationally. To meet ethical obligations, technical, legal and/or political barriers to data sharing11 must be overcome, and best practice for data collection ensured. For these reasons, a successful AMR surveillance programme requires clear political support, and should engage accordingly with the relevant government bodies11.

- Essential for data governance agreements needed
- Protect confidentiality but facilitate data sharing
- Successful AMR programme requires political support

Each country should develop its own organisational structures (Figure 1), and define terms of reference. While the governance structure may vary, important factors include identification of a National Coordinating Centre (NCC), convening a technical team, and strong engagement with the Ministry of Health, reflecting the national importance of AMR surveillance in health systems.

- Develoment of government structures
- NCC convening a techanical team with strong government engagement

The NCC should include a committee of multi-sectoral stakeholders to support a One Health approach at national and international levels. This committee could be, or could develop from, the national working group on AMR as established by GARP, or the committee responsible for the NAP. The committee should report to the appropriate national body, for example, the Ministry of Health, and, where appropriate, collaborate with a relevant external organisation/funder.

- NCC should engage multi-sectoral stakeholders
- Appropriate reporting to national body

The roles and responsibilities of the committee should be set out with formal terms of reference. Membership should include relevant technical experts and stakeholders, although individuals may fulfil the remit of more than one technical brief. A typical committee may include the following representatives: technical team leader, Ministry of Health, Ministry of Agriculture, national public health institute, coordinating AMR laboratory, international stakeholders, clinical microbiologist, data manager, public health analyst, laboratory manager, hospital manager, adult physician, paediatrician, pharmacist, veterinarian, infection control manager.

- Roles for stakeholders need to be specific
- Represent multi-sectors

The functions of the NCC include:
1. commissioning a situational analysis of current capacity and sustainability for AMR surveillance
2. national strategic planning for AMR surveillance
3. oversight of AMR surveillance implementation at a national level against key performance indicators

- NCC looks at sustainability
- Engages in planning and development of performance indicators

The NCC should have oversight of the technical team to: monitor quality assurance, support capacity building through training of national and site level participants, determine national priorities for pathogens in AMR surveillance in addition to those identified as priority pathogens by the WHO, review the scope of AMR surveillance as capacity develops, integrate a One Health approach, review the introduction of new technologies, support research programmes to use AMR surveillance platforms, collaborate with neighbouring countries and across international regions, and develop and expand regional networks.

- NCC monitor quality assurance and training
- One Health approach
- Support research and innovation
- Expansion of networks

The NCC should be headed by a named National Coordinator for AMR surveillance from a key stakeholder institution, such as the Ministry of Health, Institute of Public Health, or similar organisation. The National Coordinator should be supported by a technical team responsible for training, standardisation and quality assurance. Where appropriate, the technical team may be led by the National Coordinator.

- Headed by National Coordinator
- Support research and innovation

The NCC should collaborate with international stakeholders and funding bodies, such as the Fleming Fund, the US Centers for Disease Control and Prevention, the Institut Pasteur, the European Centre for Disease Prevention and Control, the Bill & Melinda Gates Foundation, and major non-governmental organizations including Médecins sans Frontières, the Global Health Security Agenda and the WHO.

- Collaboration with stakeholders and external funding bodies

Sentinel sites should determine and define their own organizational structures, and how this fits into existing hospital and laboratory administration systems. There should, however, be a Site Coordinating Committee (SCC), with defined terms of reference, and which includes relevant representatives, for example site leader, hospital administrator, data manager, laboratory manager, clinical microbiologist, adult physician, paediatrician, infection control manager, pharmacist, veterinary practitioner, public health specialist.

- Determine their own organizational structures
- Site coordinating commitee

Rattanaumpawan et al. 2018

Data collection by means of an electronic database andWeb- or Internet-based methods was commonly used worldwide. However, using an electronic database requires high investment in hardware (eg, computers, mobile phones, other electronic devices), sophisticated computer programs and software, and well-trained personnel. Therefore, open-access, user-friendly information technologies for electronic surveillance and real-time warning would be beneficial in health care facilities for the early detection of emerging infectious diseases, including AMR infections; they would lead to appropriate management of affected patients and timely, effective prevention and control of infections. Alternatively, Web- or e-mail–based data collection would be more effective in resourcelimited settings

- Electronic data collection used
- Requires high investment in hardware
- Investment in training of personnel

WHONET is an open-access software (available from the WHO Collaborating Centre for Surveillance of Antimicrobial Resistance) for the management and descriptive analysis of microbiologic data; it is an information technology forAMRsurveillance that has been used in >3,000 laboratories in >120 countries.

- WHONET used to capture microbiological data
- Is commonly used globally

Despite the higher cost of electronic surveillance systems, many studies revealed that such systems could reduce the time to detect targeted infectious diseases.10,14,16,19,56,62,64,98 Furthermore, an electronic surveillance system could provide better data quality, including data accuracy (sensitivity and specificity in capturing cases with infectious diseases),9,64,68,71,99 and better data completeness.

- Electronic surveillance systems increase timeliness
- Electronic systems give better data quality

Rempel Pitout and Laupland 2011

There were issues with case ascertainment for four of the studies examined. Three had concerns with case ascertainment because it was possible that not all isolates were tested for resistance and were, therefore, given a score of '1' because some of the isolates not tested may have met the case definition of resistance (20,26,34). One study had substantial issues with case ascertainment and was deemed poorly protected from bias due to the fact that the study was describing antimicrobial resistance rates, but excluded isolates based on intrinsic resistance and was given a score of '0' (22).

- Not all isolates tested for resistance
- Poorly protected from bias

sampling bias was precluded because sampling was not performed and, therefore, were scored '2' for sampling bias (31-34). Seventeen studies scored '1' (13-30). Of these, seven had concerns with sampling because they asked for a certain number of consecutive isolates (13-15,17,19,23,24). Another five studies had sampling issues because they asked for a certain number of isolates with no explicit statement as to the method with which these isolates were to be sampled (18,20-22,28). In addition, five studies scored '1' solely on the basis that the sampling of geographical centres was not reportedly based on true random sampling (16,26,27,29,30). The study scoring '0' did so because it had no systematic means of selecting participating centres based on the area it sought to measure and samples were obtained from only two laboratories (25)

- Concerns for sampling
- No explicit statement on how samples were selected
- Geographical centres not reported

Laboratory practices and procedures was not a substantial area of concern for bias for most of the studies. Eighteen of the studies had reported thorough protocols, quality-assurance programs, standardized testing and/or centralized testing (13,14,16-24,27-32,34,35). Three of the studies had some concerns with laboratory practices and procedures because documentation was lacking regarding standardized testing among all laboratories or they did not report on any qualityassurance/quality-control programs in place at the laboratories (15,25,33). One study had substantial concerns with a risk for bias because its laboratory methodology was poorly reported (26).

- Standardised protocols in place
- Concerns with standardised testing
- Poor reporting of methodology

Multiple counting is a significant potential issue and arises when a case is counted more than once for the same episode of disease (3). While no universal 'gold standard' definition exists, it is generally accepted that only the first isolate per patient per episode of disease should be counted (36). Several studies have found that failure to remove duplicates or multiple counting of the same isolates results in an overestimate of both occurrence and rates of resistance (3,37,38). .

- Multiple counting is a significant issue
- No standard definition to remove multiple counting
- No standard definition to remove multiple counting

Sampling bias occurs when the sample under study differs in some systematic way from the larger population of interest (3). One way to minimize or avoid this bias is to include all of the population of interest. However, such population-based studies are often practically difficult to conduct and, in most cases, sampling must be performed (31-34). To be unbiased, a sample should be randomly selected from the overall population of interest. This, however, does not appear to be a common practice in surveillance studies, and convenience sampling from selected laboratories is the usual and potentially highly biased practice. In multicentred studies, hospital-based laboratories – particularly academic tertiary care referral centres – are frequently over represented and, as a result, resistance rates are typically higher than in the population at-large. In addition, the time of day, day of the week, and season of the year may have a significant influence on rates of disease and antimicrobial resistance (38-40). The practice of collecting consecutive samples over a defined period may then be highly influenced by when and where these are obtained.

- Sampling bias present in reporting
- Random sampling should be used but not standard practice
- Convenience sampling used
- Limitations to convenience sampling

The site leader would be expected to have project management and programme implementation skills, and should report to the NCC. The role of the SCC, led by the site coordinator, includes: 1. working with the national technical team to facilitate a situational analysis of current capacity and sustainability at the site 2. planning strategic priorities at the site 3. oversight of AMR surveillance implementation at the site and reporting against key indicators

- Coordinator collaboration with stakeholders
- Monitor surveillance capacity and performance

A coordinating AMR laboratory should be identified / established for AMR surveillance. This may already be in place, or may be developed as part of the capacity-building process. Where there is no capacity for a coordinating AMR laboratory, countries should collaborate with neighbouring countries, which may be able to provide this service.

- Limited laboratory capacity assisted through working with neighbouring country

Coordinating AMR laboratories should be accredited, or be working towards laboratory accreditation12. Their staff should be trained by the technical team and / or external partners to provide training for sentinel site laboratory staff, using a "Train the Trainers" approach (Supplementary File 1: Appendix A). The functions of the coordinating AMR laboratory are: 1) core laboratory processes (Supplementary File 1: Appendix D) 2) participation in internal quality assurance 3) participation in external quality assurance through appropriate international schemes 4) provision of a reference service for core organism / antimicrobial combinations as a minimum, for borderline isolates or isolates with unexpected or unusual resistance profiles, and collaboration with international centres to monitor emerging resistance patterns 5) assisting sentinel site laboratories to procure equipment and reagents, in collaboration with the NCC 6) maintaining a biorepository for bacterial isolates 7) promotion of good practice (including development of national SOPs) to ensure standardisation and quality control 8) training staff at sentinel site laboratories 9) facilitating the development of internal quality assurance at sentinel site laboratories 10) provision of external quality assurance across sentinel site laboratories if they do not already participate in external quality assurance (EQA) (for example, by testing a subset of isolates from the sentinel site laboratories and providing feedback)

- Coordinating AMR laboratory
- Staff training using Train the trainers approach
- Participation in quality assurance
- Provision of testing standards
- Collaboration with international centres
- Providing feedback mechanism

To promote awareness of AMR surveillance, education and training should be integrated into local and national education programmes, across all the disciplines required for AMR surveillance. These include clinical, laboratory, information technology and public health training (Supplementary File 1: Figure 3). Teaching on AMR should be introduced into formal training pathways, including undergraduate and postgraduate curricula across these disciplines. AMR awareness should also be developed through continuing professional development (training days, workshops) at site, regional, national, and international levels. Such training should incorporate e-learning options and specific training modules. To enhance motivation, site coordinating committees should consider appointing individuals with specific roles to act as AMR surveillance champions in clinical (doctors, nurses or allied professions) including infection prevention and control, laboratory and data services.

- Teaching through formal pathways
- Onsite training at all levels
- Committees appointing specific champions

The sites selected, and the network as a whole, should reflect relevant variations in geography, socioeconomic factors and demography, disease epidemiology (e.g. co-morbidities such as HIV) and ecology, taking into account climate, rainfall and land use. Surveillance that only represents one level of healthcare (e.g. referral hospitals) will not adequately reflect the AMR situation of the country. The potential for biases include: 1) sampling only from referral hospitals, which may have high numbers of patients treated with antibiotics prior to sampling or high numbers of cases who have failed first-line treatment at referring facilities 2) sampling only from hospitals may under-represent less severe infections e.g. sexually-transmitted infections, uncomplicated urinary tract infections, community acquired pneumonia. 3) sampling only from healthcare outpatient clinics will result in under-representation of severe or invasive infection 4) health financing systems that require patients to pay for investigations will include only those who are able to afford investigations

- Sites selected should reflect variations for sampling
- Sampling through sites needs to be representativeness
- Adjusted for multiple biases

AMR surveillance sampling should therefore be drawn from the health facilities used by the population targeted for surveillance. These may include referral hospitals, district hospitals and out-patient facilities (including primary care); some institutions may fulfil more than one of these functions. Facilities serving a population sub-group, such as private hospitals in a country where most hospital services are delivered through the public sector, should only be included if the rest of the population is already adequately represented

- Surveillance drawn from facilities that population use for representativeness

To allow full and informative interpretation of data, effective AMR surveillance requires well-functioning health-systems that serve a defined population. Standard laboratory methods for pathogen identification and antimicrobial susceptibility testing are vital in order to understand the emergence of AMR and inform policy, but so too are population descriptors, healthcare utilisation patterns, and the systematic assessment and investigation of patients

- Standard laboratory methods neeeded to understand AMR

Population catchment and sampling frame. Wherever possible, the catchment population of the health facilities included in surveillance should be defined and an assessment should be made of the patterns of healthcare utilisation in that population. This is important for data interpretation: total population allows estimates of incidence and trends; descriptors define risk factors (socioeconomic status, urbanisation, co-morbidity levels) for national models of AMR burden; access to care patterns determine whether the healthcare facilities included are the first point of contact, post-treatment, or post-clinical failure level – which will have different AMR prevalence.

- Population catchment should be captured

Clinical surveillance. AMR surveillance data should be interpreted in the context of local clinical practice. This is particularly relevant for low-income country settings, which use syndromic management approaches where patients are diagnosed clinically and treated empirically.

- Interpretation of results need to be done in context of clinical practice

Isolate identification. Specimen culture and testing for antimicrobial susceptibility should be done by sentinel site laboratories. Isolates with unusual susceptibility profiles, or of uncertain identification, should be referred to the coordinating AMR laboratory, as well as a proportion of all isolates for quality control purposes. All isolates from blood or CSF specimens should be

- Identification of specimens done at sentinel sites

Saeed et al. 2017

Specific gaps identified in terms of quality assurance included lack of quality control of susceptibility discs and antimicrobial disc potency, non-availability of standard guidelines in the procedure manuals for inconsistent AST results, and the need for participation in internal and external quality assurance programs

- Quality control in susceptibility discs
- No standard guidelines for procedures
- Need for participation in quality assurance programmes

Amongst the laboratory cohort from Karachi, the initial on-site visits conducted after the workshop, revealed that the impact of the workshop was limited and that the participant laboratories only partially succeeded in implementing practices communicated during the workshops. Contributory factors included lack of resources e.g. access to standardized quality control strains for AST. Implementation of quality controls for media and antimicrobial sensitivity testing were also incomplete. The laboratories were not participating in external quality control program.

- Lack of physical resources
- No quality control for media
- No participation in quality control programmes

Due to limited spending on health by the government [18] monetary and personnel resources required to establish and maintain standardized laboratory practices are insufficient.

- Limited funding by government leads to limited resources
- Limited funding by government leads to limited resources

In particular limited use of quality control (QC) strains and standardized inoculum are pertinent gaps that undermine the reliability and reproducibility of AST being carried out. Additionally uninterrupted power supply in the public sector laboratories is a significant gap to be circumvented for improving laboratory infrastructure. These findings are consistent with earlier reports [20–22]. Low participation in AMR surveillance, a weak collaborative network between laboratories and insufficient use of Laboratory Information Systems (LIS) are underlying bottlenecks that need to be addressed in order to strengthen data collection, validation and aggregation of regional and national resistance data. LIS in particular is recognized to not only improve capacity for AMR surveillance through the collation of data from different laboratories but to also contribute towards standardization and improvement of the quality control of methodology [23].

- Limited quality control and standard inoculum
- Uninterrupted power supply
- Limited use of LIS needed for data collection
- Participation in network is low

While an earlier knowledge and practices (KAP) survey from Pakistan reports considerable gaps between awareness and implementation of standardized laboratory practices [24] comparatively higher scores of 54 and 72% for public and private laboratories, respectively, indicated in our study, suggest that implementation of SOPs has improved since the earlier KAP survey.

- Gaps in awareness and implementation of standardardised laboratory practices

In resource limited healthcare settings where national accrediting bodies exist, a higher percentage of accredited laboratories have been observed to belong to the private sector [25]. In such settings, private sector laboratories, have the potential to assume a pivotal role in combating AMR by partnering with public sector laboratories and by participating in regional or national surveillance to produce a clearer picture of resistance trends [25]. In contrast, data from our study indicates that participation in external quality assurance was weak not only in public laboratories (46%) but also in the private sector labs (38%). Paradoxically, despite a high participation of public sector labs in internal quality assurance programs (92%), prevalence of significant gaps in these labs reiterates that internal quality assurance can be more robust and effective when complimented with an External Quality Assurance System (EQAS).

- Partnership between private and public sector is important
- External quality assurance weak in all sectors

Knowledge based-interventions in the form of short courses addressing specific diseases, along with skill development have been proven to be effective models for laboratory capacity building [11]. Consistent with these findings, significant efforts by cohort laboratories towards addressing gaps; development and implementation of SOPs regular use of standardized quality control strains and standardized inoculum for AST emphasizes the value of knowledge-based interventions towards addressing laboratory gaps. The success of proficiency testing (PT) in conjunction with training programs has been highlighted by a number of studies from resource limited settings [26–29]. The approach of partnering laboratories fulfilling core capacity with weaker labs suggests a model of a sustainable network for knowledge and skill transfer for RLCs. It has been suggested that such partnering of laboratories may also contribute towards reducing costs and increasing the range of diagnostic facilities; enabling a robust laboratory system for surveillance of infectious diseases [30]. Furthermore, tiered laboratory networks along with clearly defined national guidelines that push for gearing lab capacity towards national accreditation can achieve remarkable improvements in laboratory diagnostic capacity for surveillance.

- Knowledge based interventions effective to improve laboratory capacity
- Standardisation through knowledge based interventions
- Partnering with lower capacity laboratories seen as successful in increasing diagnostic facilities
- Tiered laboratory network can be used to improve laboratory diagnostic capacity

Seale et al. 2017b

Leadership
Leadership, and the national policy addressing AMR, as well as international collaboration, are necessary to strengthen AMR surveillance in LMIC countries, to ensure an enabling policy environment. However expertise in microbiology is limited in these settings, and, whilst in the longer term this capacity is important to develop, in the interim leaders may need to be drawn from clinical infectious diseases and public health. Leaders are important for advocacy/championing, and for local credibility of the surveillance effort, as well as providing the focal point for activities

- International collaboratio needed
- Expertise is limited in LMIC settings
- Interim leaders needed for expertise

Training
Where health systems are weak and still developing, there is a need to develop human resources across cadres of staff: clinical, laboratory and data management. Well-trained staff are likely to move elsewhere for better salaries; it is important that there is sufficient budget to ensure staff can be recruited and retained.

- Need to develop human resources

Laboratory quality assurance
In terms of strengthening laboratory capacity, it is clear that inadequate laboratory infrastructure limits the quality and the ability to reliably detect pathogens and conduct antimicrobial susceptibility testing

- Laboratory infrastructure is limited and affects quality of microbiological investigation

Overall, a model with active, continuous, comprehensive integrated population and laboratory disease surveillance would provide the most robust, comprehensive data. However, within the constraints of resources, an appropriately designed sentinel site system would also be appropriate. Whilst it would be feasible to assess AMR outside of a health care context, for example through cross-sectional studies of population colonisation (as part of a population based survey), these would not include surveillance of the most serious drug resistant infections, and fall outside the remit of public health surveillance as observational research

- Active model of surveillance provides most robust and comprehensive data
- Constraints of resources, sentinel may be better

sent to the coordinating AMR laboratory for storage

Antimicrobial susceptibility testing for priority pathogens should be carried out in line with international standards, preferably according to the European Committee on Antimicrobial Susceptibility Testing (EUCAST) methodology and guidance (www.eucast.org). Where Clinical and Laboratory Standards Institute (CLSI) guidelines are used, these may also be reported. Unless automated systems are already in place, antimicrobial susceptibility testing at the core level should be performed using the disc diffusion method. Where additional drugs are included (for example Acinetobacter baumannii vs. carbapenems), they should be tested according to accepted guidelines (e.g. CLSI, EUCAST)

- Adoption of international standards for testing of isolates
- EUCAST or CLSI

Reporting results requires efficient data management at both sentinel site and national levels (Supplementary File 1: Figure 5). Quality control should be incorporated at every stage, with automated data validity checks and rules, as well as audit to check data consistency, completeness and accuracy. Confidentiality should be protected and data security measures should be in place (links to resources are given in Supplementary File 1: Appendix A).

- Data management is needed with quality checks for completeness and accuracy

At core level, clinical data should be recorded in a standardised paper request form that accompanies the clinical sample to the laboratory. Sites operating at extended level will capture data using an electronic system. The minimum set of data required on the core clinical request form are: age, sex, clinical diagnosis, specimen type, sample date, admission date, hospital or community source.

- Clinical data recorded in a standardised paper format
- Inclusion of metadata

Quality assurance. Quality assurance (QA) should be led by the national coordinator and technical team in country, in conjunction with external organizations as appropriate. At a core level, all site procedures should be undertaken according to SOPs, adapted from national SOPs, and based on these guidelines. Alongside these, quality control (QC) and QA procedures should be established to ensure that the data produced are accurate and reliable.

- Quality assurance should be lead by national coordinator
- Standardised operating procedures needed

studies requiring appropriate ethical approvals and individual informed consent.

At a national level, there is a need for leadership to support training, and quality across the system. A national AMR laboratory can support development of site laboratories and conduct more extensive testing (thus focussing resources, Supplementary File 1: Table 3, Figure 1). At a site level an integrated model includes development of laboratory capacity (isolate identification and susceptibility testing), clinical surveillance (systematic investigation of patients according to diagnosis), health care utilisation surveys (to assess representativeness of the health facility data) and census or enumeration data to determine the population catchment (Supplementary File 1: Table 2, Figure 1).

- National level requires support for training across system
- Site level requires training for laboratory capacity and testing

Malawi.
Malawi faces substantial challenges due to extremely limited country resources, which reduces health system capacity. However, whilst it does not have the capacity for a comprehensive AMR surveillance system, it has been developing institutional infrastructure and policy for AMR surveillance. It also has considerable capacity through international collaboration and an academic centre of excellence (Malawi-Liverpool Wellcome Trust (MLW)) undertaking microbiological surveillance as part of a research programme. In order to take advantage of available research infrastructure i.e. sentinel surveillance capacity beyond Queen Elizabeth Central Hospital (QECH), the following should be considered for new surveillance sites in Malawi:

- Malawai case study with limited resources increasing capacity through international collaboration

Ethiopia.
Ethiopia is developing a system for AMR surveillance that includes a national reference laboratory and sentinel regional laboratories across the country reporting data (continuous, passive surveillance). At present, whilst capacity is in development, there are some hospitals where the standard of clinical examination is adequate for surveillance, but there is limited microbiology laboratory capacity for processing specimens for culture, particularly blood cultures. Record keeping is not formalized or electronic, and there is potential for improved communication between the ward and the laboratory to link microbiology data to patients. In strengthening capacity for AMR surveillance, the following should be considered:

- Ethiopia another case study
- Potential in improvement with capacity

1. Determine whether the hospital(s) are epidemiologically representative It is important to include a range of health care settings for AMR surveillance; district hospitals, outside of the capital are more generalizable to the Ethiopian population as a whole than very urban settings. 2. Establish surveillance within the hospital Systems for development would be national guidelines supporting standardised clinical examination and systematic investigation. Laboratory capacity needs strengthening in terms of automated blood cultures to support AMR surveillance to a core level. Clinical and laboratory data should be linked, this could be through a double sided form with data collection for both. 3. Connect the site to a national and regional network The Ethiopian Public Health Institute is an arm of the Federal Ministry of Health and combines public health policy making with laboratory capacity in the same institution. Stronger regional links could support capacity development as well as continued partnership with international organizations

- Determine representativeness
- Establish hospitals sytems for development of national guidelines
- Linking data together
- Continued partnerships between organisations

Vietnam.
Vietnam has established national infrastructure for AMR and has worked with regional networks and international collaborators to develop a surveillance system, including a national reference laboratory and twenty laboratories attached to district hospitals. The hospitals are public and broadly representative, there are functioning microbiology laboratories and the physical, legal and ethical capacity to link these records for anonymised aggregation at individual case level. In strengthening capacity for AMR surveillance, the following should be considered:

- Vietnam has collaborated with regional and international networks
- Hospitals are broadly representative
- Link data together

Simjee et al. 2018

While both programs utilize the same AST methodology, namely MIC testing utilizing agar dilution or microbroth dilution techniques, there are major differences in breakpoint determination, types of bacteria screened, and interpretation. Within the animal arena, application of different terminologies, techniques, and clinical breakpoints versus epidemiological cutoff values (ECOFFs) has meant that it has been difficult to compare data across national programs, and sometimes within programs over time as breakpoints are re-evaluated or created and methodological advances made.

- Differences in breakpoint and determination
- Cut-offs are different which limits comparability across programs

However, there is often much more variability in AST methodology applied to the isolates, with many veterinary diagnostic laboratories still being heavily reliant on disc diffusion methodology because of its rapidity and price. Automated MIC testing is increasingly available in veterinary laboratories but is often an additional cost for the client. Therefore, AMR surveillance focused on animal pathogens still often relies on centralized collection of isolates by an independent reference laboratory, which then conducts "gold standard" AST (MIC testing), making this a very expensive option for regular ongoing surveillance programs. Where veterinary diagnostic laboratory testing is well coordinated and methodologically similar, such as in France's RESAPATH program, passive collection of animal pathogen AST data is possible and has generated some useful information for antimicrobial stewardship policy and procedures (see example below)..

- Variability in methodology for isolate testing
- Centralised collection of AST data is expensive
- RESPATH is an example of successful coordination

Data should be stored in secure databases that facilitate simple entry and retrieval, flexible reporting, and ad hoc analysis. Compatibility with similar national and international databases is important. Electronic transfer of data from other systems is highly recommended, rather than manual data entry, which is timeand resource-consuming and error-prone.

- Data stored in flexible databases

From the outset, DANMAP adopted a coordinated, One Health strategy; they developed a highly integrated, systematic, and continuous program that covered the entire food chain, relating antibiotic consumption with resistance, from "farm to fork to sickbed." Unique methods of integrating data were developed that created outcomes for action through cross-sector collaboration between scientists and authorities. DANMAP has been highly successful due to adequate funding, excellent planning, and collaboration at all sectors, but also because Denmark is a small country with a large economic reliance on high-quality agricultural produce (approximately 80% of antimicrobials used in the animal sector are administered to pigs) and relatively short distances between farms, processing facilities, and laboratories.

- DANMAP adopted a coordinated One Health approach
- Unique methods of data integration were created through cross sector collaboration
- Adequate funding

- RESPATH incoprorates data from 63 public and private

RESAPATH, operated by ANSES, the French Agency for Food, Environmental, and Occupational Health and Safety, coordinates the voluntary contribution of antimicrobial susceptibility data from isolates from diseased food-producing animals and companion animals obtained by 63 public and private diagnostic laboratories distributed throughout the country.

- laboratories across the country

CIPARS aims to provide a unified approach for monitoring trends in antimicrobial use and resistance in humans and animals and for facilitating assessment of the public health impact of antimicrobials used in humans and agricultural sectors to enable accurate comparison with data from other countries that use similar surveillance systems.

- CIPARS provided a unified approach to monitoring AMR in humans and animals

In looking toward the future of AMR surveillance, it is clear that the main way in which it will change is through greater use of DNA sequencing technologies. The development of affordable WGS technologies, along with complementary advances in bioinformatics, provides a single, rapid, and comprehensive laboratory procedure by which to characterize bacterial strains. The power of WGS for public health surveillance has already been demonstrated.

- WGS technologies may improve understanding of AMR

Singh-Moodley, Ismail, and Perovic 2018

Since surveillance of antimicrobial-resistant bacteria is performed only in a few African countries, there is a paucity of accurate and reliable information and consequently limited data concerning the true extent of the problem. An additional challenge noted during an external quality assessment of public health laboratories in Africa21 showed that many countries experience problems with performing antimicrobial susceptibility testing. From this external quality assessment, a question on the consistency and accuracy of antimicrobial susceptibility testing data arises. Furthermore, although some countries have established surveillance programmes, there is a lack of a formal framework across the region.

- Paucity in reliable information due to limited data
- Limited external quality assessments for laboratories
- Lack of framework across the region

For a national laboratory-based surveillance programme, government commitment and support is non-negotiable. This requires the drafting of policies and strategies and the securing of resources both, financial and human. GLASS participation also requires the establishment of a coordinating centre which will systematically collect, analyse and share data nationally and internationally. Participation also requires a regional reference laboratory which will provide technical support including training, capacity building and strategy advice

- Government committment is necessary for surveillance
- Drafting policies and financial resources are necessary
- Participation in GLASS needs coordinating centre

National Department of Health attributed the weaknesses of surveillance and reporting activities to a number of factors. These included the low number of trained microbiologists outside of major urban centres, limited funding, the lack of a national electronic prescribing system and lack of linkage to pharmacy, clinical and laboratory data systems in institutions resulting in incomplete and variably reported information on antimicrobial resistance and consumption.

- Weaknesses in surveillance
- Weaknesses in training
- Limited funding
- Lack of data linkages

In an effort to improve worldwide antimicrobial resistance surveillance, the Global Antimicrobial Resistance Surveillance System (GLASS) was developed to support the Global Action Plan (GAP) on antimicrobial resistance. It is coordinated with the national action plans of countries and aims at enabling countries to generate antimicrobial resistance data that is standardised, comparable and validated.

- GLASS has been used to coordinate NAPs
- GLASS allows for standardisation, comparability, and validation

Spiteri et al. 2013

In order to ensure comparability, these laboratories are assessed to ensure that they reach specific quality criteria which include performing consistently well in the EQA and good comparability between the laboratories' own national or regional susceptibility testing data and susceptibility data generated by centralised susceptibility testing. The implementation of decentralised testing is supported by an annual training course offered to laboratory technicians in participating countries. The establishment of a hybrid system of testing with centralised and decentralised testing has allowed shifting the focus to capacity building and training of experts in countries which do not yet participate in Euro-GASP.

- Comparability in data through EQA and centralised susceptibility testing

The Euro-GASP training component aims to increase the number of laboratory experts who are able to perform internationally validated and quality assured antimicrobial susceptibility testing across EU and EEA. This is needed because many EU/EEA member states are moving towards nucleic acid amplification testing and the skills for culture and antimicrobial susceptibility testing are being lost. The training is organised annually over 3 days to give participants a good working knowledge and understanding of N gonorrhoeae diagnostics, culture, species identification and antimicrobial susceptibility testing. The training can also be used to address issues arising from the EQA.

- Training for members as skills for culturing being lost
- Training addresses issues from EQA

Tornimbene et al. 2018

GLASS supports the strategic objective of WHO's Global Action Plan on antimicrobial resistance to strengthen the evidence base.2 GLASS provides a standardised approach to the collection, analysis, and sharing of antimicrobial resistance data by countries, and seeks to document the status of existing or newly developed national surveillance systems.3 GLASS is supported by WHO Collaborating Centres, involving strong commitment from participating countries and close collaborations with regional networks.

Following the call for country enrolment in March, 2016, and as of Dec 14, 2017, more than a quarter (50 countries) of WHO member states at different stages of economic development are enrolled in GLASS. The rapid increase in country enrolment and active participation in a global system to monitor resistance reflects a collective understanding and engagement to support the global effort to control antimicrobial resistance. By the end of the GLASS first data call on July 8, 2017, 40 countries provided information about their antimicrobial resistance surveillance systems and 22 provided 2016 data for antimicrobial resistance, out of 42 countries enrolled.

- Following call, rapid participation in global system

Because this submission was the first year of GLASS data collection, great variability was expected in the completeness and quality of antimicrobial resistance data, and differences were addressed to promote a harmonised representation of the results and to show country efforts. For this reason, to avoid misrepresentations of the epidemiological status of global resistance, comparison of antimicrobial resistance results between countries or regions was not attempted. However, the surveillance standards established by GLASS proved to be a valuable and feasible method for future development, and represented a major achievement for both participating countries and GLASS.

- Great variability in completeness and quality of data
- Haromonization of data
- Comparability of regions cannot be done due to differences in data

Vernet et al. 2014

The diagnosis of resistance to antimicrobial drugs has so far relied on culture techniques performed in reference centers; these procedures have a long turnaround-time, are technically demanding, and are sometime dangerous.

- Diagnosis timely
- Technically demanding

The recent implementation of molecular tools for the diagnosis of M. tuberculosis infection and resistance in under-resourced countries illustrates the importance of integrating these tools with traditional diagnostic methods.

- Integration of new diagnostic tools with traditional methods were successful

A surveillance network, coordinated by the Malaria Research Training Centre (University of Bamako, Bamako, Mali), has taken advantage of rapid molecular-based tests that have been adapted for field use. It relies on health care workers, who have limited clinical training. After a short training course, they are able to collect blood samples from finger pricks and spot them onto filter paper strips that are sent to regional sites that detect PfCRT 76T, the key mutation causing chloroquine resistance.

- Malaria Research training centre has overcome training gap through running short course

Key factors that contributed to the success of this health information system included molecular methods and the reliability of the data-sharing system. Internet access through satellite connection, which became possible a few years ago, will allow more timely data centralization.

- Successes to information system included data sharing system and internet connection

Lessons learned from antimicrobial drug resistance surveillance highlight the need to endow surveillance networks in under-resourced countries with laboratory capacity in a sustainable manner—infrastructure and human resources are required to obtain reliable data that can inform both clinicians and policy makers. Common problems should be addressed first: the death of bacteria during shipping, lack of standardization and guidelines for preparing culture media, high rates of culture contamination, and the lack of availability of diagnostic tests matching the most common infectious agents in circulation in a given area

- Laboratory capacity requires funding
- Human resources as well as infrastructure needed
- Standardisation of guidelines
- Microbiological procedure failings
- Microbiological materials

Training, feedback, and the introduction of a few simple hygienic measures implemented over the past 3 years in the pediatric service at Kilifi District Hospital have resulted in a reduction of the rates of contamination in blood culture samples from children from 19% in January 2009 to 5% in July 2011 (S. Morpeth, unpub. data). Good coordination between clinical and laboratory staff was crucial for this achievement, but the backing of the hospital senior clinical management staff was also necessary. However, the effort required to achieve similar results in standard district hospitals will obviously be more intense than it was at Kilifi District Hospital, which is part of a major research program. Clinical demand and political will are critical for the development of adequate laboratory services.

- Training and feedback implemented has lowered rates of contamination
- Done through coordination between clinical and lab staff

Existing infrastructures in under-resourced countries encompass ingredients for good surveillance of antimicrobial drug resistance, but the bottleneck is laboratory capacity. This capacity needs to be built into all levels of the health care system. Planning should take into account the following: tools to be used for diagnosis, sustainable funding, sharing of standard operating procedures, data management, a central database, and resource centers. In addition, logistical and human issues should be considered: for example, common procurement strategies, training of technicians, communication to raise awareness of antimicrobial [illegible]

- Under resourced surveillance system
- Materials are needed for microbiological surveillance
- Planning for standardised procedures, data management [illegible]

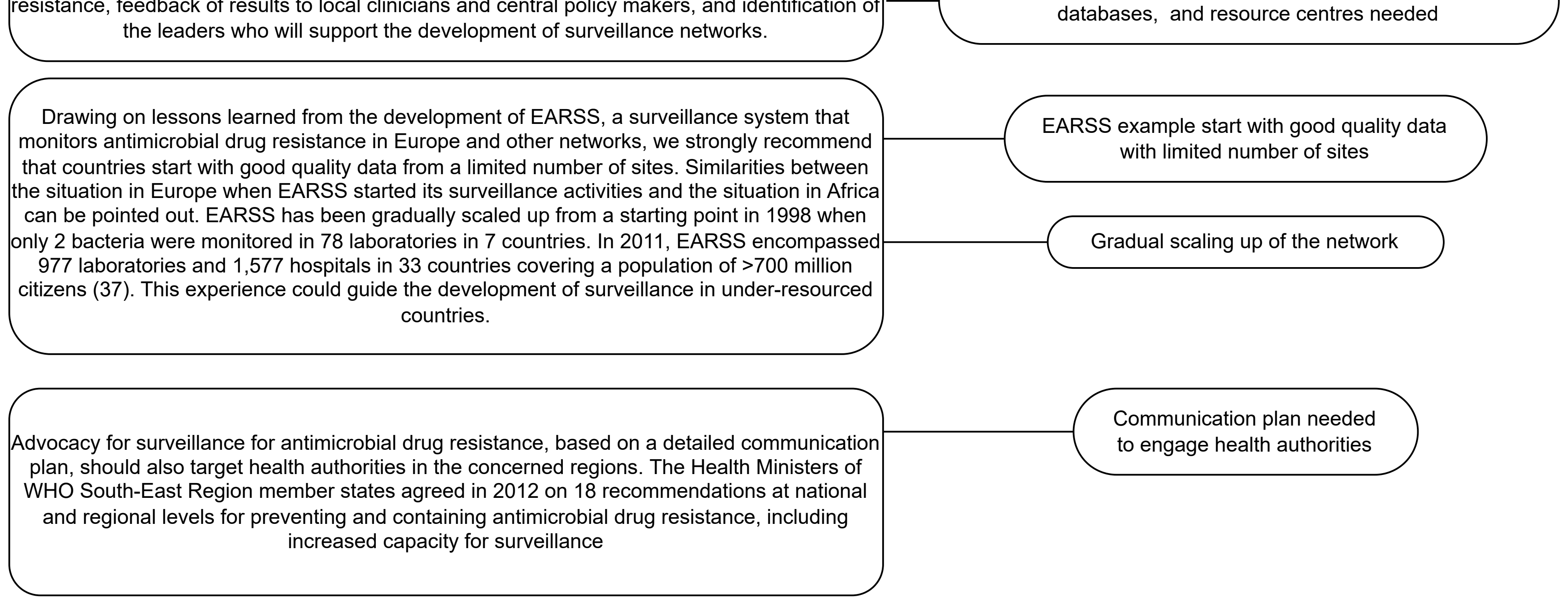
resistance, feedback of results to local clinicians and central policy makers, and identification of the leaders who will support the development of surveillance networks.
databases, and resource centres needed
Drawing on lessons learned from the development of EARSS, a surveillance system that monitors antimicrobial drug resistance in Europe and other networks, we strongly recommend that countries start with good quality data from a limited number of sites. Similarities between the situation in Europe when EARSS started its surveillance activities and the situation in Africa can be pointed out. EARSS has been gradually scaled up from a starting point in 1998 when only 2 bacteria were monitored in 78 laboratories in 7 countries. In 2011, EARSS encompassed 977 laboratories and 1,577 hospitals in 33 countries covering a population of >700 million citizens (37). This experience could guide the development of surveillance in under-resourced countries.
EARSS example start with good quality data with limited number of sites
Gradual scaling up of the network
Advocacy for surveillance for antimicrobial drug resistance, based on a detailed communication plan, should also target health authorities in the concerned regions. The Health Ministers of WHO South-East Region member states agreed in 2012 on 18 recommendations at national and regional levels for preventing and containing antimicrobial drug resistance, including increased capacity for surveillance
Communication plan needed to engage health authorities
