# Supplementary material for: Strengthening antimicrobial resistance surveillance systems: a scoping review
Source: BMC Infect Dis. 2023 Sep 11;23:593. doi: 10.1186/s12879-023-08585-2 (PMC10496311; doi:10.1186/s12879-023-08585-2)
Supplement: Supplementary file 3 — Supplementary Material 3 [file 12879_2023_8585_MOESM3_ESM.pdf]

Overaching themes

Legend: Supplementary file 3 shows the aggregation process of activities into larger themes

Capacity

- Physical personnel
- Laboratory Infrastructure
- Education and training
- Materials

Data infrastructure

- Data
- Microbiological standards
- Quality
- Reporting of results

Stakeholder engagement

- Collaboration
- Harmonisation
- Reference laboratory

Policy

- Government
- Political
- Committments

Representativeness

- Bias
- Surveillance system structure
- Participation

Sustainability

- Financial
- Longevity of surveillance

Capacity

The overarching theme that governs the physical operations of the surveillance system was given the name capacity. It encompasses the determinants for surveillance system function throughout its infrastructure, personnel, education, and physical materials needed for microbiological investigation. It explicitly refers to the physical "capacity" for which surveillance can operate.

Policy

Policy was given the overarching theme for government, political, and committment themes as this was the end result of all of the themes included. The common goal for these themes were to influence and create policy so that it was conducive to the operation of antmicrobial resistance surveillance.

Data infrastructure

Data infrastructure was the encompassing theme for all themes that were data-related. This included the reporting, interpretation, quality, and collection of data. The inclusion of infrastructure was pertinent as the discussions within data also pertained to how data was captured and intrepreted. This was done through data infrastructure platforms which are integral to discussing antimicrobial resistance surveillance.

Representativeness

Representativeness refers to how accurately surveillance captures antimicrobial resistance. Bias, surveillance system structure, and participation are components to looking at representativeness. Bias refers to the potential biases that must be contended within in surveillance. Structure and participation look at whether the system has enough breadth to do so.

Stakeholder engagement

Stakeholder engagement was designated the overarching theme as this was the common goal amongst the themes of collaboration, harmonisation, and reference laboratories. It involved multiple parties as the intracies involved in carrying out a single objective together. This often included engaging "stakeholders" of the surveillance system.

Sustainability

Sustainability was the common goal amongst the themes of financial and longevity of surveillance. They are both determinants in the sustainability of surveillance endeavours and have significant overlap in their core ideology.

Emerging themes from codes

Data

- Missing data
- Data completeness
- AST error prone outside quality laboratory
- Linking data together
- Accessing accurate data is complicated
- Data break point on surveillance
- Consistency in isolate data
- GLASS protocols to standardise AMR data processing
- Data sources
- Difficulty to differentiate between susceptibility
- Data timeliness
- Data can be easily extracted using softwar like WHONET
- Insufficient comparable data to develop global mapping
- Comparability of results/uniform test
- Data entry limited
- Merger of data
- Data not accessible by neighbours
- Complementing laboratory data with WGS
- Global systems reliant on national data
- Data sharing restrictions
- Does not use unique patient identifier
- Data quality is poor and incomparable
- Standard operating procedures for Bio-banking
- Data completeness and consistency
- Quality AMR data needs infrastructure
- Standards are not the same
- Availability of electronic data collection
- Data is standardised and reproducible
- Systems are fragmented in data capture
- Accessing accurate data is complicated
- Increase proportion of accurate data points
- Data is siloed and restricted
- WHONET used to capture microbiological data
- GLASS cannot accept data from pharma or research
- No homogenity in reporting of content
- Individual solutions for data entry integrated with existing practices and laboratory staff and management
- WHONET is an example software to facilitate sharing of AST data
- SOP has developed guidelines to standardise data management
- AMR data is heterogenous
- Lack of data supplemented by national surveillance
- WHONET and BACLINK are programmes to facilitate database standardisation
- Data management
- Optimization data collection
- Data management done by working group
- National AMR reports lacking or not available
- No uniform reporting of AMR
- Problems in standaridsation of databases
- Linking of systems
- Integration provides more complete data
- Reporting and sharing data
- Effective data reporting
- Absence of data consensus
- Questionable quality of data
- EARS-Vet provides standardisation
- Statistics exist but not integrated.
- Coordination team developed standardised data harmonisation
- Limitations include data security, integrity, confidentiality, corruption in shared computation resource settings
- Data management need to be considered for scalability
- Weakness with unintegrated databases
- Data sharing and interopability
- Standardisation of data collection is needed
- Heavy laboratory workload leads to inconsistent data collection
- Data verification and highlighting isolates is complicated
- Data entry mistakes
- Standardization still being deliberated
- Inconsistent reporting of AMR data
- Data management and quality
- GLASS enables standardisation of data and reporting of AMR data
- Link data together
- Data is incomplete
- Data can serve to inform decisions
- Need for high quality data is there
- AMR and AMC data availability
- Sharing of data
- Limited data interpretibility
- Collect retrospective AMR and AMC data
- Limited availability of genetic data
- Good quality susceptibility data needed
- Data collection and analysis not standardardised
- Poor comphrension of data/analysis
- DANMAP - integration of data
- Poor questionnaire and data quality
- Innovation for new methods for data capture
- Public and private partnerships for data generation to be submitted to GLASS as national data
- Great variability in completeness and quality of data
- Standardisation of data
- GLASS allows for standardisation, comparability, and validation
- Paucity in reliable information due to limited data
- WGS technologies may improve understanding of AMR
- Data stored in flexible databases
- Data differences
- Data selection differences
- No standard guidelines for procedures
- Poor reporting of methodology
- Lack of data and standardisation
- Sufficient data is to be provided
- Feed-related data designed for feed-data and not product data
- Data acquisition is difficult
- Improving data quality
- End user data most pragmatic option
- Effective mechanisms for data sharing
- Response formats standardised for data collection
- Standardised data uploads
- Huge dataset produced
- Data limitations need to be recognised
- Data verification was problematic
- Data collection and management
- Difficulty in sourcing data
- Data cannot be compared
- Difficulty in data collection
- Private data not integrated
- Further integration by incorporating more data
- Substantial improvement with integration
- Data collection of agents, food, and disease
- Addressed data alimitations and integrated analyses
- Identification of risk clones by linking data

Data

Grouped together as the codes identified relate directly to the data generated by surveillance system. They detail issues and ideas directly relating to data

Microbiological standards

- EUCAST or CLSI
- Different procedures in WGS data
- Limited cohesive in investigative methods
- Globally recognised techniques and standards must be adhered
- CEESA applies mode uniform methodology
- Interpretation of results need to be done in context of clinical practice
- Concerns with standardised testing
- Standardised operating procedures needed
- Poorly updated guidelines
- No formal consensus on methodology
- Problematic as different laboratories use different procedures
- Investigations revealed lapses in hospital for microbiological services
- Need for robust, consistent criteria across sites
- Standardisation of case definitions
- Adoption of international standards for testing of isolates
- Provision of testing standards
- Standardisation of testing protocols and quality control
- Integration of new diagnostic tools with traditional methods were successful
- Pharmaceutical - susceptibility
- Standard laboratory methods neeeded to understand AMR
- Laboratories should use standardised methods
- No routine susceptibility testing of cases
- Definitions differ for resistance
- Standardised protocols in place
- Network with clear guidelines on reporting sampling and testing needed
- Laboratory assessment should look at AST for limitations
- Increases comparability across regions
- Standardisation with CLSI guidelines
- EU/EFSA/ECDC programme helps harmonization of results
- Limits comparability
- Agreement in susceptibility data to allow analysis
- Global standard
- Sampling protocols changed with revisions
- Better harmonization of sampling exists for specific species
- Lack of trust, demand and capacity for microbiological services
- Standardisation of methods
- Establish hospitals sytems for development of national guidelines
- Standardised procedures are needed
- Difference in quality and susceptibility results
- Lack of standardisation
- Standardization of epidemiological data accompanying WGS
- Methodological variations limits comparability
- Methods to improve quality and reduce microbiological testing are needed
- Centralised molecular characterisation
- Lack of standardisation
- Transition period when moving to EUCAST
- Diagnostic testing
- Internationally accepted guidelines needed
- Comparability of participating laboratories
- Differences in breakpoint and determination
- Unreliable blood culture systems
- Standardisation of laboratory techniques
- Barriers are lack of standardisation
- Inadequate standards
- Standardised methodology for susceptibility
- Lack of standards
- Standards are not uniform
- Consensus has yet to be made
- Standardisation of procedures and criteria
- EARSS for standardisation of criteria
- Standardisation of testing procedures
- CLSI standards for all laboratories
- WHO standardised laboratory methodology and protocols
- Standardised protocols throughout studies
- Criteria differ from country to country
- Guidelines increase data comparability
- Further need for standardised microbiological protocols
- Standardising data elements for inclusion on an antibiogram
- Adopting case definitions enable data comparisons
- Microbiological procedure failings
- Standardisation of guidelines
- Variability in methodology for isolate testing
- Cut-offs are different which limits comparability across programs
- Increases comparability
- Based on GLASS manual recommendations
- Non-uniformity of testing isolates
- Successful implementation with standardised and validated data
- International recognised standards
- Laboratory analysis
- Standardised case definition across all data
- Inclusion/exclusion criteria for samples
- Standardised systems
- Developing standard procedures in collaboration with scientific staff
- Developing standard procedures in collaboration with scientific staff
- Commerical blood cultures
- Establishment of standard procedures
- AST technician must interpret correctly
- Correct storage
- Heterogenous approaches
- Validation of selection criteria
- Interpretative criteria for resistance
- Integration points are different
- Comparability of data
- Adherence to testing methods
- Failure to distinguish strains

Microbiological standards

Codes grouped together as they relate to the microbiological services that are necessary to generate antimicrobial resistance data. This includes standards, guidelines, protocols, and analyses.

Political

- Successful AMR programme requires political support
- Essential for data governance agreements needed
- Mandates for developing an informatics solution for quality susceptibility testing and provides analytics
- Protect confidentiality but facilitate data sharing
- Cross-policy collaboration and support for research institutions is needed
- Design of dedicated policies
- Protects confidentiality
- Separation of activities
- Mandates must be critically aligned
- Lack of uniformity in AMR plan
- No mandatory reporting of prescriptions to collect data
- Written in policy by lacking in practice for data sharing
- Animal health law points out need for AMR data
- Sustainability needs political willingness
- Hindered by lack of political willingness
- NAP requires all laboratories to use EUCAST for AST.
- Limited influence on policy
- Several decrees have been adopted to promote AMR surveillance
- No legislated mechanism for collecting surveillance data
- Statutory requirement for veterinary pharmaceutical companies

Political

Themes discussing laws, agreements, and mandates were given the theme of political. This theme encompassed the legislative foundations for which antimicrobial surveillance could act upon.

Reporting of results

- Cases reported
- Record screening
- Trends with species
- Establish baseline for AMR burden
- Summarizing data is relatively straightforward
- Screening criteria
- Screening criteria
- Standardise measures for data reporting
- Leverage data in system and report on behalf
- Screening consistency
- Surveillance records
- Aggregation of multiple data helps to track burden and inform policy
- Industry, data confidentiality and reporting surveillance findings
- Inconsistent reporting
- Algorithm for detection
- Challenges to data collection, compilation, storage
- Time delay
- Multiple languages and cultural barriers to understand procedures
- Paper based data management was inconsistent for AMR data capture
- Smoother data pipeline with infrastructure
- Inconsistencies in panels
- Improve system efficiency of analyses
- Opted for different data entry solution
- Interpretation of results
- Limited metrics
- Diagnosis timely
- Aggregation of data is not standardised
- Hinder detection
- Results in delay and inadequate information
- Sharing of results is important
- Standardise data collection, analysis, customization and scability
- Data workflow standardisation with interpretation
- Standardised AMU storage system with mandatory fields must exist
- Slow turn around for results
- Programs are fundamental to standardisation of reporting
- Single language use for comparability
- Hetereogeneous timelines for reporting
- Reporting
- Can complicate uniform reporting
- Languages of reporting
- Establishment of quality assessment is necessary
- Hospitals reporting
- Faster results compilation
- Clinical data recorded in a standardised paper format
- Standardisation of interpretation of surveillance outputs
- Limited in the use to veterinary practice
- Optimal criteria for surveillance has not been established
- Standardise data exchange formats
- Improving capacity for assessing and reporting of AMR
- CAHSN system allowed for faster and more secure retrieval
- CIPARS provided a unified approach to monitoring AMR in humans and animals
- Inform decision making
- Incentives to improve sharing of data
- Inefficient data management and reporting
- Reporting delays
- Dissemination of resutls need standardisation
- Program not feasible as requires mandatory reporting
- Institute collects data from members
- Difficult to chart data
- Summary reports
- Complying with frequency of reporting
- High reporting rate
- GLASS ran by WHO collects information on AMR
- OH surveillance report

Reporting of results

This code was generated based on the reporting of data. This also includes interpretation of results by end users.

Education and training

- Laboratory personnel qualifications
- Education varied
- Staff need to be trained
- Technically demanding
- Clear need for educational programmes
- Microbiology staff training
- WHONET/Data training
- Laboratory mentorship in place
- Training for members as skills for culturing being lost
- Microbiological expertise
- Expertise is limited in LMIC settings
- WHONet software integration challenging
- Education to address sustainability gap
- Limited laboratory staff training
- Surveillance site personnel training
- Examples: management pathway by FAO
- Knowledge to be incorporated about all aspects of production
- Investment in training of personnel
- Onsite training at all levels
- Teaching through formal pathways
- Training of clinicians and laboratory staff
- Site level requires training for laboratory capacity and testing
- Staff training using Train the trainers approach
- Lack of expertise in microbiology
- EPHI trained and redistribute staff, sought laboratory mentorship
- Feedback to clincians post assessment
- Training and support needed to quality performance
- Knowledge based interventions effective to improve laboratory capacity
- Training workshops helped train local technicians
- Expertise needed for surveillance
- Continous education needed
- Not accustomed to inputting data in electronically
- Feedlot members are trained to capture data
- Training and academic skill may improve interdisciplinary training
- Free technical support to enable harmonized data
- Weaknesses in training
- Mentoring to assist staff
- Cross-sectoral training to increase collaboration
- Capacity building through mentorship
- Network based educative process.
- Better taining regarding techniques
- Training opportunity for staff for technical ability
- Training and feedback implemented has lowered rates of contamination
- Gaps in awareness and implementation of standardardised laboratory practices
- Training workshop for clinicians regarding microbiological clinical specimen collection
- Anonymous educative process
- Appropriate human resources needed with training
- Education and consultation
- Training and motiviation by staff important to build capacity
- Training programs helped to build laboratory capacity, standardisation
- National level requires support for training across system
- Laboratory capacity increased with education
- Mentorship and guidance
- Feedback to help educate laboratories
- Malaria Research training centre has overcome training gap through running short course
- Standardisation through knowledge based interventions
- Annual training of trainers program targeting facility level staff implemented
- Laboratory capacity and quality management through EQA, IQA, and accreditation
- Training of clinicians is an important aspect
- Mentorship promoted creative solutions to issues
- WHONET training implemented

Education and training

The theme was generated by the similarities discussed with education and training necessary for the use of surveillance. It includes training for the use of software, microbiological testing, increase in expertise, and laboratory training. The theme also includes challenges and successes with edfucation and training.

Quality control

- Quality controlled
- Adhere to quality assurance
- WHONET has data quality checks
- Training addresses issues from EQA
- Need for participation in quality assurance programmes
- Research data has rigorous quality assurance
- Quality assurance is questionable
- Data validation by appraisal
- Quality controlled
- Continuous for assessment
- Quality of data collection and interpretation
- Quality of data varies in private laboratories
- Quality management strengthening programmes
- No participation in quality control programmes
- External quality assurance weak in all sectors
- Data management is needed with quality checks for completeness and accuracy
- Limited QA benchmarking
- Quality control is of extreme importance
- Quality assurance not for antimicrobial use data
- Participation in quality assurance
- Quality assessment by reference laboratories
- Frameworks has enhanced data comparability and data with quality assurance
- Maintenance of QC testing
- Monitor surveillance capacity and performance
- External quality assessment
- Quality data assurance through EQA
- Tested and validation with EQA
- Low formal quality assurance
- Laboratories meet minimal quality standards required
- Irregular mentor visits
- Quality control to be addressed
- Repeat site visits to specify deficiencies
- [illegible]trol for media
- Limited external quality assessments for laboratories
- Lack of quality assurance and control
- Regular evaluations on the [illegible]
- Laboratory equipment was assessed at each site
- Lack of evaluative frameworks for systems
- Quality assurance essential
- Quality assurance to capture MDRO
- AMR networks - Latin American, non-European, and Central and East Asian networks have EQA schemes to laboratories
- Proficiency testing programs via Supranational networks
- Alternative methods for verification
- External quality assurance enrollment
- Internal quality control introduced
- To create national reference laboratory EQA, interal assurance, LIMS are needed
- Quality control in susceptibility discs
- Limited quality control and standard inoculum
- Quality control and assurance with feedback
- Facilitated standardised and quality assured
- Assurance program.
- Enrolment into EQA
- Inefficiencies in quality assurance
- EQA for laboratories needed
- Quality assurance is important for laboratories
- Laboratory quality management systems needed for quality control
- Technical committee conducts quality assessments
- Staff still undergoing training on microbiology skills
- Limitations in recovery of pathogens
- Comparability in data through EQA and centralised susceptibility testing

Quality control

Quality control emerged as a theme with the frequent mention of assurance programs. These assurance programs were similar in by their desire to appraisal and validate data. This included quality assessments that were both internal and external, as well as issues and challenges that arose with them.

Financial

- Sampling limiting due to finances
- Limited financial support to undertake collaborative work
- Cost of adequate surveillance
- Payment for data has been effective
- Hospitals have emergency funds
- Collaboration with stakeholders and external funding bodies
- EU funding of activities
- Funding by joint programme initatives
- Donor funds to support local planning and initatives
- Financial costs
- Financial and human resources in coordination team recommended
- Regional grants in Africa for funding
- Difficulty in sourcing funding
- Funding by VMD
- Organisation of regional programmes create opportunities to concentrate expertise to reduce cost and increase efficiency
- Limitations due to budget
- Due to lack of financial resources
- Limited funding by government leads to limited resources
- Management of participating hospitals increased budgets
- Provide human and financial resources
- Diagnostic capacity limited by time and financial costs
- Costs associated
- Wellcome data institute funding
- Utilise current funding through United Nations agencies
- Examples included Fleming fund
- However, cost benefits for one health implementation
- Financial support needed to build local capacity
- Wellcome, UK fleming fund and Gates foundation for funding
- Centralised collection of AST data is expensive
- Investments needed to improve microbiological quality
- Sustainability
- Fleming fund, Wellcome fund, are examples
- Adequate funding
- Compensation of maintaining surveillance is needed
- Funding helps with provision of quality data and address basic bacteriology services in low resource settings
- Testing not readily available due to high costs
- Initial costs associated
- Dedicated funding needed or improving system will be a challenge
- AMR fund will help with investments by pharmaceutical industry
- Fleming fund used to strengthen AMR surveillance in LMIC
- Low cost method to use existing data to increase AMR surveillance
- Wellcome Sanger Institute already has a initiative for publication
- Investment into AMR surveillance
- Limited funding
- Financial costs
- Laboratory capacity requires funding
- Engagement with commodity specific ERP
- Compensation for time and effort for AMU information
- Collapse due to funding
- Sustained funding from policy-makers
- Funding needed for data collection
- Limited funding by government leads to limited resources
- Global networks are sponsored
- Commodity groups and ministries which led to pilot surveillance
- Funding sources
- Global initiatives can be facilitated by funding

Financial

The emergence of finances as a theme was due to the similarities in discussion regarding funding of surveillance activities. This theme encompasses the mentions of the monetary aspects of surveillance through external funding, internal funding, budgets, or cost-benefits.

Information technology

- Internet connection/speed
- Information systems support microbiological data verification unaffordable
- WHONet software used with MoH coordinating AMR data validation
- Physical infrastructure in computers
- Open access applications such as WHONET and AMASS to generate reports
- Software integration will provides local surveillance infrastructure
- No computers to use
- Laboratory information systems are limited
- No sites have electronic laboratory information systems
- WHONET integrated with existing laboratory information systems
- Need for laboratory information system (LIS)
- Information systems are paperbased
- Sharing information using WHO-NET
- Technological problems
- Platforms can bias data
- Lack of data linkages
- Limited use of LIS needed for data collection
- Limited IT capacities of laboratories
- Technical challenges to retrieve data
- Data entry through WHONET and analyse data
- Commerical platforms increase standardisation
- Time challenges to retrieve data from LIMS
- Electronic data collection using paperbase
- Systems are not interlinked
- Successes to information system included data sharing system and internet connection
- Leveraged existing infrastructure to integrate other systems
- Use latest technologies
- Electronic systems give better data quality
- Electronic data collection used
- Laboratory information systems reduce workload errors and help standardise data reporting
- Electronic surveillance systems increase timeliness
- Data sharing platforms help support data collation, analysis and reporting
- AST results entered into WHONET
- WHONET software allows for standardisation
- WHONET has assisted in data collection, collation, and analysis
- Proprietary software
- WHONET software for data integration trialed but challenging
- Platforms are sufficiently user friendly to facilitate that
- Electronic submission of data
- Data management software needed

Information technology

Similarities in codes that discussed the use of software, computers, electronic data and linkage of data bases were similar in the means for which they are conducted. This led to the emergence of information technology which is the platform for the aforementioned activities.

Materials

- Resource limited laboratories
- Weak supply chains
- Procurement of materials
- Successful AMR surveillance requires access to good-quality reagents, inadequate storage, reagents and frequent power
- Limited resources
- Hindered by lack of resources
- Low diagnostic supplies
- Lack of resources
- Laboratory experts should be involved in ordering supplies
- Procurement
- ASM purchased supplies.
- Transport mechanisms to be established
- No intermediate culture set up
- Lack of resources leading to inaccurate results
- Limited resources to facilitate cross-sectorial collaboration
- Supply chain issues with climate
- Obtained long term supply of materials
- Low positivity rate due to transport times and sub-optimal swabs
- Large amounts of paperwork, limited supplies and poor equipment
- Need for increases resources to enable collaboration
- Microbiological materials
- Limited access to supplies
- Under resourced surveillance system
- Challenges in acquiring supplies and resources to perform tests
- Supply chain must be robust and adapted
- Inadequate resources and weak supply chains
- Local procurement of microbiology supplies is a challenge
- Maintaining power supply chain and cold chain remains challenging
- Procurement of materials
- Generally, material unavailable.
- Logistics and shipment may be a barrier
- Shortage of items and substandard materials are an issue
- Innovative partnership created to transport specimen
- Blood containing media unavailable
- Lack of physical resources
- Climate affects quality of consumables and diagnostics
- No established procedures for specimen transport
- Addressing logistic issues
- Adequate infrastructure including reliable equipment sustainable supply of quality consumables
- Lack of consumables for microbiological investigation
- Materials are needed for microbiological surveillance
- Expired material used but not appropriate
- Materials need to be good quality
- Working group created list of essential reagents
- Cost benefit and logistical issues addressed

Materials

The similarities in the discussion regarding procurement of reagents, supplies, and media led to the emergence of materials as a theme. This theme encompasses discussion regarding physical materials needed for surveillance and microbiological investigation. It includes discussion with supply chains, quality, and issues that may arise from these processes.

Bias

- Bias in surveillance data
- Epidemiology of species
- Online network would provide more recent data
- One health approach increases complete view of AMR
- Hospitals are broadly representative
- Characteristics not taken into account
- Sampling restrictive after one treatment failed
- Often no meta data
- No details in sampling
- Surveillance in veterinary field
- Geographical centres not reported
- Data collection limited to phenotypic
- Integration of private data sources
- AMR levels can be overestimated
- Poorly protected from bias
- Identification of risk factors
- Improved understanding
- One facility sent more than 50% of all samples
- Limited sampling bias
- Accuracy in data
- Representativeness of data
- Origin of infection challenge
- Remain private until published
- Represent multi-sectors
- Lack AMR data from clinical isolates
- Concerns for sampling
- Difficulty for representativeness
- Data that is undisclosed
- Duplicate samples
- Inclusion of metadata
- Animal sector not appreciated
- Horizontal transfer rarely monitored
- Veterinary sector limited
- Data from sites
- Data may be biased
- Exclusion of samples
- 2/3 of data from one locality
- Sampling bias
- Multiple counting is a significant issue
- Data completeness
- Integration of WGS improves representation
- Adjusted for multiple biases
- Over-representativeness
- Bias in sampling which may have limited representativeness
- Sampling bias present in reporting
- Data differes across sectors
- Population catchment and pattern of healthcare utilization not present
- Inclusion of meta data adds considerable value to laboratory information
- Guidance for sample selection and transport of specimens is absent
- Sales data can over-represent
- No explicit statement on how samples were selected
- Population catchment should be captured
- Sampling through sites needs to be representativeness
- Representativeness a limitation
- Covers 14 countries
- Not randomly selected
- Variation in AMR system
- Surveillance drawn from facilities that population use for representativeness
- Sites selected should reflect variations for sampling
- Meta-data from clinical systems are absent
- Comparability of regions cannot be done due to differences in data
- Approach provides high quality representative data
- Coverage for livestock sectors
- Inclusion of private laboratories in networks
- Limited information on food producing animals
- Prescribing data has limitations
- Samples sent are relatively low with low completing rates
- Data not robust enough to account for variation
- Laboratory surveillance may also be biased
- Performing comparisons is limited
- Represented samples across countries is different
- CIPARS - random sampling strategy
- No environmental data
- Indicators for geographical coverage and understanding need to be established
- Comparability of data, representativeness needs to be assessed
- Representativeness of this surveillance system
- Not all isolates tested for resistance
- Sampling biases linked to national surveillancew
- Lack of meta-data for drivers of AMR
- Further complications when sold through same channels
- Provides more complete picture of AMR
- Isolates may not give a clear representative perspective of the global danger of resistance
- Samples from persistent patients
- Sampling bias in surveillance systems
- Determine representativeness
- Small sample sizes for some samples types
- Data from sick animals leads to high estimates of AMR
- AHC isolates more likely to originate from individuals that are sick
- AHC relies on passive sample procurement
- Different subtypes have impact AMR prevalence
- Limitations to convenience sampling
- Convenience sampling used
- Random sampling should be used but not standard practice
- No standard definition to remove multiple counting
- Only male urethral samples
- Lack of consensus to appropriate AMU indicators
- Bias associated with the quality of the data
- Coverage of LMIc varied
- Does not include data from non-member companies
- Representativeness of AMR data is a challenge
- Low coverage
- Representativeness of the sample
- Challenge in coverage
- Surveillance should generate representative high quality data
- Coverage poor in high burden countries
- Pharma may not produce representative data
- Lack of data representativeness

Bias

The emergence of bias a theme was found with the simliarities of discussion points which focused on survey design elements. THis includes meta-data, sampling bias, over- and -underestimation. It also includes explicit mentions of bias. Issues and challenges that are associated with bias have also been included within the theme.

Committment

- Troubled committment
- One Health approach
- One Health approach
- Inclusion of multiple sectors in approach
- One Health approach at a institutional, national and regional level
- Need direction from national senior management
- Dependent on the objectives of surveillance
- Food, animals, and human surveillance should be adopted
- Training in AMR surveillance and lack of commitment by facility management
- Advisory group has representatives from science and policy
- Limited scope of surveillance
- Site coordinating commitee
- Government commitment is crucial for AMR surveillance programmes
- Need to improve and launch coordinated surveillance
- Support research and innovation
- Difficulty in recommended frequency of surveillance
- Objectives of surveillance need to be clear
- Roles for stakeholders need to be specific
- Laboratories are not ready to lead surveillance
- DANMAP adopted a coordinated One Health approach
- Lack of engagement
- Funding of AMR surveillance requires budget commitments
- Should have clear objectives
- No agreements between to collection identifiers
- Commites have overseen the implementation of AMR surveillance
- Higlighted in AMR strategy and comittments
- Success dependent on commitment and funding
- Shows participative approach can succeed
- UK OH report aligned data across sectors
- Support laboratory capacity for those who do not have it
- Current limitations in the system addressed by developing a common vision for AMR surveillance
- ResAlert contingency plan

Committment

Committment was a theme that emerged from the discussion of objectives, approaches, and plan for surveillance. These were encompassing aims of the surveillance system set out by stakeholders. Issues and challenges with committment have also been included within the theme.

Surveillance structure

- Identification of specimens done at sentinel sites
- One reference laboratory with competence is needed
- Improvement resulted in rapid surveillance scale up
- Variation in surveillance systems
- Expansion of networks
- Laboratory based may be most efficient
- Different sectors encompassed
- Hybrid solution in partnership with laboratories and hospitals
- Single site development with future expansion
- Sentinel surveillance to generate reliable accurate data
- Sentinel surveillance system with stepwise expansion recommended
- Population, sentinel, and laboratory
- Difficulty in routine implementation
- Partnership initiatives to expand surveillance
- System should be assessed to identify gaps
- Different approaches to surveillance
- Expansion of surveillance through sentinel sites
- Tiered laboratory network can be used to improve laboratory diagnostic capacity
- Together these laboratories form a netweork
- Not sensitive enough to detect emerging resistance mechanisms
- Designed with pyramid model with London School of Hyigene road map
- EARSS example start with good quality data with limited number of sites
- Cannot replace continuous surveillance
- Gradual scaling up of the network
- Difficulty in method integration
- Constraints of resources, sentinel may be better
- Both be considered in designing surveillance prrograms
- Planning for standardised procedures, data management databases, and resource centres needed
- Active model of surveillance provides most robust and comprehensive data
- Barrier to integration of additional laboratories

Surveillance structure

The theme surveillance structure was created due to the similarities of codes that discussed how the surveillance system should be structured.. This includes discussion regarding the expansion and growth of the issues as well as benefits and limitations of each structure.

Physical Personnel

- Barriers (challenges) by staff
- Needed for trained professionals
- Lack of human resource capacity for microbiological susceptibility
- Limitations in human resaources
- Human resources as well as infrastructure needed
- Dedicated personnel for data entry
- Look at gaps in laboratory capacity and staffing
- Inadequate trained staff with expertise and experience
- Need to develop human resources
- Lack of personnel
- Human resource capacity is most challenging
- Interim leaders needed for expertise
- Data entry person not trained
- Staffing must be sufficient
- Lack of established professionals in the field
- Strong leadership
- New pool of technicians
- Need for personnel engagement
- Internal management issues
- Lack of framework across the region
- Poor management are challenging barriers
- Need for trained personnel
- Strong staff skills helps capacity
- Staff capacity for laboratory is a challenge
- Fewer staff for culture and inputting of data

Physical personnel

The theme emerged when there were similarities within articles that discussed the need for human resources. This includes trained experts for surveillance and staffing of laboratories.

Government

- Determine their own organizational structures
- Appropriate reporting to national body
- Governance structure follows One Health approach
- Communication plan needed to engage health authorities
- UK government - priority in strategy
- Develoment of government structures
- Mandates by national government
- EGASP lead by MoH
- Activities are complementary to national plans
- Limited government committment
- Government has responsibility for gap analysis
- Government support
- Inadequate health governance
- Difficulty in engaging goivernment
- Priorisation of health by governments as a mechanism
- Call upon government engagement to improve laboratory capacity
- UK wide plan
- Limited involvement, lack of governance and leadership
- GLASS has been used to coordinate NAPs
- Drafting policies and financial resources are necessary
- Commitments from governments
- Government committment is necessary for surveillance
- MoH leads to sustainability of surveillance activities

Government

Government was constructed as a theme as there was centrality in discussions regarding the involvment of ministries and wider government within antimicrobial resistance surveillance. This includes strategy, political willingness and approaches to surveillance.

Participation

- Local laboratories do not benefit from participation
- Representation from other committees
- Supranational European network
- Multitude of stakeholders
- Uganda enrolled into GLASS
- Capacity to conduct surveillance in the multi-discplinary analyses
- Utilise partners
- Lack of awareness and utilisation of laboratories
- Increase participation
- Limited by participation
- committed to sharing data amongst partners
- Participation in network is low
- Already exist but voluntary with contributions
- Producer willingness to provide accurate AMU data
- Agreeing on principles for contribution
- RESPATH incoroprates data from 63 public and private laboratories across the country
- Following call, rapid participation in global system
- Improvements through increased number
- Encouraged participation
- WHO resolution calls for participation
- Volunteer nature of the system
- 30 organisations involved in surveillance
- European and global contribution

Participation

Participation was constructed as a theme with the simliarities in calls for participation in surveillance endeavours and networks. This also included the participation of external parties within current surveillance systems. It includes the successes and barriers faced by surveillance systems to increase/address participation concerns.

Collaboration

- Committees appointing specific champions
- Providing feedback mechanism
- Ensure continuous engagement of laboratories and clinicians
- Private sector collaboration
- Stakeholder engagement with veterinarians
- Limited laboratory capacity assisted through working with neighbouring country
- Coordination by AMR centre
- Collaboration with international centres
- Consensus across sectors is needed
- Difficulty in engaging GP and pharmacists
- Gaps in communication
- Coordinator collaboration with stakeholders
- Engages in planning and development of performance indicators
- One Health surveillance should include collaboration
- Feedback from participants
- Communication issues
- Public and private partnership
- Vetenarians should be involved in the process to improve AMR stewardship
- Molecular typing and other procedures carried out at another reference laboratory with competency
- Programs on behalf of a consortium produce harmonised AMR data
- Collaboration with veterinary management groups
- Meetings engaged local experts
- Collaboration with other parties to support surveillance
- No unified approach to AMR between sectors
- Strong internal and external communication
- Effective surveillance should be coordinated
- Collaboration between WHO and SEAR
- Academic networks - clinical
- [illegible]se AMR awareness through collaboration
- Need for intersectoral collaboration
- Collaborative approach
- Administrations should coordinate system
- Barrier in collaboration
- Limited collaboration between sectors
- Address harmonisation at national level
- Liasion needed to increase mutual trust and to optimize the use tools
- Permission from pharmacies were difficult to obtain
- Poor communication with medical tream and laboratory
- International collaboration, Ohio State University
- DANMAP addresses holistic AMR
- Depend on international collaborations in LMIC
- Poor linkages throughout sectors
- Strengthen partnerships
- Collaboration between sectors
- Various sectors
- Collaboration between sectors
- Consortium for global standard
- Knowledge translation gap between parties
- Engaging experts
- Work with health economists
- Public private partnership to improve local laboratory capacity
- Public private partnership to improve local laboratory capacity
- No interface between clinician and laboratory
- Improve validity via utilization of herd veterinarian
- Done through coordination between clinical and lab staff
- Unique methods of data integration were created through cross sector collaboration
- RESPATH is an example of successful coordination
- Multidisciplinary teams needed for surveillance
- Works in collaboration with sub-committees
- Vietnam has collaborated with regional and international networks
- Continued partnerships between organisations
- Malawai case study with limited resources increasing capacity through international collaboration
- Collaboration as a bridge between sectors
- International collaboratio needed
- Partnering with lower capacity laboratories seen as successful in increasing diagnostic facilities
- Success in Uganda highlights feasibility of implementing AMR system
- Dialogue between coordinating centre and participants
- Partnership between private and public sector is important
- Improve networking between disciplines
- Headed by senior consultant
- Data also necessary from other sectors
- Mutually beneficial partnerships between centres
- Coordination at a central level with expert comittee
- Collaboration of industry and government ensured communication
- Partnerships between government, WHO and academia
- National microbiology network with reference laboratory incorporates all sectors
- Stakeholder meeting contributed in motivating MoH
- Success depended on extensive consultation
- Lines of communication were established between surveillance sites
- Inclusion of key stakeholders
- Communication plans between AC members.
- Georgia as an example to improve surveillance through CAESAR network
- Led by trained national project coordinators
- Surveillance network ran by ECDC

Collaboration

Collaboration is a theme that was developed from the discussion throughout the articles regarding engaging different parties and working together. This includes cross-sectorial, local level, coordination efforts, and consortiums that have been made to run surveillance.

Laboratory infrastructure

- Infrastructure availability
- Use existing infrastructure
- Varying capacities across health facilities
- Laboratory infrastructure is limited and affects quality of microbiological investigation
- Lack of infrastructure
- Inadequate laboratory infrastructure decreases data quality and reliability
- Quality of water, electricity, light sources, climate control, internet, ventilation, covarge, soap, toilet, construction
- Documents produced for surveillance foundation
- Lack of laboratory capacity
- Laboratory capacity built over time
- Laboratory capacity focused on providing reliable surveillance data
- Uninterrupted power supply
- Required physical infrastructure needed for assesment
- Lack of laboratory infrastructure
- Improves surveillance through decrease of workload and increase laboratory capacity
- Surveillance requires diagnostic capacity of laboratories
- Physical deficiencies in water, electricity, and infrastructure
- Requires high investment in hardware
- Development and improvement of laboratory capacity needed
- Do not have appropriate equipment
- Infrastructure is limited
- Laboratory equipment installed
- Limited laboratory capacity in LMICs
- Increasing number of microbiology testing facilities, strengthening local capacity and existing laboratory systems
- Limited and fragmented capacity for AMR surveillance
- Barriers in capacity
- Laboratory tools are deficient
- Implementing surveillance difficult due to size
- Weak laboratory infrastructure
- Success in building surveillance system
- Lack of ultility in peripheral centres
- Suboptimal microbiological laboratory capacity
- Establishment of EGASP shows success in resource limited setting
- VMD developed collaborative antibiotic collection systems
- Successful AMR surveillance requires access to good-quality reagents, inadequate storage, reagents and frequent power
- Increase of capacity for diagnostic capacity

Laboratory infrastructure

Laboratory infrastructure relates to the physical infrastructure that is needed for a laboratory. This includes planning, physical materials for laboratories, power, water, and electricity.

Longevity of surveillance

- NCC looks at sustainability
- Low return on investment
- Sustainability of project when involving multi-disciplinary teams
- Sustainable funding for upkept activities
- Shifts in reducing microbiological costs needed
- Support research
- Heavy reliance on external funding
- Sustainable funding needed
- Reliance on external funding
- Sustainability of endeavours because funding is short term
- Cost-benefit needs to be considered
- Short term funding and the need for long term sustainability
- Sustainability with continued training
- Governments reminded surveillance is cost effective

Longevity of surveillance

The theme longevity of surveillance was idenitified within the discussion of sustainability of surveillance. It was primarily focuses on the continued endeavour of surveillance and mechanisms which will allow that to happen. It includes criticisms regarding funding, need for research, and examination of sustainability as a concept

Harmonisation

- Further harmonisation of efforts
- Harmonization of procedures
- Agreements
- Harmonize practices
- NIVM, RIVM and stakeholders developing standard
- Harmonisation when standards are defined in a bottom up approach
- ARSRL coordination
- Haromonization of data
- Companies providing aggregated data
- Harmonize global standards to produce timely and comprehensive data
- SENTRY - JMI laboratories (global surveillance)
- Institution heads not signing
- Prioritization of objectives through consultation can help implementation
- Dissemination of information to stakeholders has no centralised approach
- Standardise surveillance using WHO guidelines
- Harmonization through Global Microbial Identifier and other International initatives
- Harmonization across labs possible

Harmonisation

Harmonisation was identified as a theme that primarily focused on the harmonisation of antimicrobial resistance surveillance. It includes coordination, initiatives, and approaches to harmonise efforts.

Reference laboratory

- Reference laboratory
- Reference laboratory coordinates activities
- Isolates tested in central laboratory
- NRL established a standardised data collection procedure
- Participation MML has contributed to knowledge and quality
- Data standardisation by centralised laboratory service
- NRL verified consumables
- NCC monitor quality assurance and training
- Coordinating AMR laboratory
- Successful in developing a national reference laboratory
- NRL continued training of members of the network
- Headed by National Coordinator
- Improve surveillance platforms
- Central leadership and budgeting is needed to enable capacity
- NCC convening a techanical team with strong government engagement
- Reference centre for isolate processing
- National reference laboratory provides EQA
- Responsibility given to national reference centres rather than supranational
- Quality assurance should be lead by national coordinator
- NCC should engage multi-sectoral stakeholders
- NCC supports policy, plans, and operating procedures
- National reference laboratory needed assistance to be a coordinating centre
- Establishment of coordinating centre for surveillance
- Coordination of system through reference laboratory
- Only 30% have national coordinating body
- Participation in GLASS needs coordinating centre
- Strong central institution helps define scope and procedures
- Centralised data platform for processing
- National reference laboratory created a support network to communicate with other laboratories
- Reference centres have responsibility but are not well equipped
- NCC oversees surveillance program
- National reference laboratory can interoperate and share data
- National Coordination Centre does data validation
- DARC group coordinates DEFRA activities
- Central laboratory needed

Reference laboratory

Reference laboratories were an emergent theme within surveillance literature. This theme explicitly encompasses all mentions of a national reference laboratory and the benefits and limitations it may have on surveillance efforts.
